# Performance test and verification of an off‐the‐shelf automated avian radar tracking system

**DOI:** 10.1002/ece3.3162

**Published:** 2017-06-22

**Authors:** Roel May, Yngve Steinheim, Pål Kvaløy, Roald Vang, Frank Hanssen

**Affiliations:** ^1^ Norwegian Institute for Nature Research (NINA) Trondheim Norway; ^2^ SINTEF Information and Communication Technology Trondheim Norway; ^3^ Present address: Rittmestervegen 7 N‐7026 Trondheim Norway; ^4^ Present address: Department of Engineering Cybernetics Norwegian University of Science and Technology N‐7491 Trondheim Norway

**Keywords:** bird monitoring, clutter, detection probability, swerling, target detection, unmanned aerial vehicle

## Abstract

Microwave radar is an important tool for observation of birds in flight and represents a tremendous increase in observation capability in terms of amount of surveillance space that can be covered at relatively low cost. Based on off‐the‐shelf radar hardware, automated radar tracking systems have been developed for monitoring avian movements. However, radar used as an observation instrument in biological research has its limitations that are important to be aware of when analyzing recorded radar data. This article describes a method for exploring the detection capabilities of a dedicated short‐range avian radar system used inside the operational Smøla wind‐power plant. The purpose of the testing described was to find the maximum detection range for various sized birds, while controlling for the effects of flight tortuosity, flight orientation relative to the radar and ground clutter. The method was to use a dedicated test target in form of a remotely controlled unmanned aerial vehicle (UAV) with calibrated radar cross section (RCS), which enabled the design of virtually any test flight pattern within the area of interest. The UAV had a detection probability of 0.5 within a range of 2,340 m from the radar. The detection performance obtained by the RCS‐calibrated test target (−11 dBm^2^, 0.08 m^2^ RCS) was then extrapolated to find the corresponding performance of differently sized birds. Detection range depends on system sensitivity, the environment within which the radar is placed and the spatial distribution of birds. The avian radar under study enables continuous monitoring of bird activity within a maximum range up to 2 km dependent on the size of the birds in question. While small bird species may be detected up to 0.5–1 km, larger species may be detected up to 1.5–2 km distance from the radar.

## 1 INTRODUCTION

Birds link ecosystem processes and communities over long distances making them special from the perspective of ecosystem services, including transport of energy, nutrients, propagules, parasites, and pathogens (Bauer & Hoye, 2014; Whelan, Wenny, & Marquis, 2008). The study of flight behavior, migration phenomena, and responses of birds to man‐made structures such as wind turbines requires ways to observe and document the movement of birds in the area of interest. Traditionally, flight activity and bird migration have been studied using techniques like observations from vantage points, line‐ and point‐transects, and relocation of ringed birds (Sutherland, Newton, & Green, 2004). More recently, these methods have been complemented with individual‐based telemetry methods (Bridge et al., 2011). Although built and optimized for a completely different purpose, both long‐range meteorological radars and dedicated short‐range radars can be used to extract and track bird activity in space and time (Urmy, Warren, & Parrini, 2016; Van Den Broeke, 2013). Such extraction of bird migration from long‐range meteorological radar data has been carried out with success in, for example, the Netherlands (Dokter et al., 2011; Van Gasteren, Holleman, Bouten, Van Loon, & Shamoun‐Baranes, 2008) and the USA (Buler & Dawson, 2014; Gauthreaux & Livingston, 2006). Networks of meteorological radars and associated radar ornithologists have the potential to reveal intercontinental bird migration patterns and phenomena (Chilson, Bridge, Frick, Chapman, & Kelly, 2012; Shamoun‐Baranes et al., 2014). Dedicated short‐range avian radar complements such large‐scale information by enabling surveillance of local key sites to study spatiotemporal movements of (migrating) birds (Beason, Nohara, & Weber, 2013; Dokter, Baptist, Ens, Krijgsveld, & van Loon, 2013; Gerringer, Lima, & DeVault, 2016; McCann & Bell, 2017; Urmy et al., 2016). Automated short‐range avian radar tracking systems (e.g., Accipiter Radar Technologies, Canada; DeTect, USA; Robin Radar Systems, the Netherlands) have been developed commercially to aid in pre‐ and postconstruction studies and monitoring collision risk at airstrips and in wind‐power plants (Cabrera‐Cruz & Villegas‐Patraca, 2016; Coates, Casazza, Halstead, Fleskes, & Laughlin, 2011; Gerringer et al., 2016; Krijgsveld et al., 2011; Plonczkier & Simms, 2012; Skov et al., 2016; Villegas‐Patraca, Cabrera‐Cruz, & Herrera‐Alsina, 2014).

However, as for all methods, radar technology also has its own limitations which are important to be aware of when employing such systems (Beason et al., 2013; Dokter et al., 2013; McCann & Bell, 2017; Schmaljohann, Liechti, Bachler, Steuri, & Bruderer, 2008; Urmy et al., 2016). Dependent on a set of system parameters, such as the wavelength, detection of objects using radar may be affected by environmental conditions (Dokter et al., 2013; Krijgsveld et al., 2005, 2011; Schmaljohann et al., 2008). Given its location, the radar's transmitted energy may be reflected against structures in the landscape, be that topography, vegetation, and/or man‐made structures, creating zones where the radar is totally blind or have reduced detection capability (Beason, Humphrey, Myers, & Avery, 2010; Dokter et al., 2013; Gerringer et al., 2016; Schmaljohann et al., 2008). Wind turbines, for example, have a huge radar cross section with complicated signatures in time and Doppler and represent a particular clutter challenge to any radar that may have them within its field of view. Received signal energy decreases exponentially with distance and causes the targets to fade quickly as they move out in range, which is especially of importance for the detection of smaller objects such as birds. In addition to radar‐inherent limitations, off‐the‐shelf automated avian radar track‐while‐scan systems employ algorithms specifically designed to handle unwanted reflections (clutter) and track birds (i.e., moving targets) over time. These tracking algorithms may potentially further affect detection of a bird in—irregular—flight with regard to aspect and tortuosity (Dokter et al., 2013; McCann & Bell, 2017). Given the radar beam width and the surrounding terrain at a site, detection probability decreases with distance (Krijgsveld et al., 2005) as well as parts of the line‐of‐sight may be obstructed thus limiting the altitudinal coverage (Schmaljohann et al., 2008). However, such tracking systems are often considered to be “black boxes” with proprietary clutter‐reducing and tracking algorithms (Dokter et al., 2013). Contrary to calibrations performed for off‐the‐shelf radars (Urmy et al., 2016), automated radar tracking systems require a calibrated moving target (McCann & Bell, 2017) for assessing detection capabilities.

The aim of this study was to verify the performance of avian radar concerning the detection and tracking of small flying objects, such as birds, within the settings of a wind‐power plant. Given the environment it was placed in, we investigated the limitations of the avian radar in successfully detecting moving targets. Although the performance results will be highly site‐, system‐, and setting‐specific, the methodologies presented enable replication at other sites and will be applicable to similar types of track‐while‐scan radar systems.

## 2 MATERIALS AND METHODS

### 2.1 Study area

**Figure 1:**
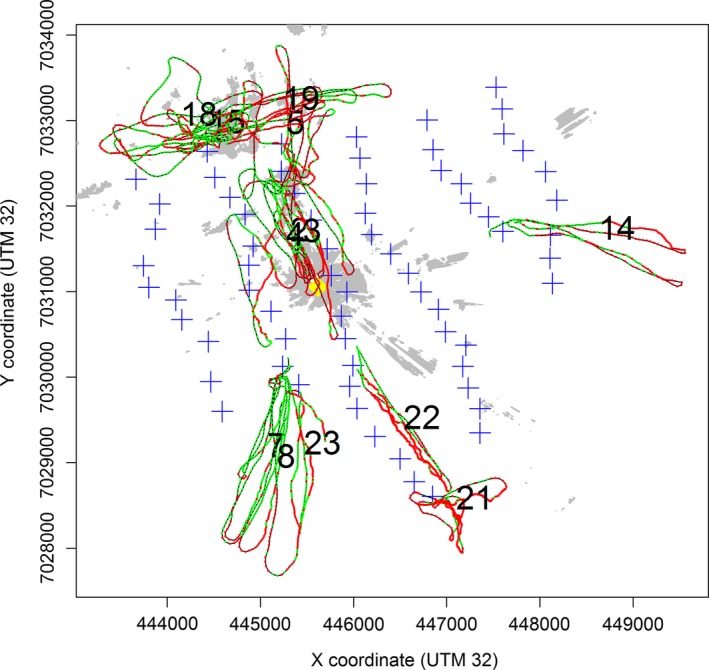
All tracks included in the detection analyses (detections and nondetections are shown in green and red, respectively). The yellow diamond indicates the location of the radar, the blue crosses indicate the wind turbines, and the gray areas represent areas in line‐of‐sight of the radar (i.e., clutter areas). The numbers indicate the track ID (cf. Table 1)

Smøla is an archipelago located off the coast of Møre & Romsdal County, Central Norway (63°24′N, 8°00′E) (Figure 1), and consists of a large main island together with approximately 5,500 smaller islands, islets, and small skerries. The terrain is flat, and the highest peak on the main island is only 69 m. The habitats are characterized by relatively flat open terrain consisting of heath and marsh vegetation, and rocky outcrops, interspersed with minor bogs and lakes. The Smøla wind‐power plant is situated on the northwest side of the main island. It was built in two phases by the Norwegian energy company Statkraft; the first phase consisting of 20 2.1 MW turbines was finished in September 2002, while the second phase with an additional 48 2.3 MW turbines became operational in August 2005. Since 2005, the wind‐power plant has comprised 68 turbines. The wind‐power plant covers an area of 17.83 km^2^, represented by the minimum convex polygon (i.e., envelope) around the outermost turbines including a 200‐m buffer. The wind‐power plant area is accessible through unpaved maintenance roads.

### 2.2 Avian radar description and recordings

The avian radar system was positioned in the middle of the wind‐power plant and operated with an instrumented range of two nautical miles (ca. 3.7 km), which provided an instrumented radar coverage of the complete wind‐power plant area. The MERLIN Avian Radar System model XS2530e (DeTect, Inc.) subjected to the tests is an automated processor of radar data enabling the continuous recording of bird activity 24/7 (Bevanger et al., 2010). The system is based on cost‐effective off‐the‐shelf hardware, using standard “T‐bar” ship radars. The radars in the system are standard S‐ and X‐band radars with nominal frequencies of 3,050 MHz and 9,410 MHz, respectively. The radar antennas are so‐called fan beam antennas, which mean that they have a narrow beam in the horizontal plane and wide beam in the elevation plane. The horizontal and vertical 3 dB one‐way beam widths are 1.9°/30° and 1.0°/20° for the S‐band and X‐band, respectively. They are operated independently from each other. The S‐band radar is used in normal horizontal surveillance mode, while the base of the X‐band antenna is tilted 90° giving a vertical scan pattern which enables height measurements in a narrow sector. The ship radars have antennas which give horizontal polarization of the transmitted electromagnetic wave. As the X‐band antenna in this case is mechanically tilted 90°, the X‐band polarization will be vertical. The detection tests are solely based on the horizontal S‐band radar. Although the radar hardware is not designed specifically to capture small flying objects such as birds, the developed data extractor is especially designed to do extract small flying objects from the radar signals. The target data extractor has two main functions: detection and tracking. The detection process establishes the automatic detection thresholds on a combined background of clutter and system noise and, for each antenna scan, detects any signal level above this threshold as a target. The tracker takes the detections as input and, based on the movement characteristics of birds, performs scan to scan processing to identify and combine successive detections of the same target. Detections from several antenna scans found to be from the same moving target are stored together as a “target track” in the target database. The entire system is mounted on a trailer and can thus be moved to any desired location for data collection.

### 2.3 Detection test flights using UAV

A remotely controlled unmanned aerial vehicle (UAV) was used as a test target to investigate the performance of the radar system in its actual operating environment. The UAV was equipped with a video camera and video link to the controlling pilot on the ground. This expands the controlling range up to ranges in excess of 2 km which makes it possible to design and perform virtually any test flight pattern within the wind park and the radar coverage. The UAV fuselage is made from balsa tree with ribs of plywood, covered with a plastic foil. Apart from the flaps, the wing consists only of plywood ribs with a plastic foil cover and has a wingspan of 2.1 m. The radar returns from these wooden structures, and plastic foil was anticipated to be minor and that the dominant radar scatterers were the different mounted metallic parts: motor, battery pack, and remote control receiver, GPS‐unit, and a 500 mW video transmitter (2.4 GHz) mounted on top of the wing.

The detection tests were executed on 17 August 2009 to 20 August 2009. Weather conditions at that time were characterized by calm and clear skies without any precipitation. At the time of testing, the wind turbines were not operational. The performance test was therefore not affected by adverse conditions rendering dynamic sources of clutter, precipitation, and/or operational turbines, which reduce detection. Take‐off of the UAV was carried out from the unpaved roads near different turbine bases (Figure 1). Flight lags for detection over range (*N* = 7) were done at circa hub height (70 m) away from and toward the radar. Flight lags over clutter areas (*N* = 6) passed over specific areas repeatedly. The radar and GPS data are provided as supplementary information (Data S2).

### 2.4 Correlating radar tracks and GPS tracks

Because the time stamps of the UAV's GPS and the Merlin avian radar system were not synchronized, we had to estimate the offset in time between GPS flight tracks and radar tracks to be able to correlate the data sets properly. First, we gave each GPS track (one for each test) and associated radar tracks a unique test‐ID, thereby clustering both roughly. Thereafter, we estimated the offset in seconds *T*_*i*_ *℮* [0,120] between GPS and radar for each test‐ID *i* by maximizing the log‐likelihood of the following linear model:

(1) $$D_{i,t}^{2}=\beta_{0}+\beta_{1}\times \Delta R_{i,t}^{2}+\beta_{2}\times \Delta B_{i,t}^{2}$$

with *D*_*i,t*_ as the distance, *ΔR*_*i,t*_ as the difference in range from the radar (*R*_radar_ *– R*_gps_), and *ΔB*_*i,t*_ as the distance effect due to the difference in bearing α from the radar (sin(α*/2*)·(*R*_radar_ *+ R*_gps_)) between each position pair (GPS position and time‐adjusted radar position) *i,t*. After having found the most likely offset (Table 1), we connected the radar‐detected positions to the GPS positions by timestamp. Thereafter, we derived nondetections by interpolating consecutive detections with an average time difference of 2.7 s (i.e., 22.5 radar scans per minute). The S‐band pulse repetition frequency (PRF) used is 1,900 Hz with a pulse width of 70 ns, corresponding to 10.5 m in distance (range). The best‐case angular resolution of the system is approximately equal to the 3 dB beam width, and the best‐case range resolution is equal to the pulse width, giving a theoretical resolution cell of 10.5 m × 1.9°. The actual resolution experienced in a practical system is usually coarser, dependent on the actual signal processing in the receiver chain. R code is provided as supplementary information (Data S1).

**Table 1:** Overview over the flight tracks of the unmanned aerial vehicle and the estimated offset in seconds. The last column indicates in which test the track data was used: detection over range (DR) and over clutter areas (DC)

Track ID	Offset (sec)	Test type	Average range (m)	Proportion over clutter areas
2	43.578	DR	621	0.41
3	46.169	DC	659	0.38
4	45.836	DC	825	0.13
5	46.169	DC	1,837	0.08
7	47.124	DR	2,062	0.00
8	47.057	DR	2,169	0.00
14	46.228	DR	3,073	0.00
15	45.836	DC	2,198	0.18
18	51.798	DC	2,434	0.10
19	54.042	DC	2,178	0.18
21	59.089	DR	2,999	0.00
22	57.007	DR	1,896	0.00
23	58.302	DR	1,965	0.00

### 2.5 Detection theory and in practice

The ability for an object to reflect the signal energy back to the radar is called its radar cross section (RCS) and is measured in square meters (Barton & Leonov, 1997). The main problem in detecting birds with a radar system is the small RCS. The signal level in the radar receiver from any object within its coverage is proportional to the objects RCS. The literature reports for large birds (e.g., a swan or an eagle) an average RCS of −20 dBm^2^, or 0.01 m^2^, measured at a frequency of 10 GHz (Eastwood, 1976; Skolnik, 2001; Vaughn, 1985). In comparison, a small aircraft may be in the order of 1–3 m^2^. However, the measured RCS depends on several factors, such as the size and shape of the bird, the frequency, and the aspect angle. The RCS of complex targets will therefore fluctuate in time. In an attempt to capture target RCS fluctuation effects in a mathematical model that could be easily used in detection studies, Peter Swerling developed statistical representations of RCS fluctuations that are commonly referred to as the Swerling target models. The theoretical probability of detection (*P*_d_) will depend on the type of RCS fluctuation model used and is a function of the signal‐to‐noise ratio (SNR). Theoretically, the SNR in the receiver for a target with RCS σ is obtained by the radar equation (Skolnik, 2001). The radar equation represents the physical dependence of radar properties (e.g., wavelength, antenna gain, transmit power) and inherent loss factors (e.g., atmospheric, attenuation, and fluctuation losses) with RCS and range (~*R* ^−4^) (Larkin & Diehl, 2012).

Which target model to use depends on the type of target and properties of the radar waveform. The Swerling‐0 model describes the relationship for “steady” (nonfluctuating) targets. The Swerling‐1 model describes a target whose RCS is constant throughout the illumination time, that is, antenna scan, but varies independently from scan to scan. One of the standard assumptions is that the Swerling‐1 model is associated with complex targets such as aircraft and ships with many independent scattering points. In practice, detection measurements indicate that, indeed, the Swerling‐1 models provide a good representation of complex targets (Klein & Sadovnik, 2006; Swerling, 1954). The *P*_d_ for a fluctuating target (i.e., Swerling‐1 model) follows the equation:

(2) $$P_{d}=e^{\frac{\ln\;(P_{fa})}{\mathrm{SNR}+1}}$$

with *P*_fa_ as the probability of false alarms (typically ~10^−6^).

Although it is unclear from the literature whether birds as radar targets follow the latter “fluctuation model,” we expect that the UAV can be approximated as a Swerling‐1 target. In the radar equation, this gives rise to a growing fluctuation loss when *P*_d_ is above circa 0.3. For *P*_d_ = 0.5 the fluctuation loss lies around 2 dB compared to a target that does not fluctuate (Barton, 2004). If birds are to be expected to be a more “steady” target, then they thus have detection advantage over the UAV, although they may have the same average RCS.

However, to be of use in practical radar problems we need to attempt to relate this statistical model to actual targets detected by the Merlin avian radar system. The Swerling‐1 equation follows a (continuous) chi‐squared (or gamma) distribution (Swerling, 1954). When the detections (and nondetections) are seen as independent Bernoulli trials, this process can be approximated with a binomial distribution. The output data of the avian radar system have gone through several processing algorithms. Because of the heterogeneous clutter surrounding the radar and the algorithms in the automated processing, the relationship between *P*_d_ and range as part of the radar equation may not be as simple (~$e^{-R^{4}}$). The same applies to the relationship between *P*_d_ and *P*_fa_. Given the nature of our data (independent detections and nondetections), we chose to follow a binomial approach, and for simplicity chose to model detection over range on a linear scale (cf. Dokter et al., 2013).

### 2.6 Statistical modeling

R code for all analyses is provided as supplementary information (Data S1.2). Prior to statistical analyses, we assessed data quality and excluded extreme values from the analyses based on visual inspection of the distribution of and cumulative standard deviation in detection rates for altitude above ground level and tortuosity (Gerringer et al., 2016). Low‐flying objects may be obscured by terrain formations limiting detection (during take‐off and landing of the UAV). Sharp and unpredictable in‐flight turns and doubling‐back are generally difficult for tracking algorithms (Schell, Linder, & Zeidler, 2004); however, any inability in doing so was not the focus of this study. Tortuosity, defined as the normalized cosine of the turning angle of consecutive positions, (*S*_i_) was calculated for each position *i* as follows: *S*_i_ = 1 – ((cos Δ*H*_i_ + 1)/2), with Δ*H*_i_ as the change in heading between two consecutive positions. We used segmented regression to find the optimal partitioning at varying thresholds. Segmented regression aims to find the threshold (i.e., minimum altitude or maximum tortuosity) that renders the minimum sum of squared residuals. As detection may also be affected by the orientation of a track toward the radar (cf. Schmaljohann et al., 2008), we included a grouping covariate to differentiate between track segments along and across (|cos(α)| > cos(45°)) the radar beam.

Thereafter, detection over range (i.e., distance to radar) and over clutter areas (i.e., binary variable indicating whether the position was inside (=1) or outside (=0) a clutter area) were assessed for each track cluster separately (Table 1, Figure 1). Ground clutter was modeled in GIS delineated by areas in line‐of‐sight to the radar (Figure 1). The within‐track detection probability analyses were modeled using mixed‐effects generalized linear models with a binomial distribution. For this, we used the glmer function in the lme4 library of statistical program R 3.1.2 (R Core Team 2014). Here, successful detections (versus nondetections) were related to different covariates while controlling for track orientation and tortuosity and a random grouping over tracks (i.e., intertrack dependence).

Because the MERLIN software is able to maintain a track, all else being equal, when every other detection is lost, we set the minimum detection probability level to 0.5, which then defined our “maximum detection range” in the detection over range analyses. From this, we could derive the expected detection over range and clutter areas the MERLIN avian radar system could handle successfully. For reasons of comparison, we also estimated detection range using a traditional approach and estimated the “blip/scan”‐ratio (Skolnik, 2001). Here, we estimated the *P*_d_ by counting the number of detections in 25‐m range bins and divided them by the total number of detection opportunities for the target within each bin. From this plotted graph, we visually assessed the detection range for *P*_d_ = 0.5.

### 2.7 RCS measurement of the UAV for extrapolation of detection range to bird targets

**Figure 2:**
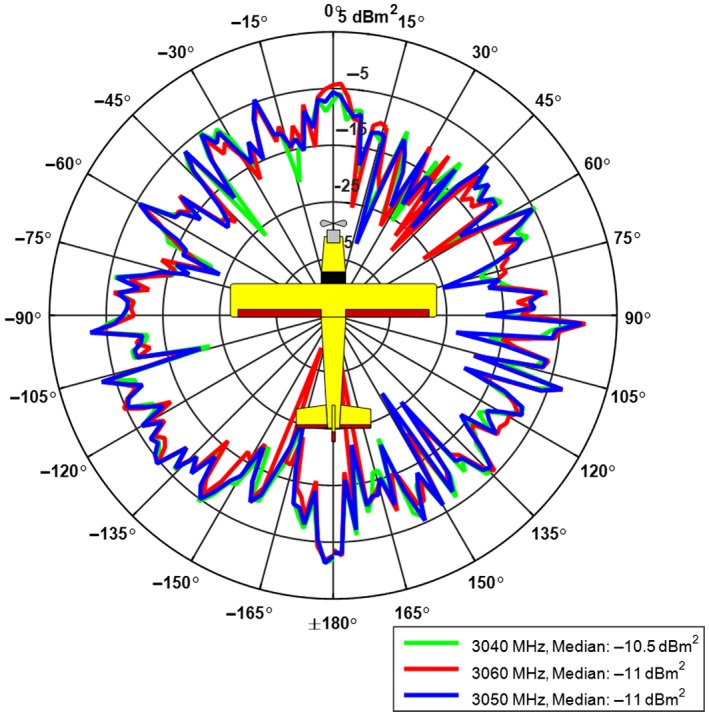
Unmanned aerial vehicle S‐band radar cross section (RCS) at 0° elevation

To be able to compare the performance of the avian radar system, using the test target, against the actual performance with real bird targets, the radar cross section (RCS_d_) of the test target must be known. The challenge of using a UAV as test target is that the RCS of the UAV generally is unknown and much more difficult to control than the RCS of simple targets as, for example, a conducting sphere. It is expected to vary substantially as a function of the radar wavelength and the aspect angle. This is due to the irregular shape and positioning of the different scatterers on the UAV. An important prerequisite when using the UAV as a test target is therefore that its RCS is measured and verified for the relevant aspect angles and radar wavelengths. RCS measurement of the UAV has been performed in an anechoic chamber at the NTNU/SINTEF antenna laboratory facilities at Gløshaugen, Trondheim (Appendix 1). The median RCS of the UAV at 3,050 MHz (S‐band wavelength) over 360° was −11.0 dBm^2^, that is, 0.08 m^2^, and the 75 percentile was measured at −7.5 dBm^2^, 0.18 m^2^, and the 25 percentile at −14.6 dBm^2^, 0.03 m^2^. The UAV had distinct lobes head‐on, tail‐on, and from the sides. These lobes were however not much larger than lobes in other directions, indicating that it is not the balsa and plywood wings and fuselage itself that caused the biggest returns, but rather the wiring and the different parts mounted both inside and outside the UAV (Figure 2).

Finally, the modeled and binned detection ranges (*R*_d_) derived from the UAV were extrapolated to bird targets with RCS_i_ using the information from the RCS measurements on the UAV (RCS_d_). This was performed using the following formula (Knott, Shaeffer, & Tuley, 2004):

(3) $$R_{i}={\left(\frac{10^{{\mathrm{RCS}}_{i}/10}}{10^{{\mathrm{RCS}}_{d}/10}}\right)}^{1/4}\times R_{d}$$

where the RCS (in dBm^2^) of the various species was estimated assuming a spherical water body (Moon, 2002):

(4) $${\text{RCS}}_{i}=10\times \log_{10}\left({{\left(\frac{W_{i}\times 0.65\times 3}{1000\times 1000\times 4\pi }\right)}^{2/3}}^{}\times \pi \times 0.56\right)$$

with *W*_*i*_ as the weight of the bird (in grams). Weights for the different species were obtained from the FLIGHT software (Pennycuick, 2008).

## 3 RESULTS

The segmented regression rendered a lower altitude threshold of 27 m above ground level and upper tortuosity threshold of 0.33 (indicating a turning angle of 70°), above/below which the standard deviation stabilized. For further analyses, we excluded altitudes below 27 m and tortuosity values above 0.33 from the data (signifying 4.1% of the data).

**Figure 3:**
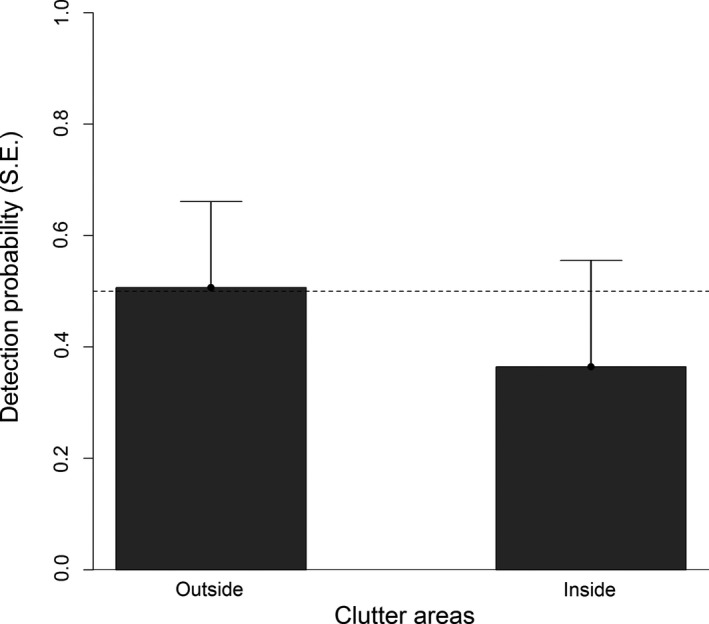
Detection over clutter areas. Modeled relationship and detection limit (at *P*_d_ = 0.5) for six flight tests using a binomial mixed‐effects model

**Figure 4:**
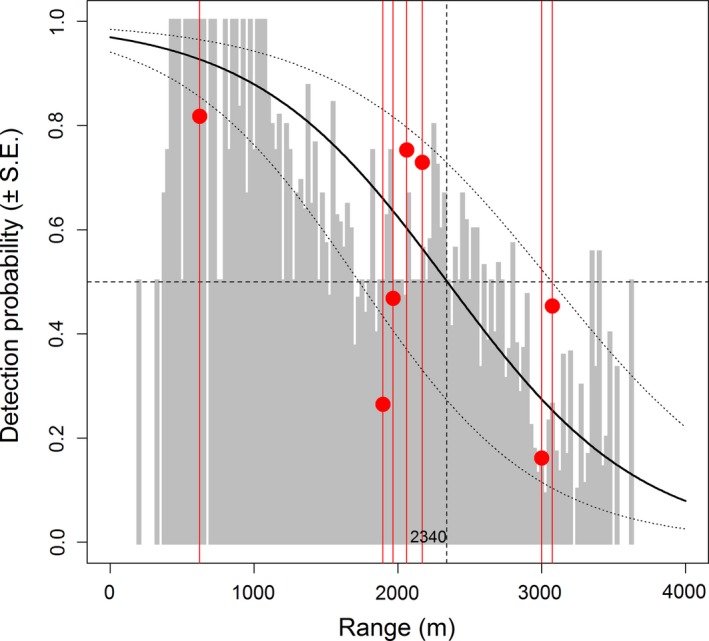
Detection over range. Proportion of detections by the total number of detections are given in gray, clustered in 25‐m bins for seven range‐detection tracks. Predicted relationship (±*SE*) and detection range (at *P*_d_ = 0.5) using a binomial mixed‐effects model. The red lines indicate the average range for the different tracks. Red dots indicate the overall track detection probability

The detection tests over clutter areas showed a 28% (16–41% CI) reduction in the detection probability within land clutter areas (*z *=* *−4.232, *p *<* *.001) from 0.51 (0.35–0.66 CI) to 0.36 (0.21–0.56 CI) while controlling for potential effects over range (*z *=* *2.756, *p *=* *.006), track orientation (*z *=* *−1.969, *p *=* *.05), and tortuosity (*z *=* *−0.408, *p *=* *.683) (Figure 3). To further test for range of detection, we only included positions that were positioned outside clutter areas. The detection range, given a threshold detection probability (*P*_d_) of 0.5, was 2,340 m (1,720–3,060 m CI) from the modeled data (*z *=* *−11.951, *p *=* *.037), while controlling for track orientation (*z *=* *2.088, *p *<* *.001) and tortuosity (*z *=* *−3.650, *p *<* *.001) (Figure 4).

**Figure 5:**
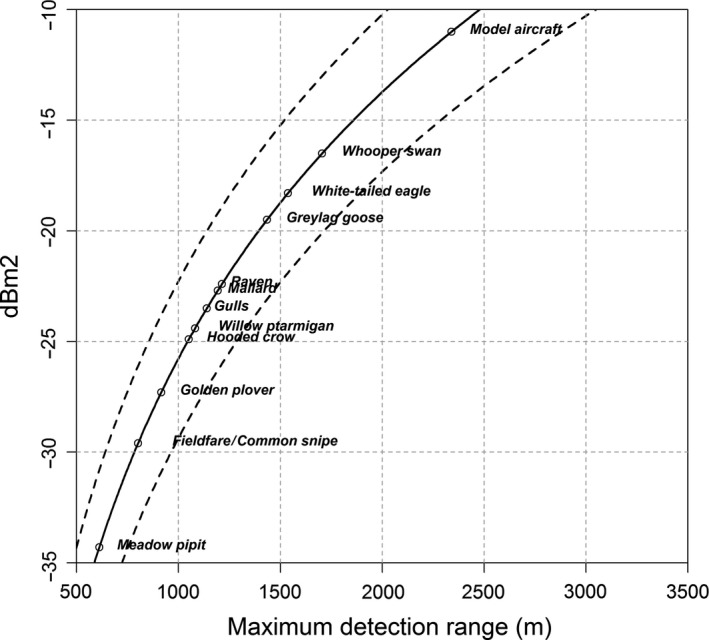
Extrapolation of the detection range for different bird species with different radar cross section (RCS) (Table 2), based on a modeled detection range of 2,340 m (see Figure 4)

Based on the measured RCS of the UAV and estimated average detection range (2,340 m; Figure 4), we extrapolated the expected detection range to a range of bird species with different sizes (Figure 5). Table 2 compares modeled detection ranges at a *P*_d_ of 0.5 with observed ranges of groundtruthed radar tracks of various bird species. Although the groundtruthed birds did not represent a representative subset of the geographic distribution of birds in the area, it does give an indication of the range of possible detections.

**Table 2:** Groundtruthed birds which were detected by the Merlin avian radar system, and the range (m) at which they were tracked. The lower and upper ranges represent the extremes including both uncertainty around the modeled detection range (2,340 m, 1,720–3,060 m CI) and quartiles of the radar cross section (RCS) calibration for each species (group). The observed mean, *SE*, and range values were derived from *N* groundtruthed birds observed in the field and tracked by the avian radar simultaneously

Detection range	Passerines^a^	Waders^b^	Hooded crow (*Corvus cornix*)	Gulls	Mallard (*Anas platyrhynchos*)	Raven (*Corvus corax*)	Greylag goose (*Anser anser*)	White‐tailed eagle (*Haliaeetus albicilla*)
RCS (dBm^2^)	−32.0	−28.5	−24.9	−23.5	−22.7	−22.4	−19.5	−18.3
Modeled	707	859	1,051	1,140	1,193	1,214	1,435	1,537
Lower range	578	703	859	932	975	992	1,173	1,257
Upper range	870	1,057	1,293	1,402	1,468	1,494	1,765	1,891
Mean	959	2,058	1,013	1,653	1,434	1,043	1,932	1,497
*SE*	53	179	49	48	202	63	114	22
Minimum	135	371	214	346	418	292	504	249
Maximum	2,866	4,377	2,869	3,564	3,103	3,446	3,722	3,643
*N*	76	33	74	220	11	58	38	725

a Represented by the Fieldfare (*Turdus pilaris*) and Meadow pipit (*Anthus pratensis*).

b Represented by the Common snipe (*Gallinago gallinago*) and Golden plover (*Pluvialis apricaria*).

## 4 DISCUSSION

The modeled species‐specific detection ranges signify the minimum limit beyond which the avian radar tracking system is expected to lose track of a given bird (cf. Gerringer et al., 2016). In other words, within this limit the radar system may be expected to maintain a track up to the moment when every other plot is undetected (*P*_d_ = 0.5). The detection range is however dependent on a number of external factors limiting detection such as atmospheric conditions, ground clutter, altitudinal coverage, and inference from large objects or other radar systems nearby (i.e., shadowing) (Beason et al., 2013). The analyses in this study controlled for potential sources of reduced environmental detectability due to altitude, clutter, and track orientation. In this study, we also assumed that a bird's RCS equals its water content not taking into account shape and form of the bird. Birds may be better approximated by a prolate spheroid with length‐to‐width ratios of 2–3 than by the often used equivalent weight water sphere (Vaughn, 1985). Assuming a more or less crucifix bird shape may thereby, relative to its size, affect detection probability with respect to movement ventral and lateral to the radar beam (McCann & Bell, 2017; Schmaljohann et al., 2008). Birds, however, do not resemble a static crucifix shape. Various bird species deploy a range of different flight modes, including soaring, flapping and intermittent bounding and undulating flight (Norberg, 1990; Pennycuick, 2008). To which extent the flight mode affects radar detectability is however uncertain. In addition to aspect and flight mode, detection and tracking are likely affected by flight tortuosity. Irregular flight of small maneuvering targets (acceleration, sharp turns, crossing, and flocking) complicates successful tracking of birds in a cluttered environment (Beason et al., 2013; Schell et al., 2004). Here, we also have to distinguish between the sensitivity of the radar hardware to sense a (static) reflected bird echo, and the subsequent clutter suppressing algorithms and tracking software to detect this echo from background clutter and be able to track bird targets. Although the first two can be seen as being limitations inherent to the system employed (as regarding accuracy, sensitivity, and resolution), the potential influence of the latter may be hard to assess. Most currently commercially available avian radar tracking systems process radar echoes employing “black box” algorithms to identify and track birds in space and time (Beason et al., 2013; Dokter et al., 2013; Gerringer et al., 2016). In this study, we controlled for any potential effects of tortuosity in the detection probability, both by excluding extreme values (cf. Urmy et al., 2016) and by including random effects in our models. The detection ranges estimated in this study were similar, albeit somewhat more conservative, compared to other validation studies (Beason et al., 2010; Dokter et al., 2013; Gerringer et al., 2016). Comparing to the groundtruthed birds clearly shows that birds in specific situations can be detected by the radar up to two nautical miles, the maximum instrumented range at which the radar was set. Still, this does not equate to the detection range at a set detection probability (of e.g., 0.5). Discrepancies between this study and visual observations may in part be explained by the sensitivity of the systems employed, the environment within these were placed and the spatial distribution of birds within the areas. In addition, the larger observed distances for greylag goose, mallard, and gulls may in part also be explained by social behavior when they are flying in flocks. The smallest bird species, differing most in size from the UAV, seemed to result in an underestimation of their detection range. This would merit further studies. Still, from our study and other validation studies (Beason et al., 2010; Dokter et al., 2013; Gerringer et al., 2016) it becomes clear that single birds can in general be detected within a circular area with a maximum range up to 2 km (ca. 1 nautical mile) from the radar, representing a surface area of ca. 12.5 km^2^. However, detection range depends on the size of the bird, with smaller species (e.g., fieldfare, meadow pipit) being detected up to 0.5–1 km and larger species (e.g., whooper swan, white‐tailed eagle) up to 1.5–2 km from the radar. Thus, to enable continuous monitoring of bird activity within a relatively large area of interest, radar may still be the best available technology at present (Chilson et al., 2012; Desholm, Fox, Beasley, & Kahlert, 2006). This performance test was executed within the settings of a wind‐power plant, including the static clutter from present structures (wind turbines, buildings, and power line) as well as the surrounding terrain. As the test was executed during calm and dry circumstances, thereby minimizing any dynamic clutter from, for example, operational turbines and precipitation, these ranges should be seen as “optimal” ranges within an application‐relevant environment. Although the performance results will be highly site‐, system‐, and setting‐specific, the methodologies presented enable replication at other sites and will be applicable for similar types of radar systems and situations.

## AUTHOR CONTRIBUTION

All authors contributed to the design of the test and acquisition of the data in the field. YS contributed to input concerning radar theory. PK and RV supported in the database management of radar and GPS data. FH calculated the line‐of‐sight clutter mapping. RM executed the statistical analyses and interpretation of the results. All authors contributed to the drafting of the article, and gave final approval for publication.

## CONFLICT OF INTEREST

None declared.

## DATA ACCESSIBILITY

Further details on the RCS measurement of the UAV are given in Appendix 1. R scripts used to prepare the data and test the detection range of the MERLIN Avian Radar System are uploaded as Supplemental Information. An Excel file with the verification data used in this study is uploaded as supplemental information to the article as well.
