# Supplementary material for: Performance test and verification of an off‐the‐shelf automated avian radar tracking system
Source: Ecol Evol. 2017 Jun 22;7(15):5930–8. doi: 10.1002/ece3.3162 (PMC5552925; doi:10.1002/ece3.3162)
Supplement: Supplementary file 4 [file ECE3-7-5930-s001.pdf]

MEMO

SINTEF ICT

Address: NO-7465 Trondheim, NORWAY
Location: O S Bragstads plass 2C, NO-7034 Trondheim
Telephone: +47 73 59 30 00
Fax: +47 73 59 10 39

Enterprise No.: NO 948 007 029 MVA

MEMO CONCERNS

Radar Cross Section measurement of model aircraft used as avian radar test target

DISTRIBUTION	FOR YOUR ATTENTION	COMMENTS ARE INVITED	FOR YOUR INFORMATION	AS AGREED
Kjetil Bevanger/NINA	X			
Roel May/NINA	X			

FILE CODE	CLASSIFICATION
90F259/N01-10/YS	Confidential

ELECTRONIC FILE CODE

90F290_model_aircraft_rcs_v2.0.docx

PROJECT NO.	DATE	PERSON RESPONSIBLE / AUTHOR	NUMBER OF PAGES
90F259	2016-09-22	Yngve Steinheim	65

This memo contains project information and preliminary results as a basis for final report(s).
SINTEF accepts no responsibility of this memo and no part of it may be copied.

Table of Contents

1 Background **3**

2 Radar Cross Section (RCS) **4**
2.1 Definition 4
2.2 Far field requirement 5

3 Method **5**
3.1 Selection of approach 5
3.2 Measurement set-up 7
3.3 Estimation of RCS 9
3.4 Calibration 10

4 Measurements **11**
4.1 S-band 12
4.1.1 S-band calibration 12
4.1.2 S-band RCS measurements 13
4.2 X-band 18
4.2.1 Horizontal polarisation 18
4.2.1.1 X-band horizontal polarisation calibration 18
4.2.1.2 X-band horizontal polarisation RCS measurements 20
4.2.2 Vertical polarisation 25
4.2.2.1 X-band vertical polarisation calibration 25
4.2.2.2 X-band vertical polarisation RCS measurements 25

5 Summary **30**

6 References **31**

7 Document revision history **31**

Appendix A Model aircraft configuration **32**

Appendix B Equipment list **36**

Appendix C Link budget **38**

Appendix D S-band RCS, horizontal polarisation **39**

Appendix E X-band RCS, horizontal polarisation **48**

Appendix F X-band RCS, vertical polarisation **57**

1 Background

The Norwegian Institute for Nature Research (NINA) has installed an avian radar system in the Statkraft windpark at Smøla to detect and monitor bird flight patterns in and around the wind park area. The radar study of bird movements is a sub-task in the project “Pre- and post-construction studies of conflicts between birds and wind turbines in costal Norway” (“Bird-Wind”). The radar is positioned in the middle of the wind park and normally operates with an instrumented range of about 3 km, which provides instrumented radar coverage of the complete wind park area. A remotely controlled model aircraft has been used as a test target to investigate into the performance of this radar system in its actual operating environment. To enable long-range radar testing NINA has equipped the aircraft a video camera and a video link to the controlling pilot on the ground. This expands the controlling range up ranges in excess of 2 km which makes it possible to design and perform virtually any test flight pattern within the wind park and the radar coverage.

To be able to compare the performance of the avian radar system, using the test target, against the actual performance with real targets, which in this case means large birds e.g. white tailed sea eagle, the Radar Cross Section (RCS) of the test target must be known. The first radar trials at Smøla were performed using a small conducting sphere as the test target. To move the test target around within the radar coverage, the sphere was towed behind the model aircraft. The advantage of using a simple shape as a sphere as the test target is that its RCS is accurately known for any given radius and radar wavelength. This makes it possible to test the radar with different size test targets simply by using different size spheres. Unfortunately the actual radar resolution, mainly limited by the extraction algorithms in the radar processor, was not narrow enough to allow for proper resolution with the towing aircraft at practical line lengths. The method requires that the echo of the sphere can be distinctly separated from any other echoes. In addition; towing a sphere behind a model aircraft poses a real challenge to the remote-controlling pilot on the ground, and places severe restrictions on the freedom to manoeuvre and thus limiting the flight patterns possible to perform. Therefore this method had to be abandoned, and instead the model aircraft itself has been used as the test target.

The drawback of using the model aircraft itself as the test target is that the RCS of the aircraft generally is unknown and much more difficult to control than the RCS of a conducting sphere. It is expected to vary substantially as a function of the radar wavelength and the aspect angle. This is due to the irregular shape and positioning of the different scatterers on the aircraft. An important prerequisite when using the model aircraft as an test target is therefore that its RCS is measured and verified for the relevant aspect angles and radar wavelengths.

The purpose of the work described in this memo has been to investigate and map the RCS of the model aircraft used as the test target for testing of the NINA avian radar at Smøla. The measurements have been performed in an anechoic chamber at the NTNU/SINTEF antenna lab facilities at Gløshaugen, Trondheim. This memo presents the results and also describes and discusses the method used.

2 Radar Cross Section (RCS)

2.1 Definition

The power received from a target with RCS, σ, at distance R from the radar is given by:

$$P_r = \frac{P_t G_t G_r \lambda^2 \sigma F^4}{(4\pi)^3 R^4 L}$$

Eq. 1

Here P_t is the transmitter power, G_t and G_r is the antenna transmit and receive gain respectively, λ is the radar wavelength, F^4 is the pattern propagation factor, which accounts for multipath interference in the transmit and receive path of the radar wave, and L is a loss factor which accounts for all signal loss up to the point in the receiver where the received power is to be measured. This is sometimes referred to as the "radar transmission equation" [4]. If a minimum detectable signal, S_{min}, is defined, and P_r is replaced by this minimum power level, the equation can be solved with respect to range, R, to find the maximum detection range for a given size target:

$$R_{\max} = \sqrt[4]{\frac{P_t G_t G_r \lambda^2 \sigma F^4}{(4\pi)^3 S_{\min} L}}$$

Eq. 2

This expression is the fundamental form of the "radar range equation" [4].

Eq. 1 and Eq. 2, states that the received power from a target, and thereby the maximum detection range of a target, is dependent of its RCS, i.e. the ability of the target to reflect radar energy back to the receiving antenna. A formal definition of RCS can be found in [2]:

..4π times the ratio of the power per unit solid angle scattered in a specified direction to the power per unit area in a plane wave incident on the scatterer from a specified direction. More precisely, it is the limit of that ratio as the distance from the scatterer to the point where the scattered power is measured approaches infinity.

This can be expressed [3]:

$$\sigma = \lim_{R\to\infty} 4\pi R^2 \frac{\left|\boldsymbol{E}^{scat}\right|^2}{\left|\boldsymbol{E}^{inc}\right|^2}$$

Eq. 3

Where $\mathbf{E}^{scat}$ is the scattered field strength in a specified direction and at a distance R from the target and $\mathbf{E}^{inc}$ is the incident field strength at the target.
Although the RCS of targets are measured on test ranges at distances considerably less than infinity, the distances actually used, typically a few tens of meters up to a few thousand meters, are usually great enough to be close enough to "infinity".

A physical interpretation of RCS, for sufficiently large targets, is that it is the projected area of a conducting sphere which would produce the same signal as the target, if it was placed in the same position relative to the radar [2].

2.2 Far field requirement

The definition of RCS in Eq. 3 above, requires that the target is illuminated with a plane wave. In most practical measurement situations, the wave front is spherical due to the finite distance between the radar and the object. It is therefore of interest to find "how spherical" the incident wave front may be and still be an acceptable approximation to a plane wave.

If the radar is considered a point source, and the maximum width of the target defines an aperture facing the radar, is a general rule of thumb that the phase difference over this aperture span must be no larger than 22.5° (π/8) for the wave front to be a reasonable approximation to a plane wave at the object. This places a restriction on the minimum distance between the radar and the target, which RCS is to be measured, given by:

$$R \geq 2 \cdot \frac{D^2}{\lambda}$$

Eq. 4

Here D is the maximum target width and λ is the wavelength.

3 Method

3.1 Selection of approach

The RCS measurements had to be performed at the two frequencies used by the NINA avian radar system at Smøla. The radars in the system are standard S- and X-band ship radars with nominal frequencies of 3050 MHz and 9410 MHz respectively. They are operated independently from each other. The S-band radar is used in normal horizontal surveillance mode, while the base of the X-band antenna is tilted 90° giving a vertical scan pattern which enables height measurements in a narrow sector. The ship radars have antennas which gives horizontal polarisation of the transmitted electromagnetic wave. But since the X-band antenna in this case is mechanically tilted 90°, the X-band polarisation will be vertical.

The maximum extension, or target width, of the model aircraft used as test target, is the wing span, approximately 2.1 m. To comply with the far field requirement in Eq. 4 above, this target must be placed at least 89.7 m away from the radar in S-band and at least 276.7 m away in X-band. In practice, this could only be achieved at an outdoor test range. Therefore a first attempt was made to set up the RCS measurements on the outdoor antenna lab facilities on the roof of the building which houses the SINTEF ICT and the NTNU faculty of Information Technology, Mathematics and Electronics in Trondheim. Even if the measurement range at this site would be considerably shorter than the required far field distances in both S- and X-band.
However, with the measurement techniques and equipment that was available, the interference from in-band external sources, presumably the most powerful ones being ship-radars in the Trondheim harbour area, was too severe to allow for measurement of a small target RCS at this location.

Instead the RCS measurements had to be performed in an anechoic chamber inside in the SINTEF/NTNU antenna lab facilities. In this room the distance from the antenna mount to the measurement tower where the target can be placed, is only about 6-7 m, and consequently the distance between the radar antenna and the test object was well within the far field limit for the maximum extent of the target. But the model aircraft is a relatively complex shape consisting of several distinct parts which will scatter independently at high frequencies. A picture of the interior of the front part model aircraft is in Figure 1 below.

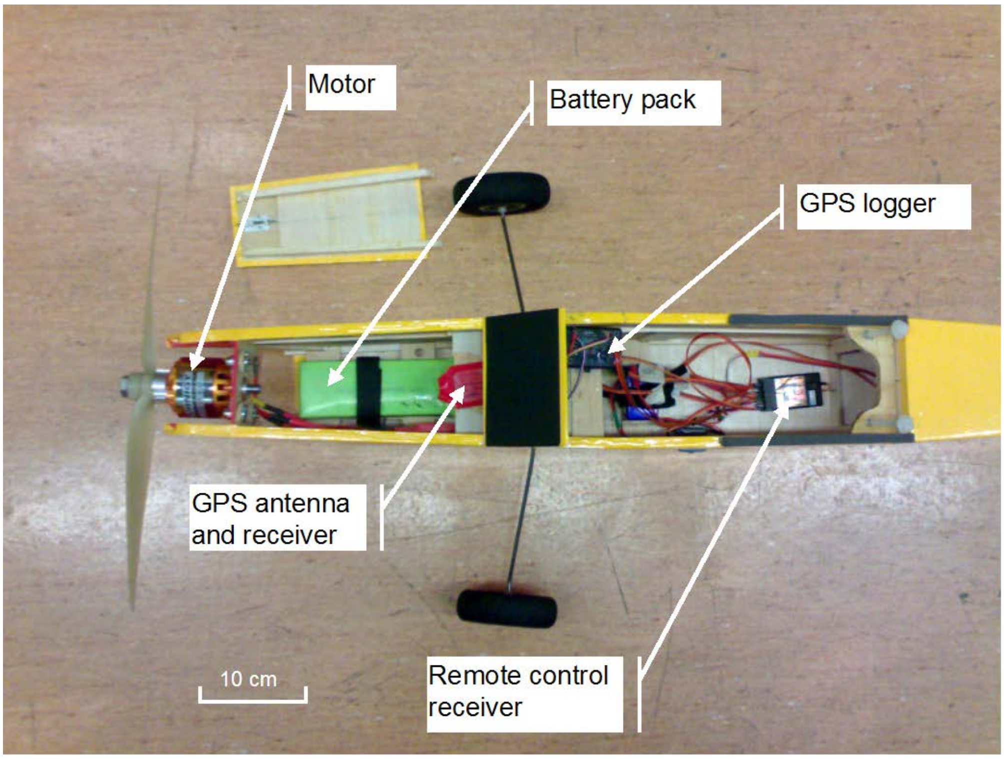

Figure 1 Model aircraft interior

A video camera is mounted on the top of the wing.

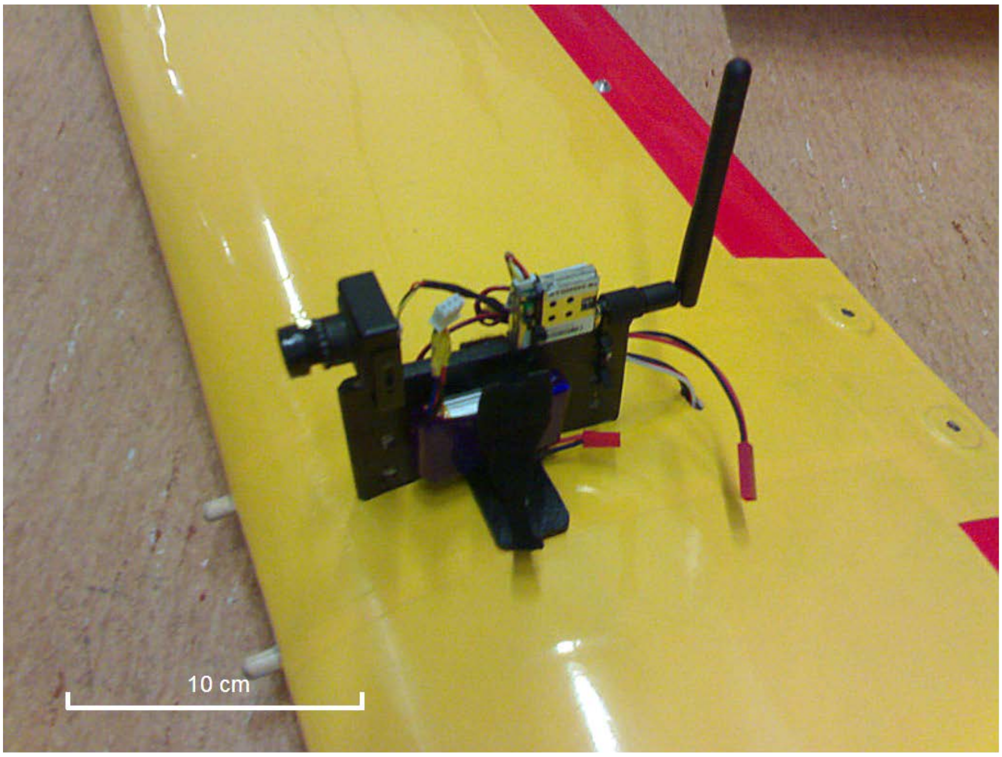

Figure 2 Video camera on top of wing

More pictures of the aircraft and its configuration are in Appendix A. The aircraft fuselage is made from balsa three with ribs of plywood, covered with a plastic foil. Apart from the flaps, the wing consists only of plywood ribs with a plastic foil cover. It is anticipated that the radar return

from these wooden structures and the plastic foil is only minor, and that the dominant S- and X-band scatterers is the different mounted metallic parts. All these structures are small enough to be well within the far-field requirement in both radar bands. But they are interconnected with copper wires which distribute power and control signals inside the aircraft. These wires have different lengths and thicknesses and complicate the modelling of the scattering characteristics of the aircraft. However, apart from the wires connecting the servos in the wings and in the tail and the remote control antenna at the underside of the fuselage, most of the cables are relatively short and located near the cockpit area. Therefore the assumption is still made that the main reflecting structures of the aircraft will be in the far-field and scatter radar energy more or less independently of each other. If this is true, the amplitude and the position of the peaks and nulls in each of the individual scattering patterns is less sensitive to the measurement range, and there will only be a slight shift of the lobes and nulls in the composite pattern compared to the true far-field pattern [3].
An other aspect that supports the validity of the proposed method is that the intention of this RCS measurement effort is not to map the RCS pattern of the model aircraft with a high degree of accuracy, but rather to find and compare its average RCS to a relevant bird size target to be able to assess the test data recorded when the aircraft is used in avian radar performance testing. Therefore a near-field measurement of RCS was considered to be sufficient for our purpose.

3.2 Measurement set-up

The model aircraft was fitted with a special designed PVC support bracket and mounted on the turntable on the support rod of the measurement tower in the anechoic room. The tower with its motion controller is capable of rotating the measurement objects around three axes, but in our case only the belt-driven turntable azimuth movement was used, and the pitch of the aircraft was set by a special adjustment feature of the mounting bracket. This provided “great circle RCS cuts” of the aircraft when it was rotated 360° in azimuth, which implies that all the RCS cuts will pass through the same point broadside, i.e. when the aircraft is pointed at +/- 90° from the antennas. Pictures of the aircraft mounted on the measurement tower in the anechoic room are in Figure 3 below.

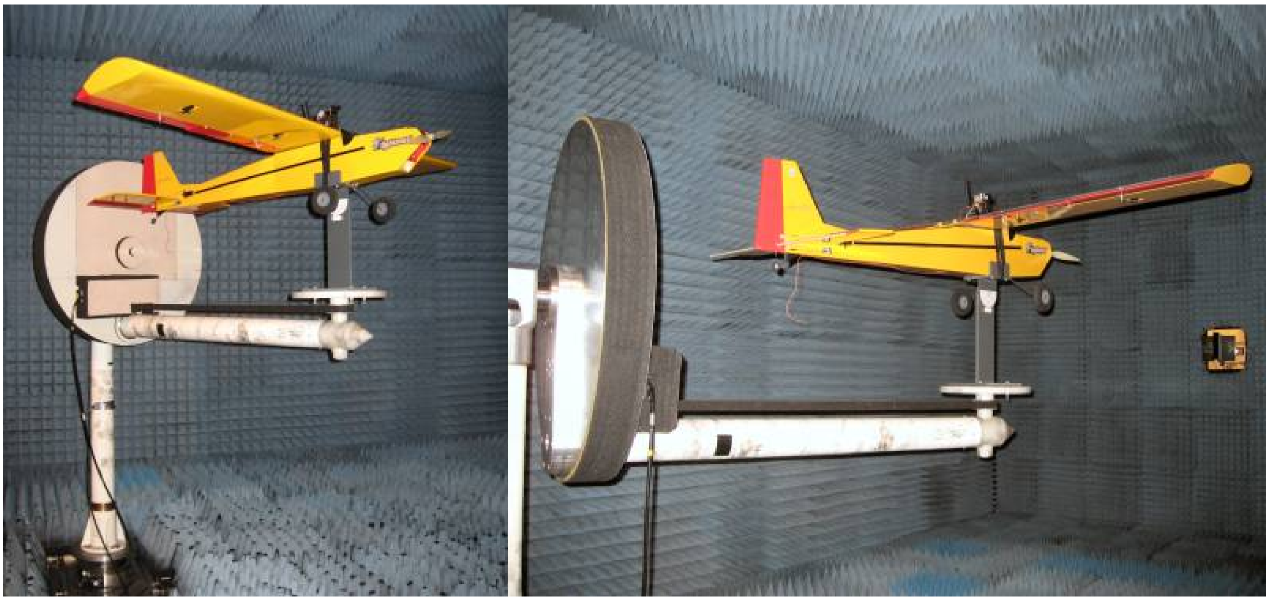

Figure 3 Aircraft in anechoic room

The radar antennas can be seen on the far right, mounted in an opening in the end wall in the anechoic room. An RF-absorber element was placed between the antennas to improve isolation between the transmitter and the receiver.

A schematic overview of the test set-up with the instruments and interconnections used is in Figure 4 below.

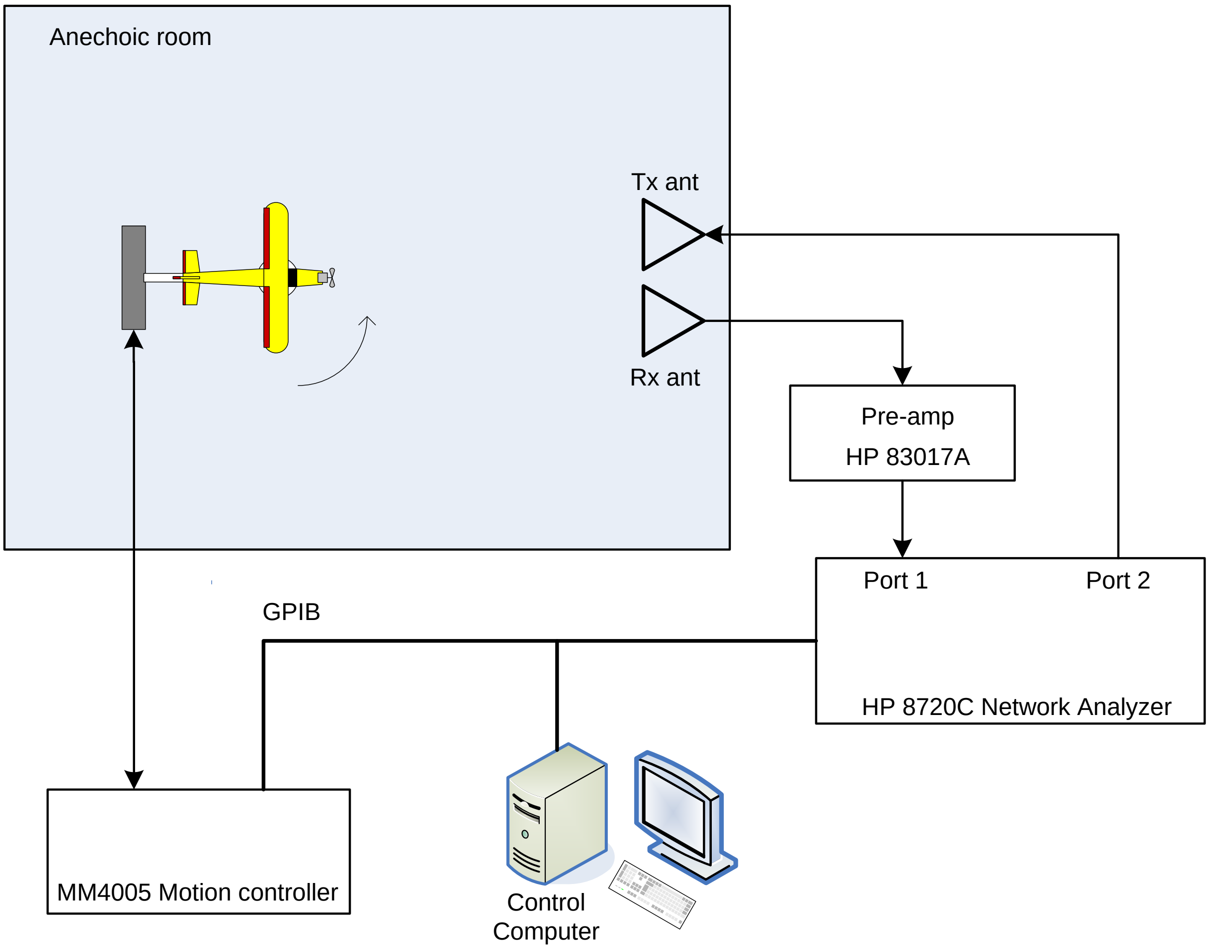

Figure 4 RCS measurement set-up

An equipment list for the measurement set-up is in Appendix B. The HP 8720 network analyzer functioned as the "radar" in this test. This instrument is normally used to measure the scattering parameters of two-port electronic devices, e.g. RF-amplifiers. In this case we measured and recorded the transmission parameter S12, which provides the transfer function, amplitude and phase, from port 2 to port 1 of the instrument, and used this as the target response to be analysed. I.e. the output from port 2 was the radar transmitting waveform and port 1 the receiver. The HP83017A is a broad-band amplifier with about 36 dB gain and 5 dB noise factor for both S- and X-band. The amplifier was used as a pre-amplifier in the receiving chain to increase the system sensitivity. Use of this amplifier in the receiver chain causes only a negligible increase in the network analyzer receiver noise floor, thus maintaining the overall system dynamic range.
With this set-up, the received power at port 1 of the network analyzer from a 0.01 m^2 target at a distance of 6 m is about -45 dBm in S-band and about -50 dBm in X-band. This should provide sufficient margin to the network analyzer noise floor of -93 dBm and at the same time be well below the maximum network analyzer receiver input power of 10 dBm, even with the largest targets and strongest returns from structures inside the room. The measurement uncertainty of the network analyzer at levels of -50 to -60 dBm is better than 0.2 dB according to [5]. A more detailed link budget for both the relevant frequencies can be found in Appendix C.

The Newport MM4005 motion controller sets and steers the movement of the three axes of the measurement tower, and can be controlled manually from a front-panel, or remotely via the GPIB interface.

The interior of the anechoic room is covered with cones of RF absorbing material which has a lower cut-off frequency of 2 GHz. But even at frequencies well above the lower cut-off, the return from the room itself and structures inside it, especially the measurement tower, is larger than the echo from the actual target to be measured. To be able to detect and separate the return of the model aircraft from the unwanted response of the room itself, two responses were recorded for each RCS measurement, first the room with the object inside, and secondly the response from the empty room with the object removed. Then the response from the empty room was coherently subtracted from the response including the object. In theory this difference should provide the return from just the object alone. But there is a couple of error sources associated with this “method of subtraction” that needs to be taken into account. First there may be indirect reflections from the object (e.g. from the object via the measurement tower), that cannot be removed by subtraction because they will only be present when the object is in the room. However, the random errors introduced by this effect seem to be only minor for the frequencies and objects measured. Secondly, there are moving mechanical devices on the measurement tower, cog wheels and belt, which will give a slight and unpredictable variation in the total response from the tower from one frequency sweep to the next, when the network analyzer is set to sweep over at certain bandwidth for each azimuth position of the target. I.e. the method of subtracting the empty room response from the response with the object in place, cannot cancel the tower response completely and a small echo from the tower will remain. For S-band this residue was small, but for X-band it was found to be in the same order of magnitude as the smallest calibration object, and the tower response had to be removed from the data by gating in the time domain before the RCS could be estimated. See paragraph 3.3 below.

The measurements and recording of one 360° cut of RCS data is fully automated and controlled by MATLAB with a special script running on a Windows Vista PC. The PC communicates with the motion controller and network analyzer on a GPIB bus, and synchronizes the movement of the object with the network analyzer measurements and reads and records the data from the network analyzer for each specified azimuth position. All measurements are recoded and stored in MATLAB format for subsequent analysis.

3.3 Estimation of RCS

The RCS measurement performed with this method falls under the class of “coherent Continuous Wave (CW) measurements”, which provides the same RCS as would be seen with a pulse that was long enough to bracket the target with a “comfortable margin” [3]. The minimum pulse length of the NINA radar at Smøla is 80 ns, which means that it provides a theoretical range resolution of 12 m, which is longer than the maximum extension in range of the model aircraft and any single bird target with plenty of margin. Thus the CW measurement of target RCS at the nominal radar centre frequency, or an average of a few separate frequencies close to the centre frequency, will provide the same results as if the target RCS was measured with the relatively long pulse width of the radar system.

However, the network analyzer was set to measure the S12 parameter at distinct frequencies equally spaced by 10 MHz over a bandwidth of 1 GHz in the relevant radar bands. This allowed us to generate a time response, or “range-profile” with an unambiguous range of 15 m and a theoretical range resolution of 15 cm. High resolution time domain analysis is useful for identifying the individual scatterers on the aircraft, but can also be used gate out any large unwanted echoes from other structures in the anechoic room that lags or leads the target response

by more than 15 cm. When the unwanted echoes have been gated out in time, the signal can be transformed back to the frequency domain and the RCS estimated at the relevant frequencies. The largest unwanted return from the anechoic room proved to be from the main structure of the measurement tower. The distance from the tail fin of the aircraft to this part of the measurement tower was only about 25 cm when the aircraft was pointed 0° (head-on) towards the antennas. See Figure 3 above. I.e. with a measurement bandwidth of 1 GHz, it was be possible to separate the aircraft echo from the tower return in the time domain. The direct response from the measurement tower at X-band, had to be removed from the data using this procedure.

The RCS could also be estimated from the range profile in the time domain, but in that case the time domain response would have to be generated with a resolution larger than the maximum target extension in range with a certain margin, i.e. more than 2.1 m, and then we would not be able to resolve the target from the measurement tower response which lags the target response by only 25 cm.

3.4 Calibration

The measurement of the absolute RCS of a target requires a radar with a calibrated output. Calibration implies that we must be able to relate the output from our radar to a physical parameter of the target, in this case its RCS. In principle, if all the parameters in Eq. 1 were known, the measured power level could be used to calculate the RCS directly. This is the method that has to be used in most dynamic RCS test ranges. But in our case, since this RCS measurement was to be performed with the target in a fixed position, we could take advantage of that fact and calibrate the measurement by replacing the object with unknown RCS with objects which have known RCS. The response in the receiver from these calibration targets was then recorded and compared to the response from the actual target. This is sometimes referred to as the “method of substitution” [3], and the advantage of this calibration method is that we don’t need detailed information on all the radar parameters. But it does require that the system transfer function is also recorded. In this case we assumed a linear amplitude transfer function, which could be estimated using different size calibration objects.

The calibration objects must be simple shapes with a RCS which is easy to determine for the relevant frequencies. In this test the following calibration objects have been used:

- A steel sphere with a radius of 5.9 cm having a RCS of -18.8 dBm^2 and -19.6 dBm^2 in S- and X-band respectively. In S-band the sphere will be in the resonance region.
- A Styrofoam sphere covered with aluminium tape with a radius of 7.5 cm. This sphere has been used as a towed test target in live radar tests, and it will have a RCS of -16.1 dBm^2 and -17.5 dBm^2 in S- and X-band respectively. In S-band the sphere will be in the resonance region.
- A small trihedral corner reflector with edges 14 cm and with a RCS of -4.0 dBm^2 in X-band. The simple formula used to calculate the RCS of corner reflectors requires that the wavelength is much smaller than the dimensions of the reflector. The S-band wavelength is about 10 cm, thus preventing the use of the simple formula. The RCS in S-band can therefore not easily be determined for a corner reflector of this size, and consequently it has not been used to calibrate the S-band measurements.
- A medium size trihedral corner reflector with edges 25 cm giving a RCS of -3.7 dBm^2 and 6.1 dBm^2 in S- and X-band respectively.
- A large trihedral corner reflector with edges 70 cm giving a RCS of 14.2 dBm^2 and 23.9 dBm^2 in S- and X-band respectively. However the size of this corner reflector requires it to be 10.0 m away from the antennas at S-band and 30.8 m at X-band for it to be in the far-field. The distance from the antennas to the turntable with the calibration

objects in the anechoic room is only a little over 6 m. This corner reflector has therefore not been used to calibrate the X-band measurements, but since the dimensions of the effective reflecting area of the corner reflector is significantly less than the dimensions of the corner reflector itself, it has been considered to be close enough to the far-field requirement at S-band, and therefore used for the calibration in this radar band.

The 5 objects used to calibrate the RCS measurements, three trihedral corner reflectors and two spheres, are in Figure 5 below.

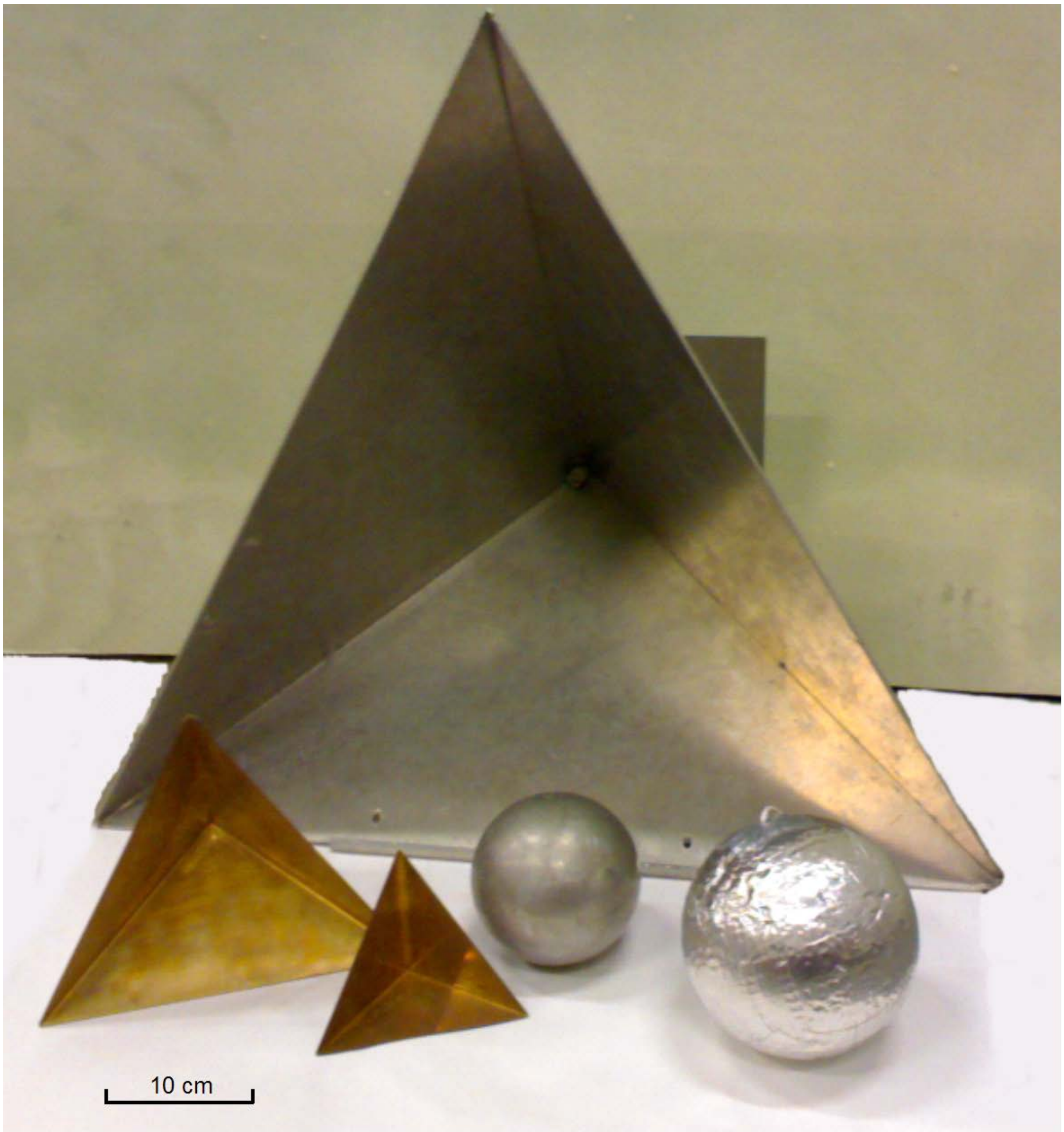

Figure 5 Calibration objects

4 Measurements

In S-band the RCS measurements have been made for horizontal polarisation only, while in X-band both vertical and horizontal polarisation have been measured since the X-band radar in the NINA radar system at Smøla can be configured to scan either horizontally or vertically. For both radar bands the RCS measurement set-up described in paragraph 3.2 was configured to rotate the aircraft 360° in azimuth and for every 2° record the S12 parameter at 101 discrete frequencies

spaced 10 MHz apart over a 1 GHz bandwidth around each nominal radar frequency. The total 360° azimuth scan was automatically run and recorded. The port 2 output of the network analyzer was set to output 13 dBm to provide an additional 3 dB of margin to the noise floor at port 1, compared to what has been used in the link budget in Appendix C. It was verified that this output was within the linear range of the output amplifier. The elevation aspect angle was set manually for each 360° scan by adjusting the pitch of the aircraft on the aircraft mounting bracket. See Appendix A. The angle was varied in steps of 5° from -20° (the radar looking up at the aircraft) to 20° (the radar looking down at the aircraft). See Figure 6 below.

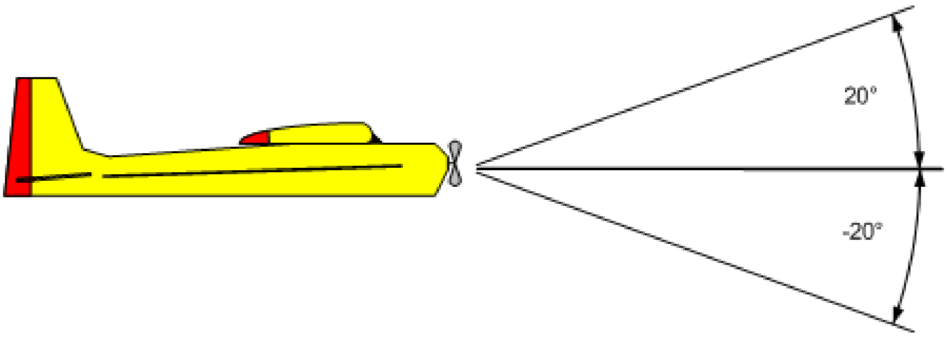

Figure 6 Elevation aspect angles

4.1 S-band

4.1.1 S-band calibration

The S-band calibration was performed using the two largest corner reflectors and the two spheres, as described in paragraph 3.4. The S12 parameter was measured for each calibration object and in this way samples of the transfer function of the system were provided. A complete 360° azimuth scan was performed for all objects. The estimates of S12 from the two corner reflectors was formed by taking the average of the returns at the nominal radar frequency at 3050 MHz and the two neighbouring frequencies in the measured frequency array, i.e. at 3050 MHz +/- 10 MHz. This estimate was taken at the peak of the azimuth RCS pattern at 0° when the corner reflector was facing the antennas. The estimates of the spheres were in addition to the frequency average, also averaged over all azimuth position since the RCS of these objects is independent of aspect angle.

The S12 estimates were plotted versus theoretical RCS for each target and a line was fitted to the data. Se Figure 7 below.

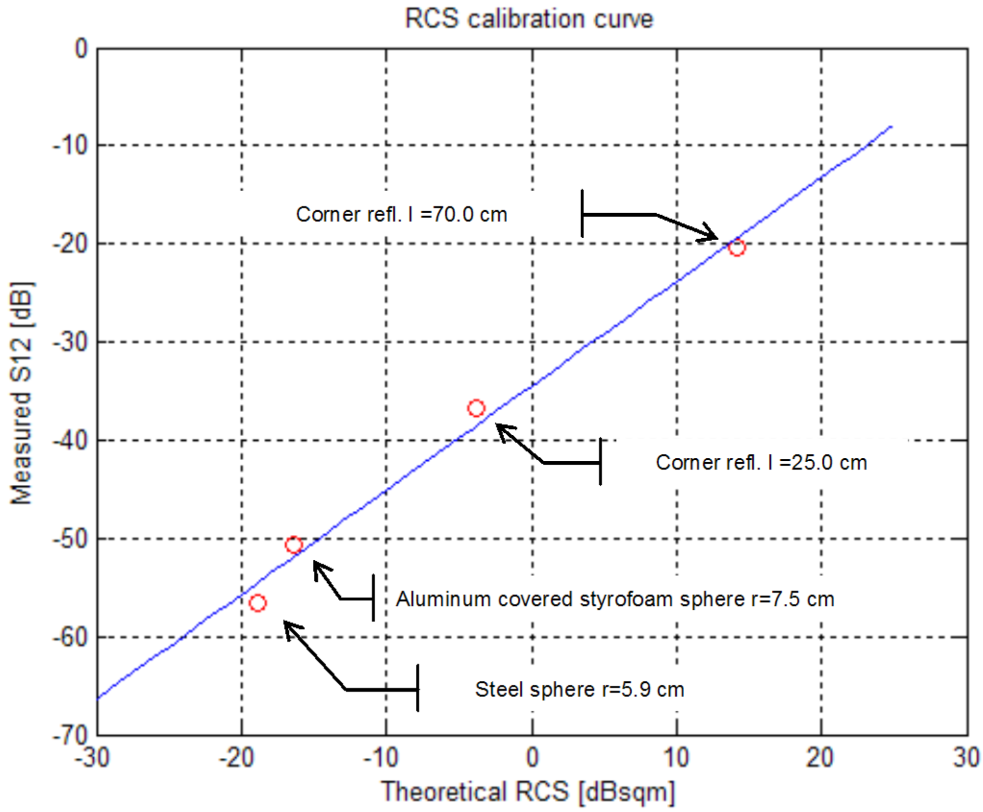

Figure 7 S-band calibration

The line became the estimate of the system transfer function, and was used to map the measured S12 levels from the model aircraft into RCS values.
All the measurement points are within 1-2 dB away from the fitted line, and this error distance provides an indication of the accuracy of the RCS data obtained with this method.

4.1.2 S-band RCS measurements

A calibrated polar plot of model aircraft RCS in S-band as a function of azimuth aspect is in Figure 8 below.

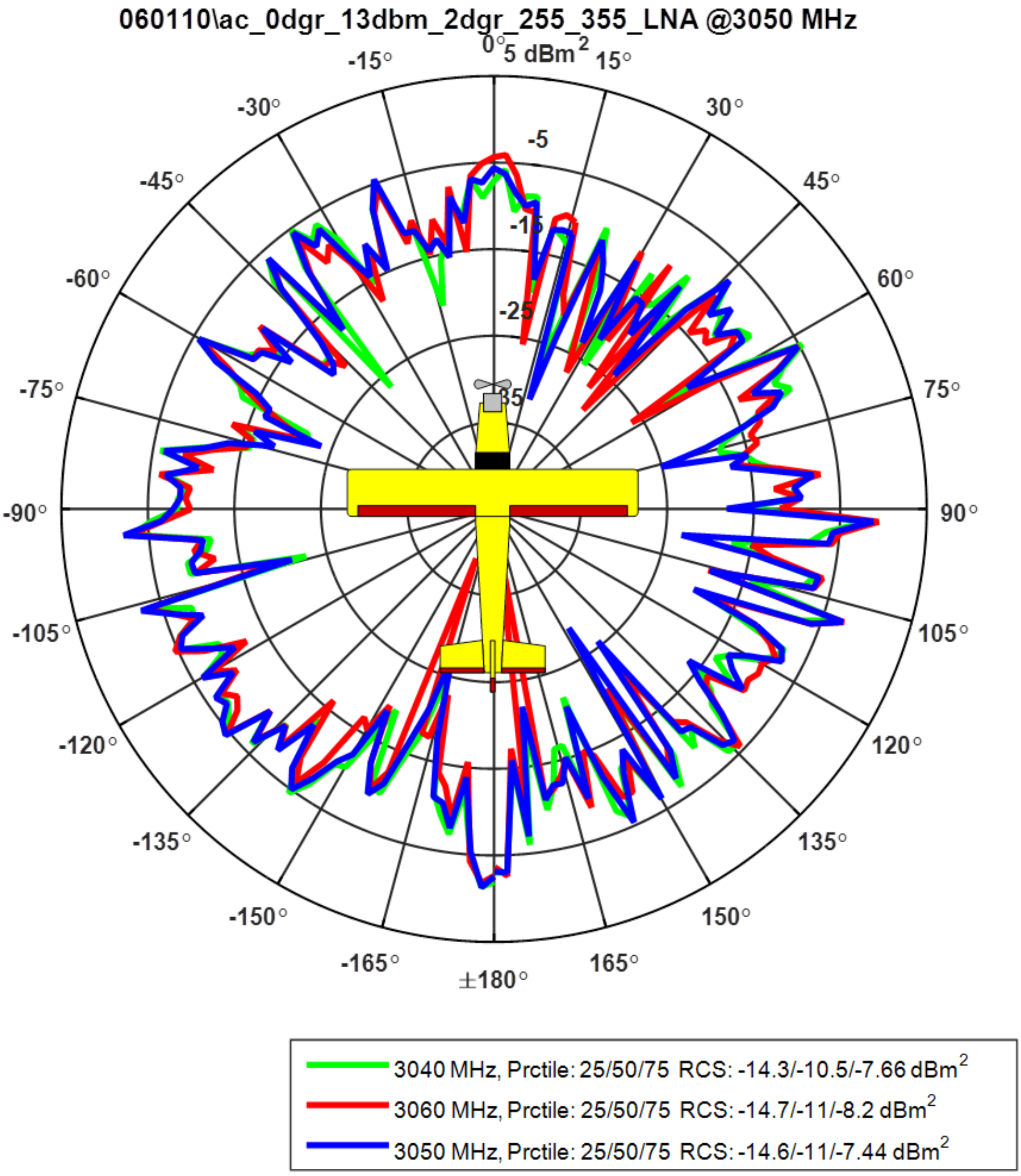

Figure 8 Aircraft S-band RCS at 0° elevation

In this measurement the aircraft had 0° pitch. RCS is plotted for the nominal S-band frequency of 3050 MHz (blue trace) together with the corresponding measurements at 3040 and 3060 MHz, giving an indication of the RCS variation over a band of 20 MHz around the centre frequency. The median RCS at 3050 MHz over 360° is -11.0 dBm^2, i.e. 0.079 m^2. The aircraft has distinct lobes head-on, tail-on and from the sides, but these lobes are not very much larger than lobes in other directions, which points to the conclusion that it is not the balsa and plywood wings and fuselage itself that causes the biggest returns, but rather the wiring and the different parts mounted both inside and outside the aircraft (See Figure 1 and Appendix A). Another indication of this can be found if the range profile for each azimuth position is plotted in a colour intensity plot.

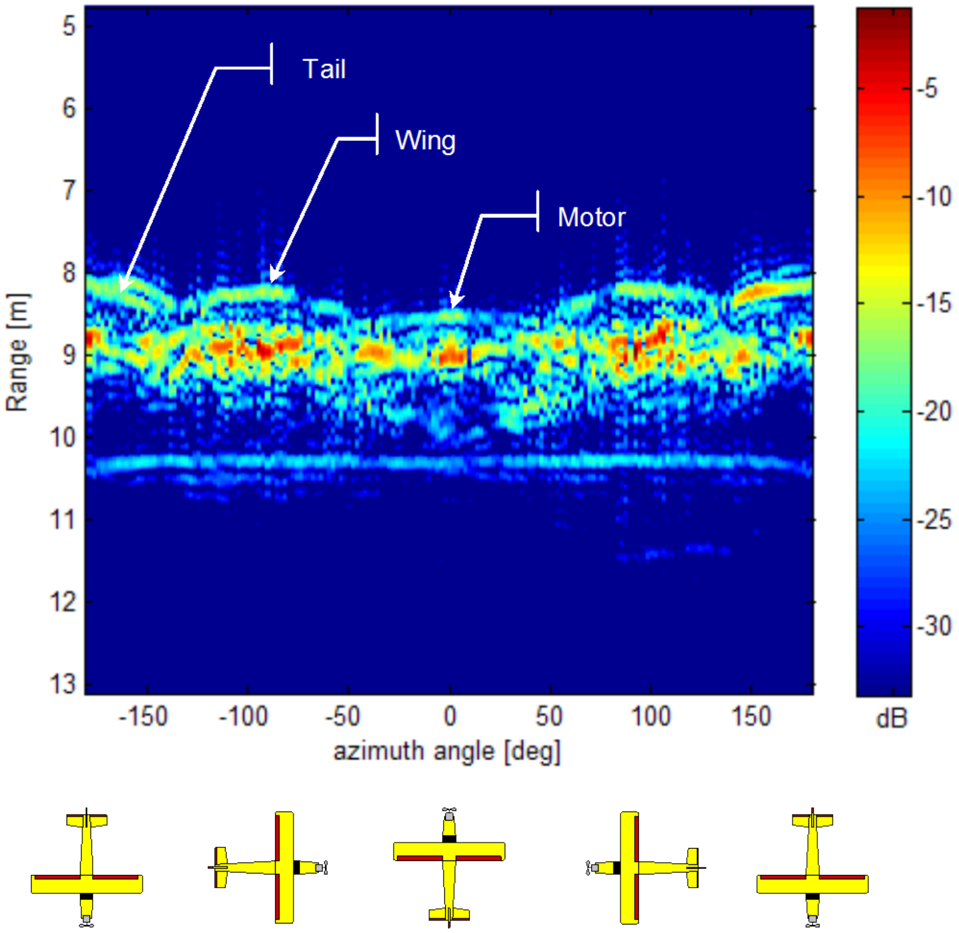

Figure 9 S-band range profiles, aircraft at 0° elevation

In this plot the response from the tail, wing and motor, are easily distinguishable from the rest of the response from the aircraft. The weak horizontal line at about 10.5 m is the remainder of the echo from the measurement tower which has not been completely removed with the subtraction method described in paragraph 3.2. To verify that it is actually the outer parts of the wing that cause the response indicated in Figure 9, the wing with the video camera was removed and the RCS at 0° elevation measured once more with the same set-up.

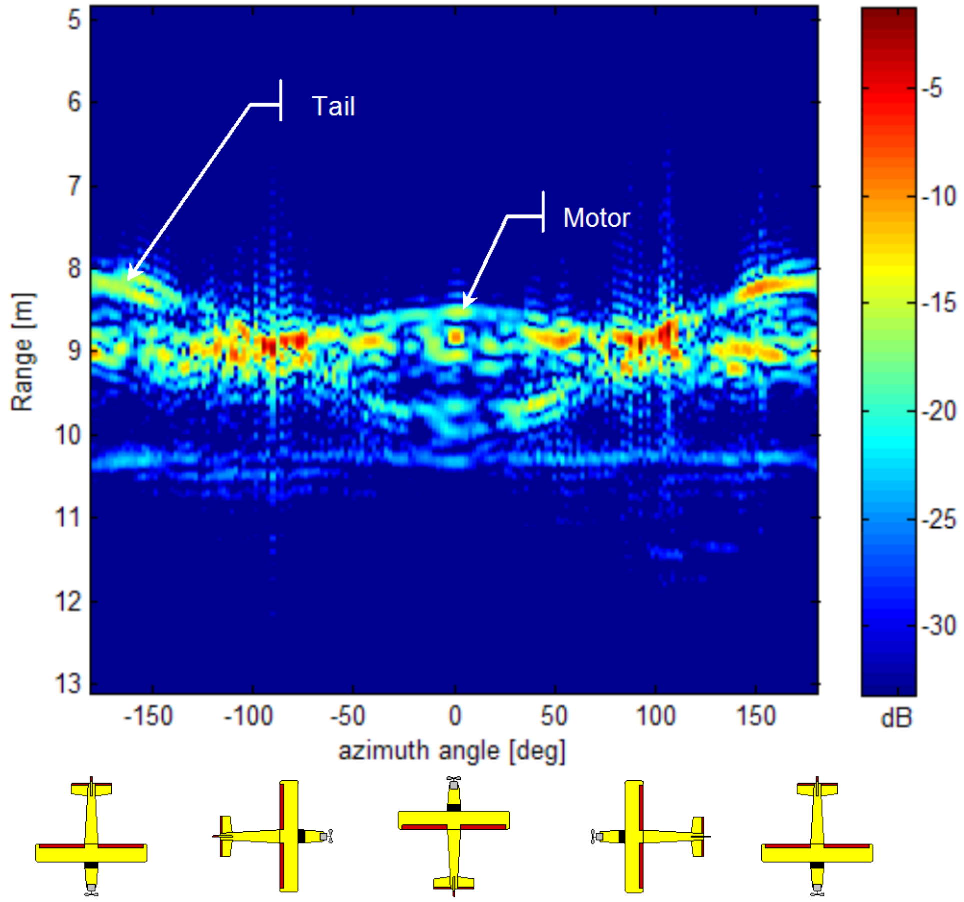

Figure 10 S-band range profiles, aircraft at 0° with wing removed

Now the response from the parts of the wing is gone and the cosine-like trace from the tail as the aircraft swings round on the turntable becomes more evident. But no also the return from the central part of the aircraft is reduced, at least head-on. Part of this reduction is due to the video camera on the top of the wing which is also removed when the wing was dismounted.
The vertical lines in the portions of the image that has the strongest returns, are sidelobes from the fast fourier transform (fft) used to generate the range profiles.

A new 360° RCS plot of the aircraft with the wing and video camera removed is in Figure 11 below.

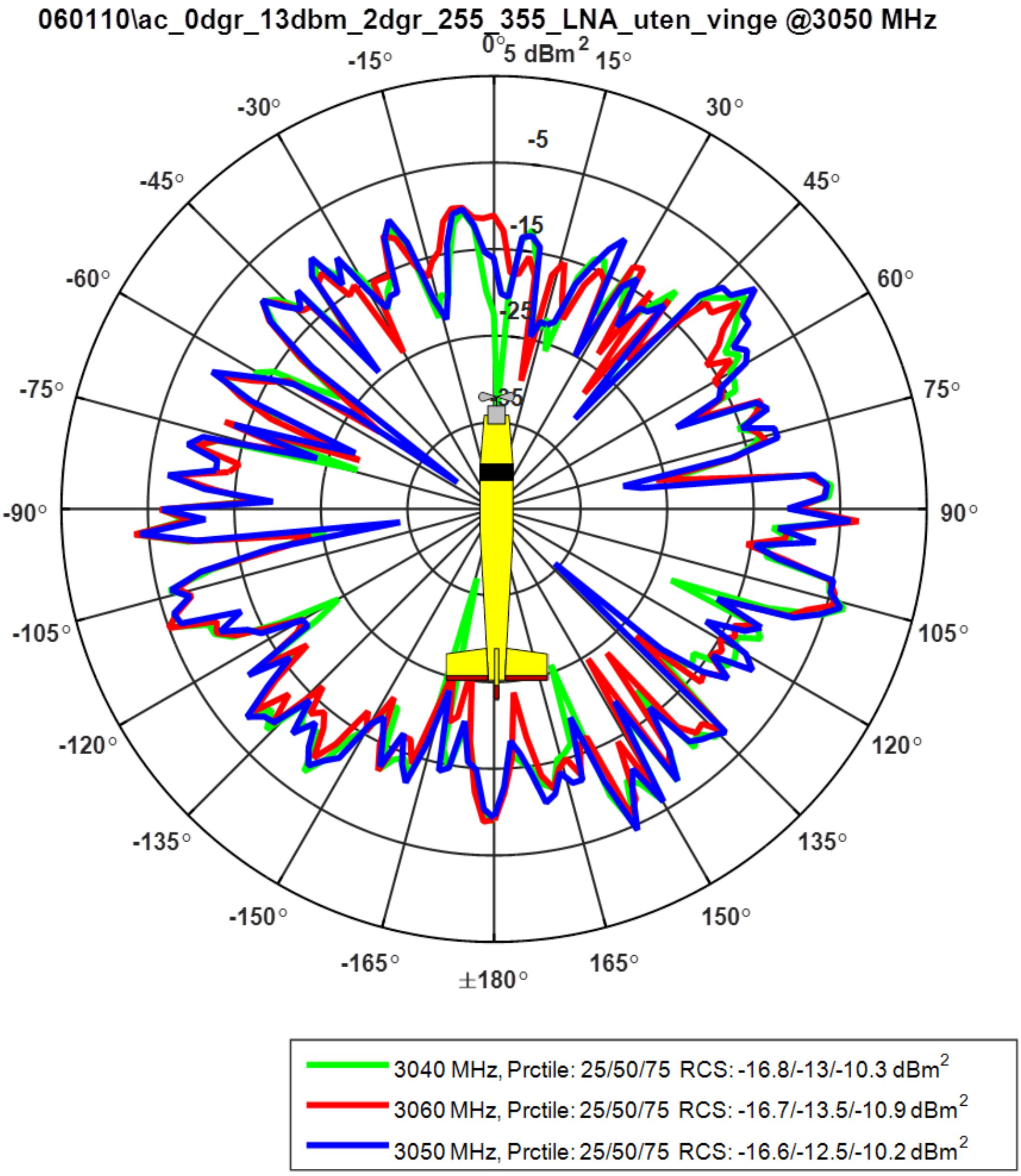

Figure 11 Aircraft RCS with wing and video camera removed

In this measurement the head-on and tail-on lobes are much lower, and there is a noticeable reduction in the 50 percentile RCS from -11.0 dBm^2 to -12.5 dBm^2 (0.056 m^2) at 3050 MHz, which implies that the wing and the video camera are in fact significant contributors to the total RCS. However, the outer part of the wing seems to scatter independently from the central parts of the wing and aircraft, which in case confirms the validity of this method of performing RCS measurements in the near-field of the model aircraft.

The variation of RCS with elevation angle is only minor which again points to the conclusion that it is not the balsa and plywood fuselage that is the largest scatterer, but rather the different parts mounted inside and outside the aircraft, which are relatively small compared to the wavelength and therefore have an RCS which is less sensitive to changes in aspect angle. But, referring to the discussion in paragraph 3.2, one should also bear in mind that the RCS cuts at different elevation

angles all pass through the same point on the side of the aircraft. Plots of aircraft S-band RCS for various elevation angles are in Appendix D.

4.2 X-band

4.2.1 Horizontal polarisation

4.2.1.1 X-band horizontal polarisation calibration

Since the largest corner reflector with sides 70 cm would be in the near-field at X-band, the X-band calibration was performed using the two smallest corner reflectors and the two spheres, as described in paragraph 3.4. At X-band, the residue of the echo from the measurement tower after subtraction of the empty room response was much larger than at S-band.
Figure 12 below is a time response plot of the response from smallest sphere together with the residue from the measurement tower.

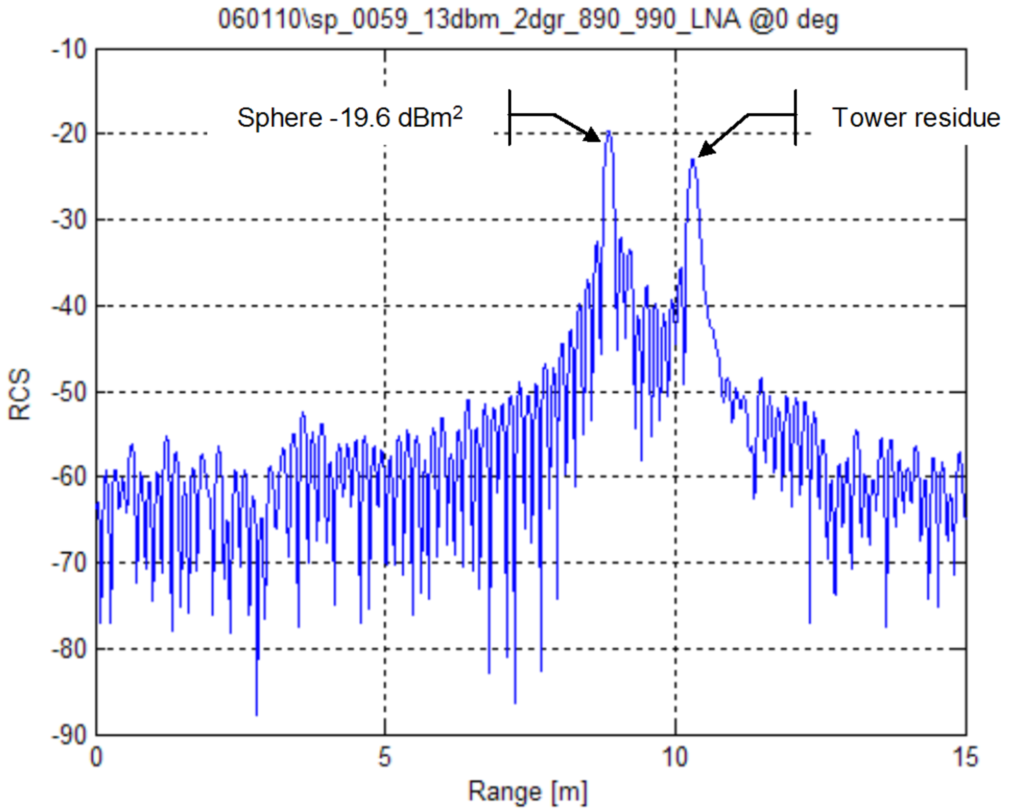

Figure 12 Time response X-band vertical, sphere -19.6 dBm^2 and tower residue

This residue is believed to be caused by the small moving mechanical parts, belt and cog wheels, which will have a different and unpredictable position from one measurement to the next. Since it cannot be removed by simple subtraction, it is gated out in time in the data sets used for X-band RCS estimation. The resulting range profile with only the response from the smallest calibration object is in Figure 13 below.

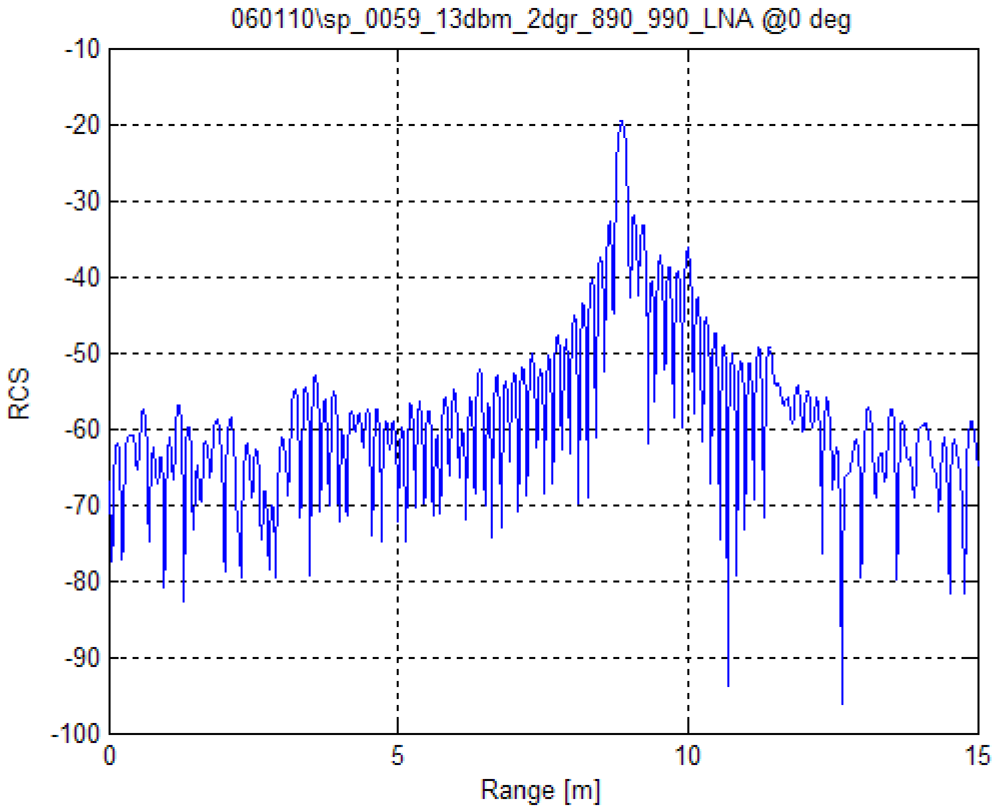

Figure 13 Time response X-band vertical after gating of measurement tower

The calibration procedure for X-band was otherwise the same as for S-band described in paragraph 4.1.1. The S12 estimates were plotted versus theoretical RCS for the four calibration objects and a line fitted to the data.

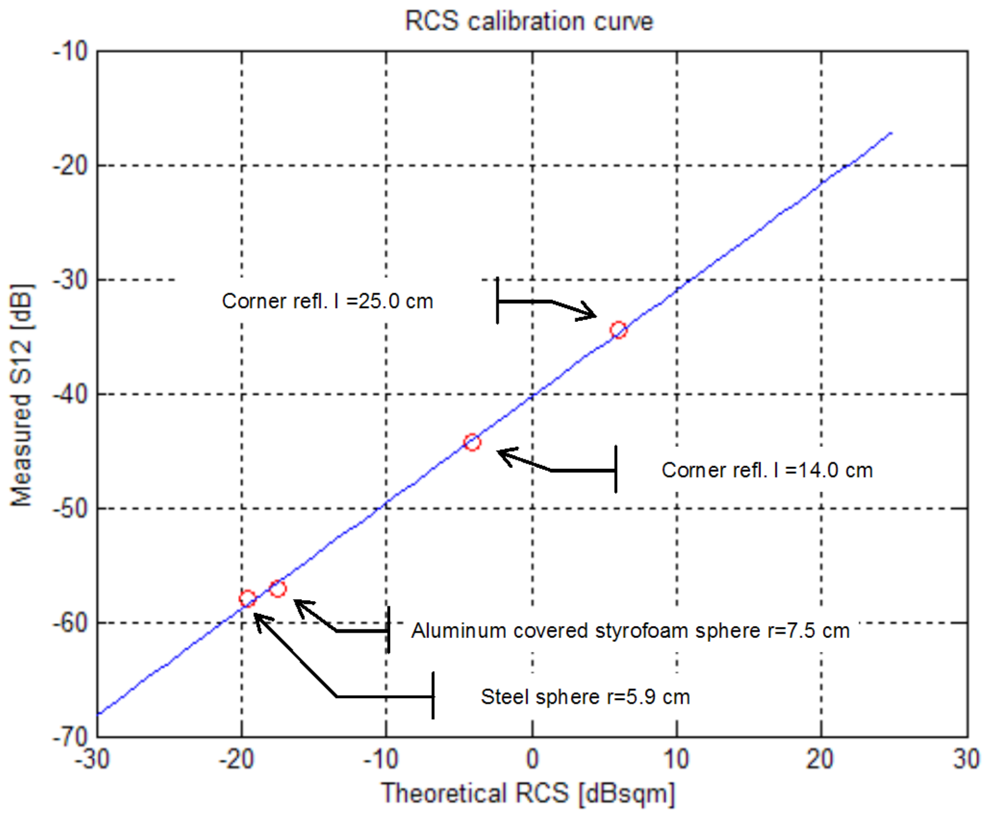

Figure 14 Calibration X-band horizontal

In this case the line fitted the data very well and all the calibration targets are within 0.5 dB of the estimated transfer function.

4.2.1.2 X-band horizontal polarisation RCS measurements

A calibrated polar plot of model aircraft RCS in X-band horizontal polarisation as a function of azimuth aspect is in Figure 15 below.

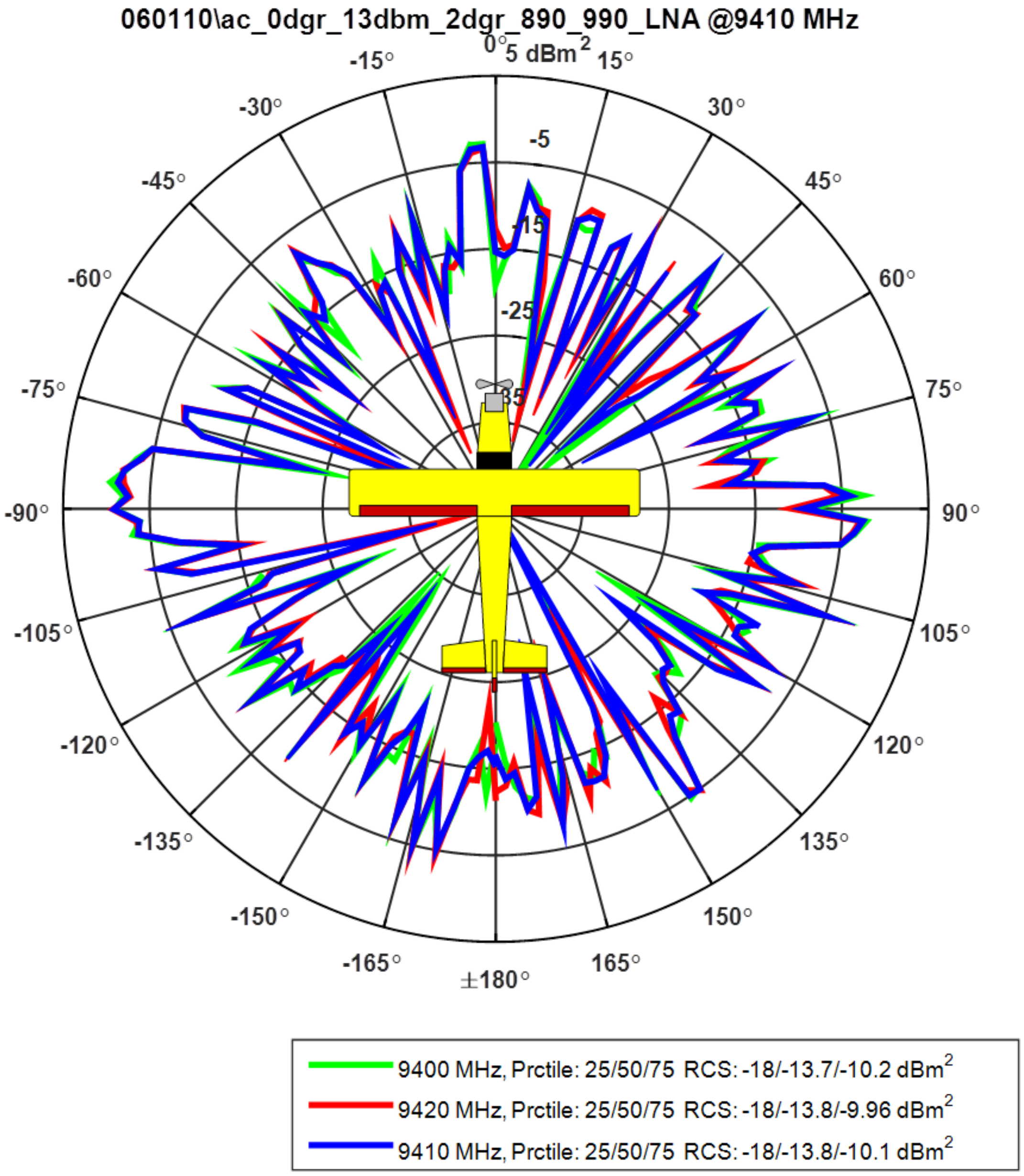

Figure 15 Aircraft X-band horizontal polarisation RCS at 0° elevation

The 50 percentile RCS at the nominal radar X-band frequency, over 360°, is estimated at -13.8 dBm^2, (0.042 m^2) which is a little less than in S-band. However the lobes in the pattern at broadside (+/-90°) seem to be more distinct than in S-band. This impression is confirmed if we plot the time response for each azimuth position in a colour intensity plot.

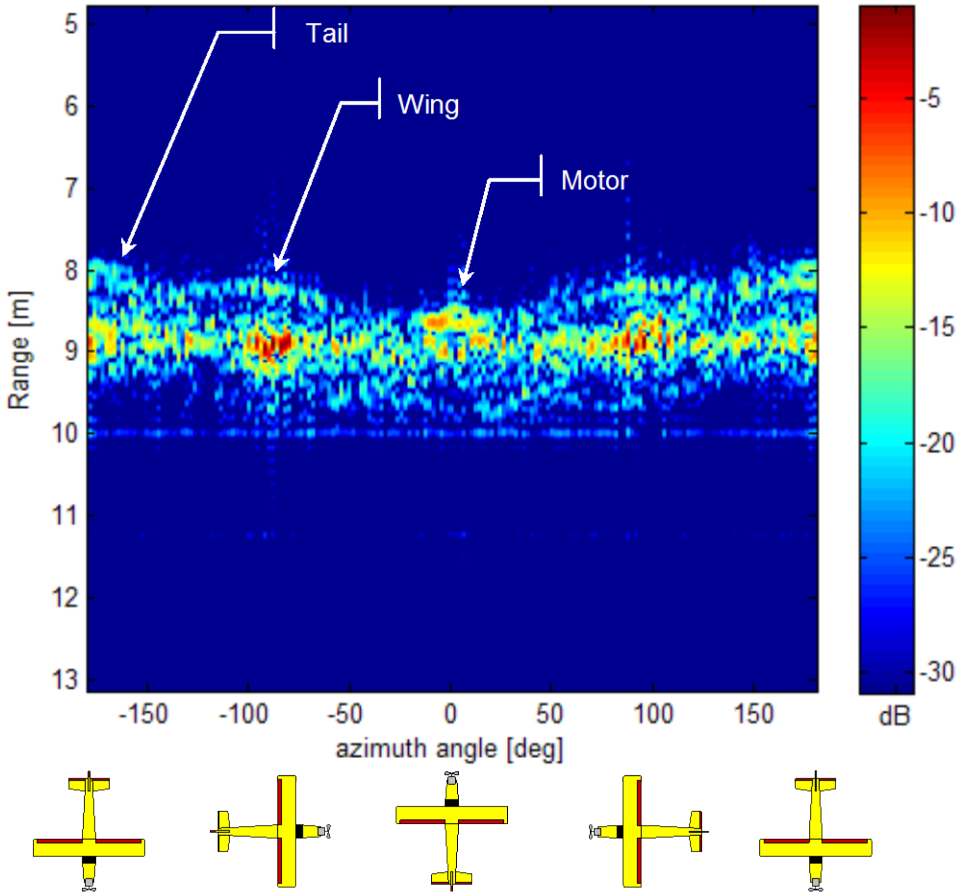

Figure 16 Range profile X-band horizontal, aircraft at 0° elevation

Here we can see that the most intense areas of scattering are when the aircraft is viewed broadside at +/- 90°. Also at X-band it is easy to distinguish the response from the tail, wings and motor in the time response, which is verified if the same RCS measurement is performed with the wing and video camera removed.

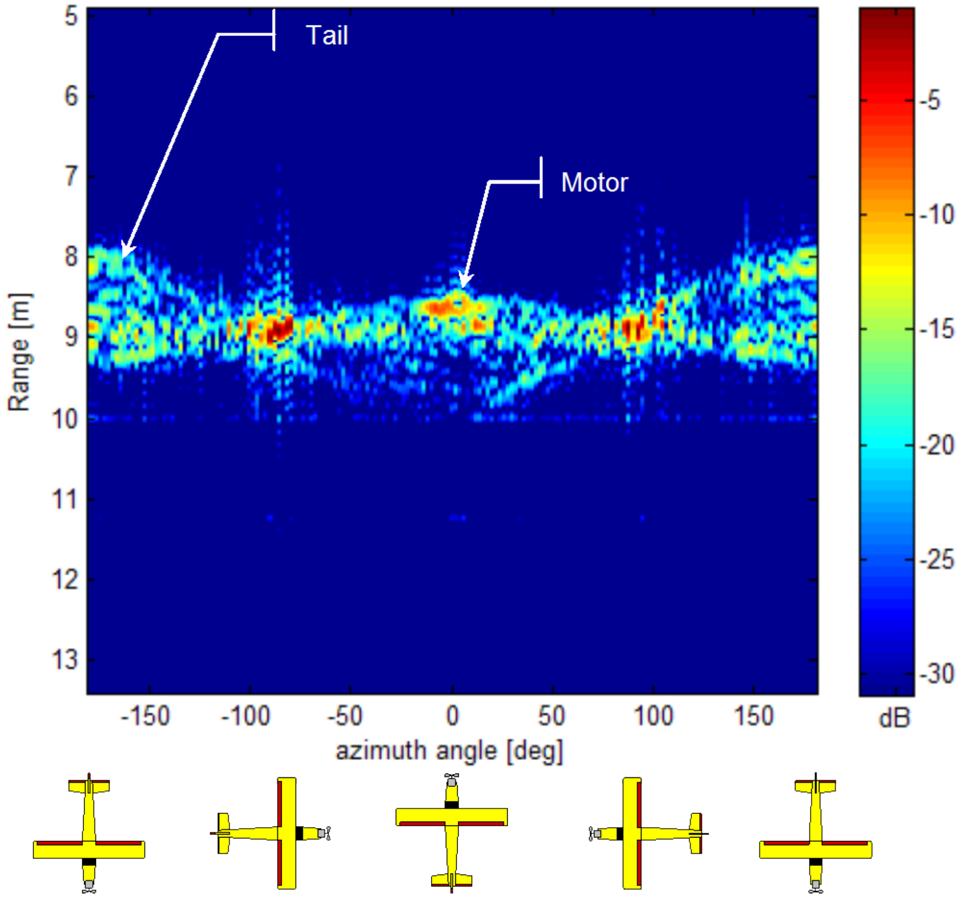

Figure 17 Range profiles X-band horizontal with wing removed

The response from the wings is disappeared and the major lobes at head-on, and broadside (+/-90°), become even more evident.

A new 360° RCS plot of the aircraft with the wing and video camera removed is in Figure 18 below.

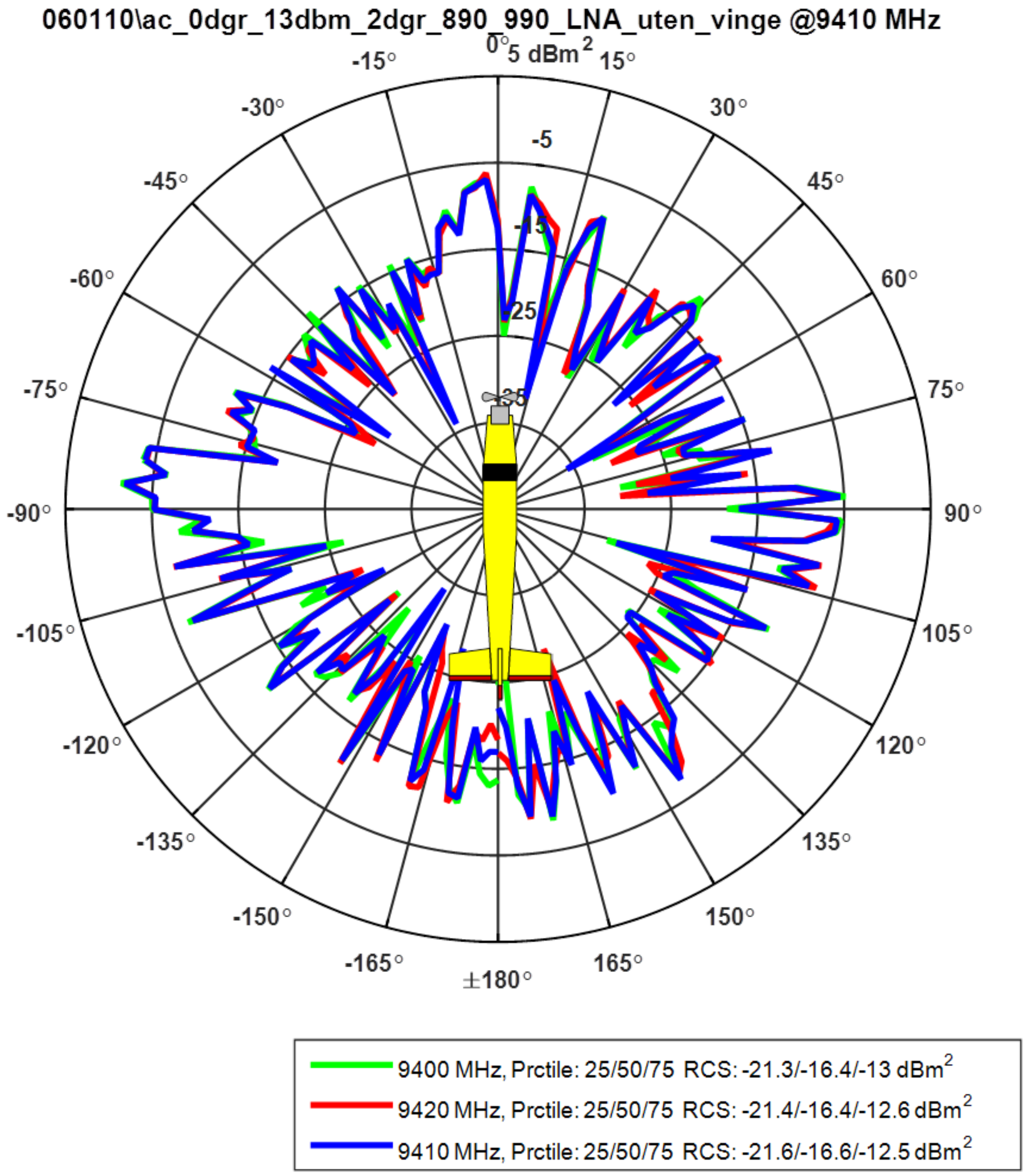

Figure 18 Aircraft X-band horizontal RCS with wing and video camera removed

Now the 50 percentile RCS at 9410 MHz is reduced form -13.8 dBm^2 with the wing to -16.6 dBm^2 (0.022m^2) with the wing and video camera removed. This is approximately same reduction that was seen in S-band for the same test.

At X-band the higher broad side lobes may suggest that the scattering from the balsa fuselage is higher than at S-band. However, several of the individual parts inside and outside the aircraft have a larger broadside area that may contribute to this effect. The battery pack is a relatively large rectangular box that falls into this category. (Refer to Figure 1) The battery pack is also easy to remove, and the RCS plot in Figure 19 is the X-band horizontal RCS of the aircraft without the battery pack, but including the wings.

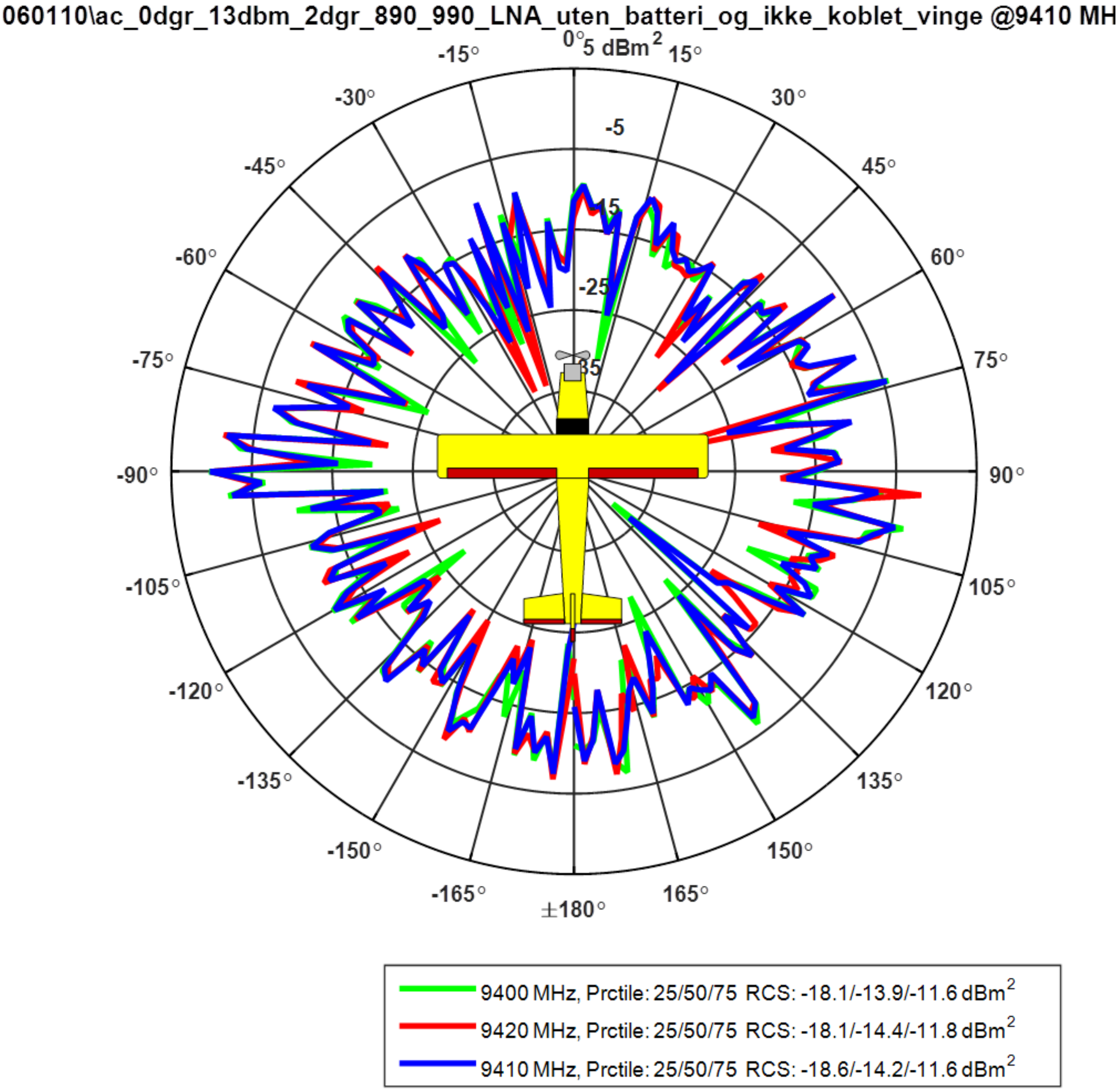

Figure 19 Aircraft X-band horizontal RCS with battery pack removed

There is only a minor drop in 50 percentile RCS, but the lobes at broadside are significantly reduced indicating that the battery pack in fact contributes to this effect.

At X-band, there seem to be somewhat higher variation in RCS (around 3 dB) with elevation angle compared to what has been recorded in S-band. An explanation for this may be that the wavelength at X-band is small compared to the scatterers, and this causes larger variation in RCS with aspect angle for each individual scatterer. Plots of aircraft RCS, X-band horizontal polarisation, for various elevation angles are in Appendix E.

4.2.2 Vertical polarisation

4.2.2.1 X-band vertical polarisation calibration

The calibration at X-band vertical polarisation used the same procedure as X-band horizontal polarisation calibration described in paragraph 4.2.1. The S12 measurements of the four calibration objects together with the estimated transfer function are in Figure 20 below.

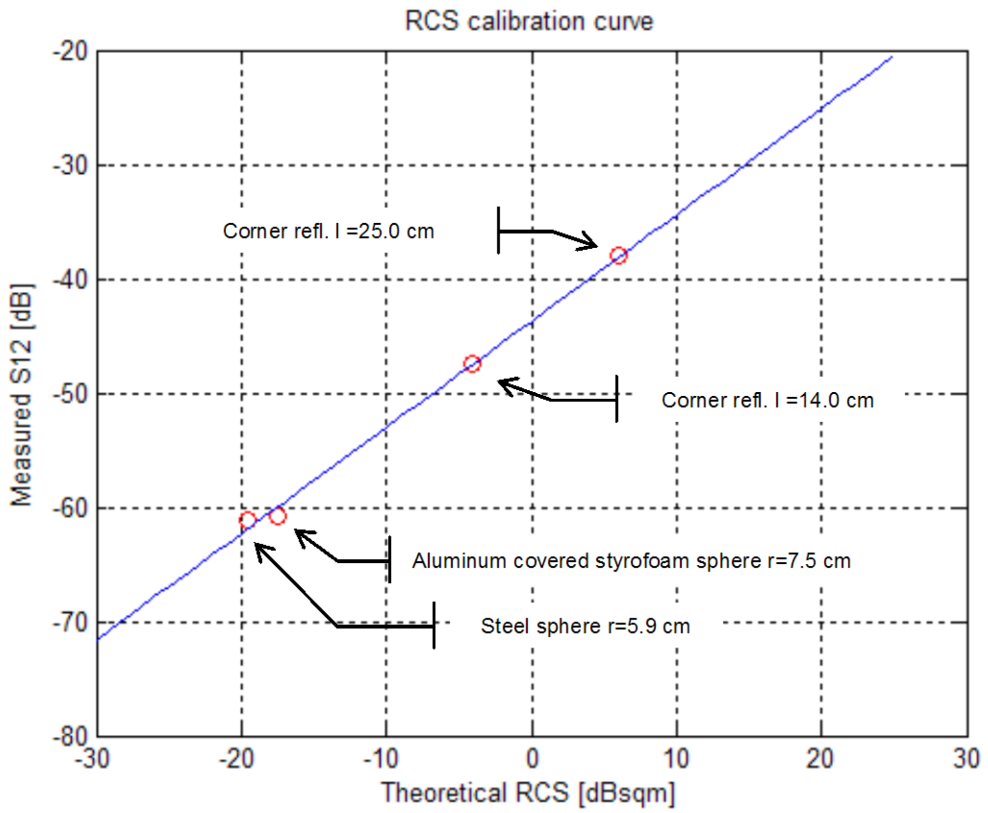

Figure 20 Calibration X-band vertical

Also for X-band vertical polarisation, the line fitted the data well and all the calibration targets are within 1 dB of the estimated transfer function.

4.2.2.2 X-band vertical polarisation RCS measurements

A calibrated polar plot of model aircraft RCS in X-band horizontal polarisation as a function of azimuth aspect is in Figure 21 below.

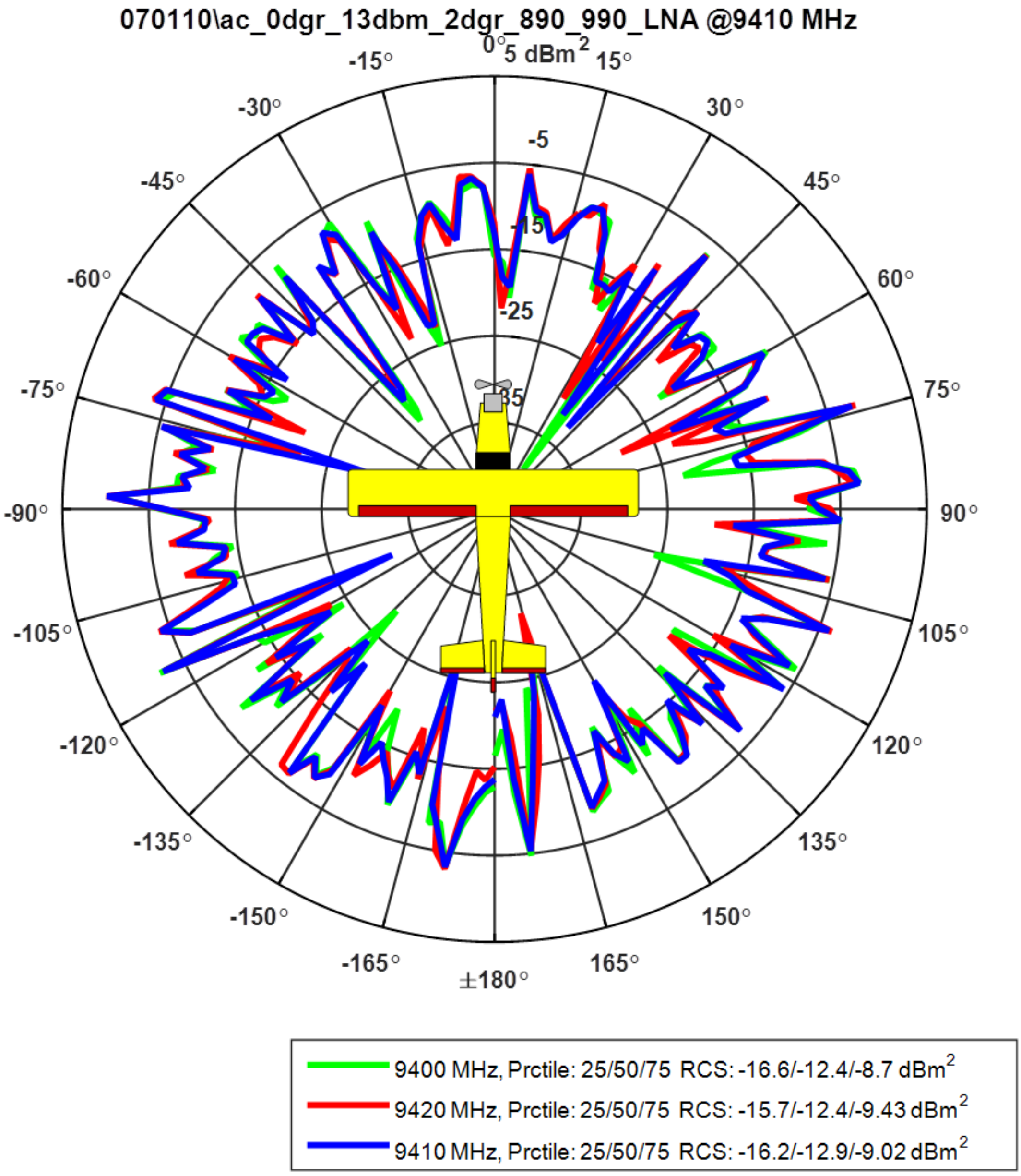

Figure 21 Aircraft X-band vertical polarisation RCS at 0° elevation

For vertical polarisation the X-band 360° 50 percentile RCS is -12.9 dBm^2 (0.051m^2) which is 1 dB more than X-band horizontal polarisation. The lobe pattern resembles the S-band pattern without the large lobes at broadside that was seen in case of X-band horizontal polarisation. A colour intensity plot of the range profiles for each azimuth position is in Figure 22 below.

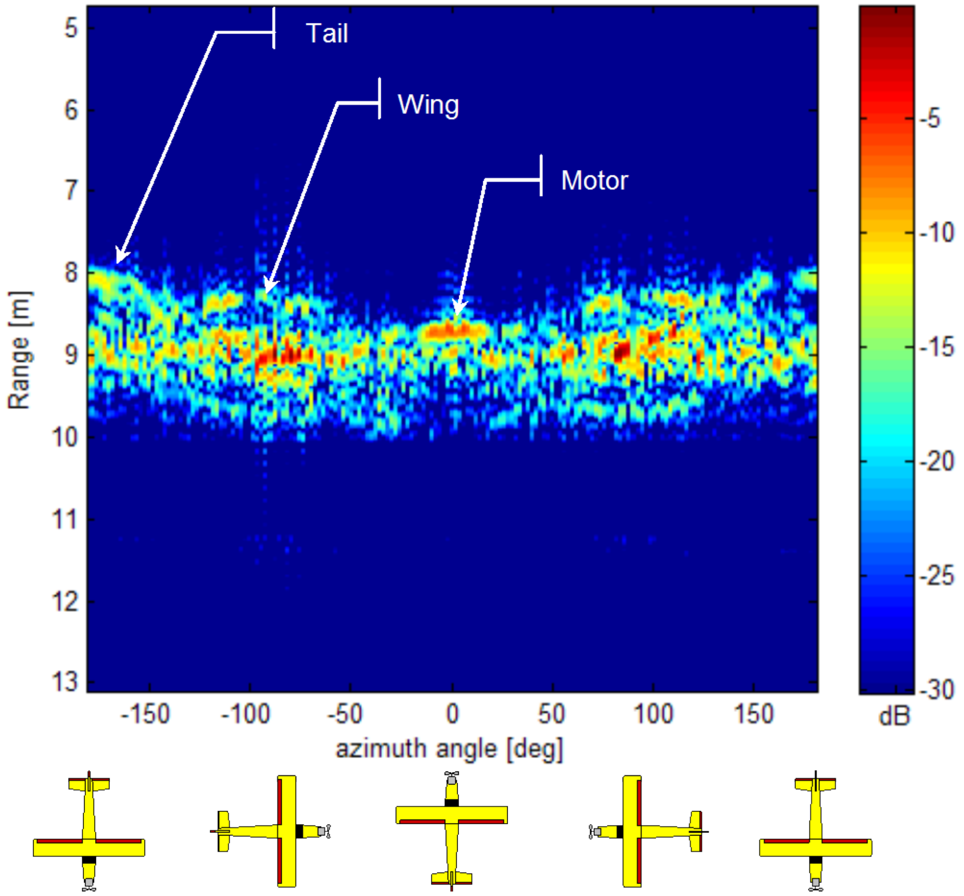

Figure 22 Range profiles X-band horizontal, aircraft at 0° elevation

In the X-band vertical polarisation case, the points with the most intense scattering seem to much more evenly distributed than in the horizontal case. But the scattering from the wings and tail can still easily be distinguished from the rest of the aircraft scatterers. Figure 23 is the image plot of range profiles from measurements with the wing and video camera removed.

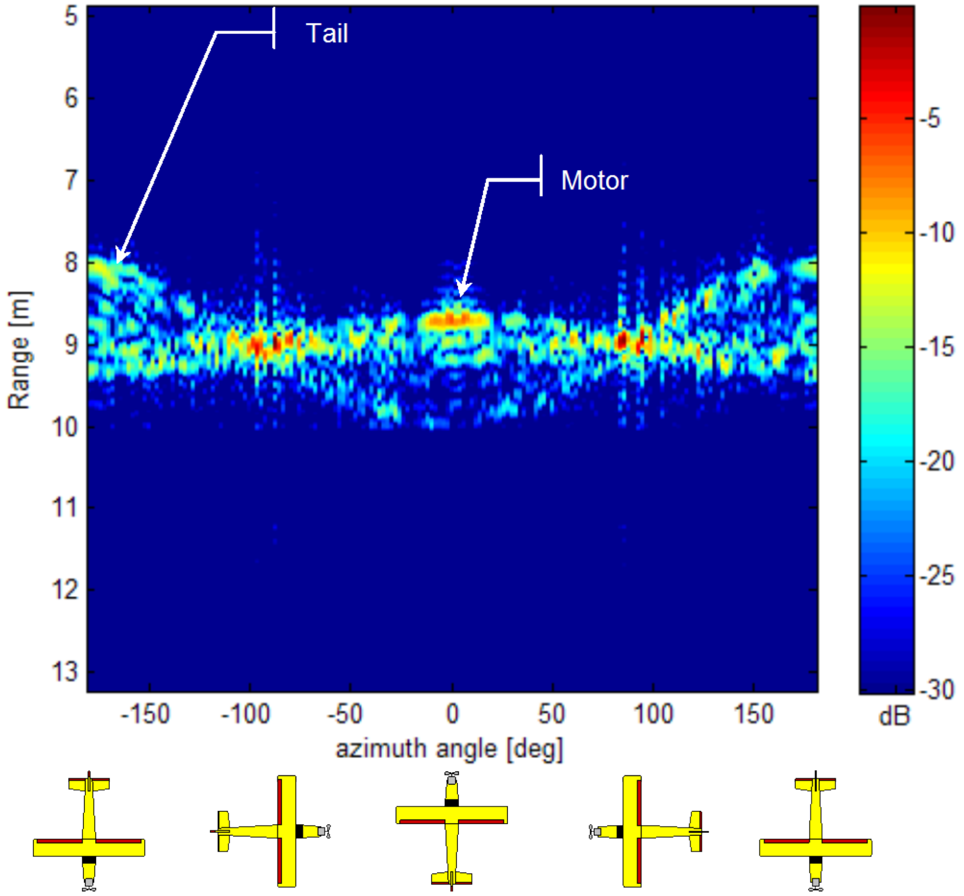

Figure 23 Range profiles X-band vertical with wing removed

Now the traces from the wing disappear and the scattering from the tail and the engine becomes more dominant and is easy to identify.

A new 360° RCS plot of the aircraft with the wing and video camera removed is in Figure 24 below.

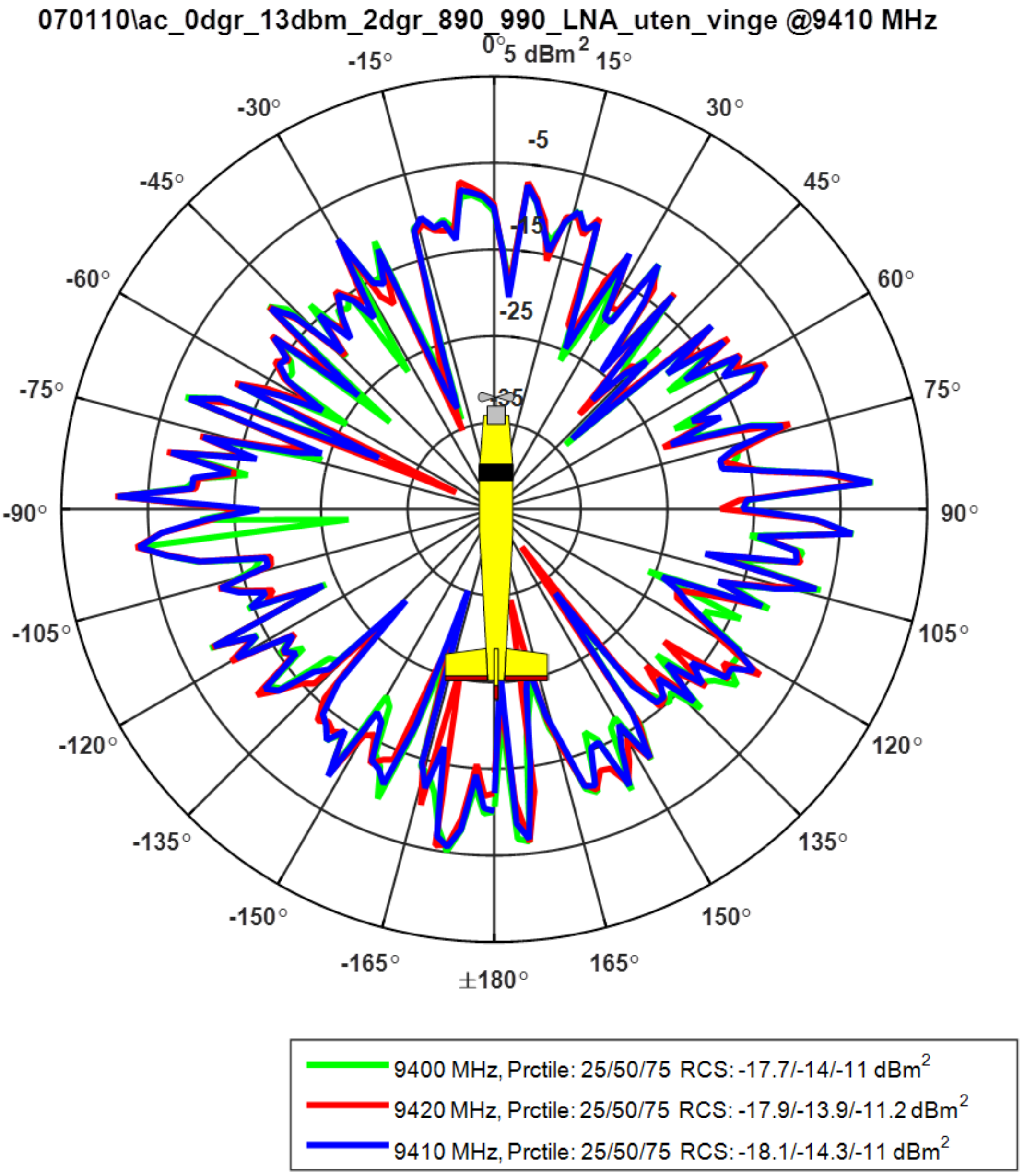

Figure 24 Aircraft X-band vertical RCS with wing and video camera removed

The 50 percentile RCS is now reduced from -12.9 dBm^2 of the original aircraft configuration to -14.3 dBm^2 (0.037m^2), which is less than the difference seen with X-band horizontal polarisation, but more in line with the reduction seen at S-band horizontal when the wing and video camera was removed.

As with the X-band vertical polarisation, the variation of RCS with elevation is somewhat larger than what was recorded in the S-band horizontal case, and again this may be attributed to the smaller wavelength making the RCS from the individual scatterers more sensitive to aspect angle. However, the maximum variation is small, only about 2.5 dB, and it is difficult to detect any clear trend in the data. Plots of aircraft RCS, X-band vertical polarisation, for various elevation angles are in Appendix F.

5 Summary

The average Radar Cross Section (RCS) of a model aircraft has been measured. The aircraft has been used as a radar test target to investigate into the performance of the NINA avian radar system at Smøla, but the RCS measurement method employed should also be applicable to other small radar targets. The measurements have been performed at 3050 MHz (S-band) and 9410 MHz (X-band). The aircraft has a wing span of about 2.1 m which requires it to be measured at a distance of at least 90 m at S-band and 277 m at X-band for it to be in the far-field. However, limitations in equipment sensitivity combined with heavy in-band interference, presumably from other radars, made it impossible to set-up the RCS measurements out-door at the SINTEF/NTNU facilities in Trondheim. Instead the measurements have been performed in an anechoic room in the antenna laboratory. This implies that the aircraft fuselage is in the near-field. But the main scatterer of the aircraft will not be the balsa and plywood fuselage itself, but rather the different mounted metallic parts inside and outside of the aircraft, e.g. battery pack, motor and video camera. Each individual component is small enough to be well within the far-field requirement, and even if the wiring aboard the aircraft complicates this simple model, the assumption is that in both the relevant radar bands the main reflecting structures of the aircraft are all in the far-field and will scatter energy more or less independent from each other. Test measurements made with parts of the aircraft removed, seemed to confirm this assumption. The amplitudes and the positions of the peaks and nulls in each of the individual scattering patterns will therefore be less sensitive to the measurement range, and there will only be a slight shift of the lobes and nulls in the composite pattern compared to the true far-field pattern. But since the purpose of these RCS measurements was not to make an accurate mapping of the model aircraft RCS, but rather to find and compare its average RCS to a relevant bird size target to be able to assess test data recorded when the aircraft is used for radar performance testing, measurements in the anechoic room with the aircraft fuselage in the near-field, but with the individual dominant reflectors in the far-field, were considered sufficient.

Since the RCS levels to be measured were very much smaller than the response from the measurement support tower and the anechoic room itself, the empty room response was first measured and then coherently subtracted from the total response from the room with the aircraft in place, the difference being just the response from the aircraft. This method has two major error sources. First there may be multipath reflections, e.g. from the aircraft via the measurement tower, that will be present only when the aircraft is installed in the room and therefore not removed when the empty room response is subtracted from the measurements. Secondly the measurement tower in the anechoic room has moving parts that can cause a randomly varying return that cannot be removed by simply subtracting two consecutive measurements. This latter effect was by far worst at X-band, and the response from the measurement tower had to be gated out in the time domain before RCS estimation could be made. But apart from strong returns from moving parts at X-band, the method of subtracting the empty room response worked reasonably well and the errors introduced appeared to be relatively small.

The measurements have been calibrated and the system transfer function verified by the use of 5 simple shape calibration objects of different size and RCS. The results from the calibration indicate that the accuracy of the RCS measurements obtained with this method is within 2 dB.

With the constraints of the measurement method, the 50 percentile RCS of the model aircraft, over 360°, with the current configuration, has been found to be -11 dBm^2 (0.079 m^2) for S-band horizontal polarisation, and -13.8 dBm^2 (0.042 m^2) and -12.9 dBm^2 (0.051m^2) for X-band

horizontal and vertical polarisation respectively. These results are all with the aircraft at 0° pitch, but the RCS showed little variation as the aircraft pitch was varied from -20° to 20°.

6 References

[1] Barton David K: Modern Radar System Analysis, Artech house 1988
[2] IEEE Standard Dictionary of Electrical and Electronic Terms, IEEE Std 100-1996
[3] Knott Eugene F. et. Al: Radar Cross Section, 2nd ed. Artech House 1993
[4] Skolnik Merrill I: Introduction to radar systems, 2nd ed. McGraw-Hill, 1980
[5] Hewlett Packard: Network Analyzer HP8719C/HP8720C/HP8722C, technical data

7 Document revision history

Rev. No.	Description	Date	Signature
1.0	Initial release	18.03.10	Yngve Steinheim
1.1	With minor typographical corrections	16.06.10	Yngve Steinheim
1.2	"Far-field" replaced by "near-field" in first sentence in paragraph 4.2.1.1 describing the X-band calibration method.	16.08.11	Yngve Steinheim
2.0	Updated with RCS-data described by percentiles. This revision has been done after the author left SINTEF.	22.09.16	Yngve Steinheim

Appendix A Model aircraft configuration

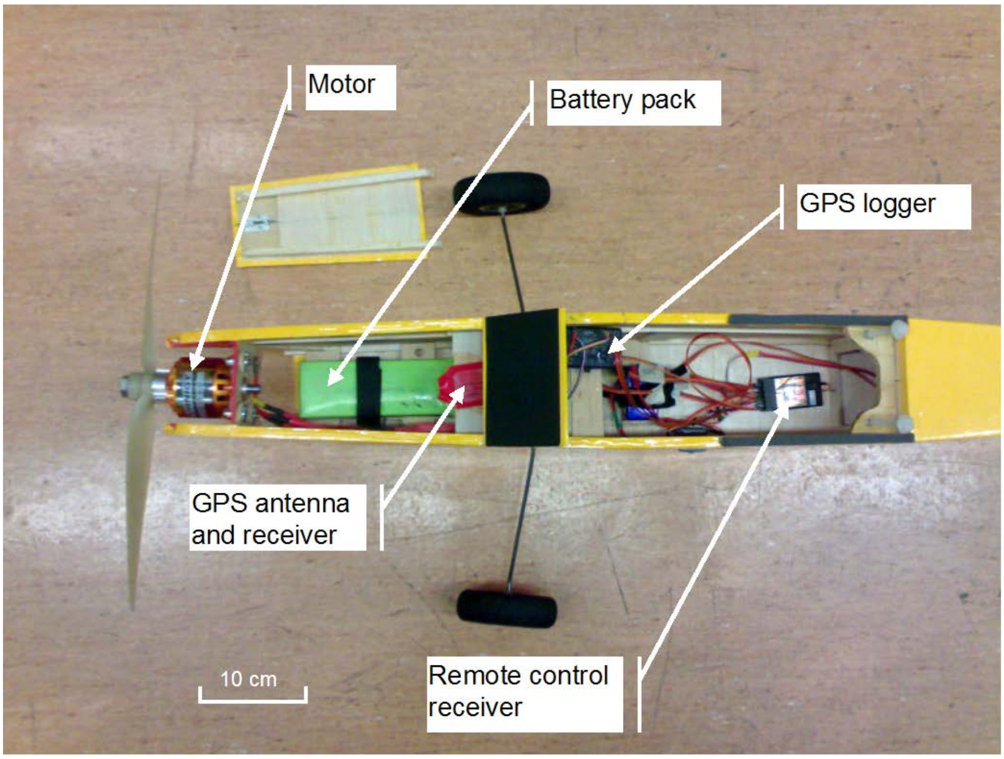

Figure A- 1 Model aircraft interior

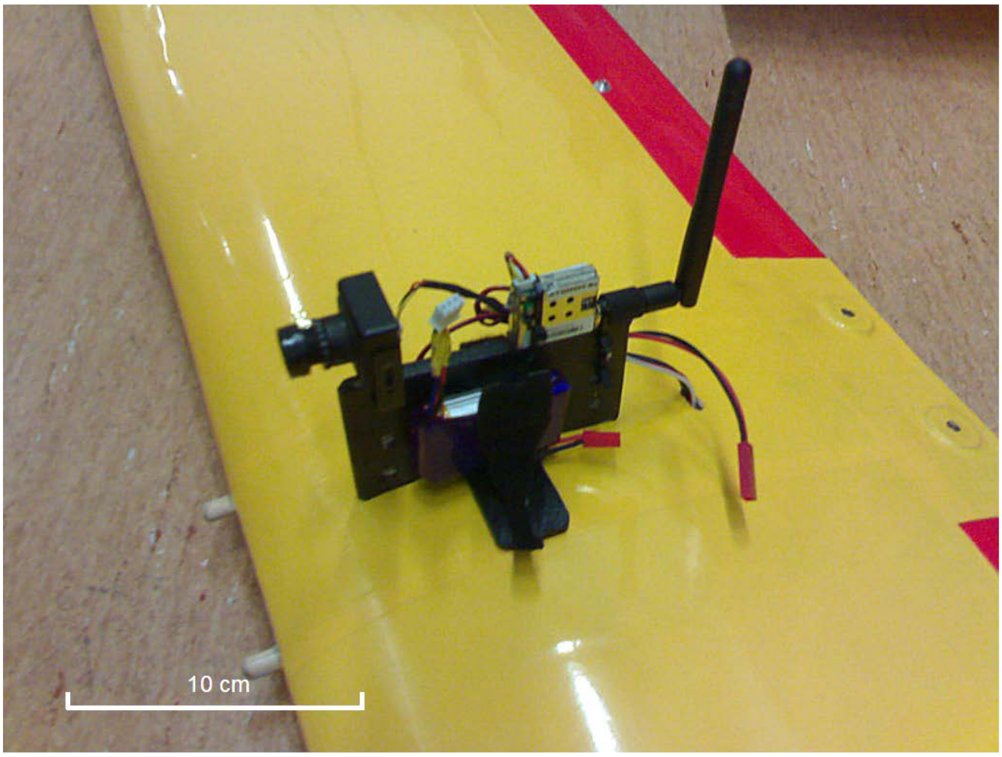

Figure A- 2 Video camera, 500 mW video transmitter (2.4 GHz) mounted on top of the wing

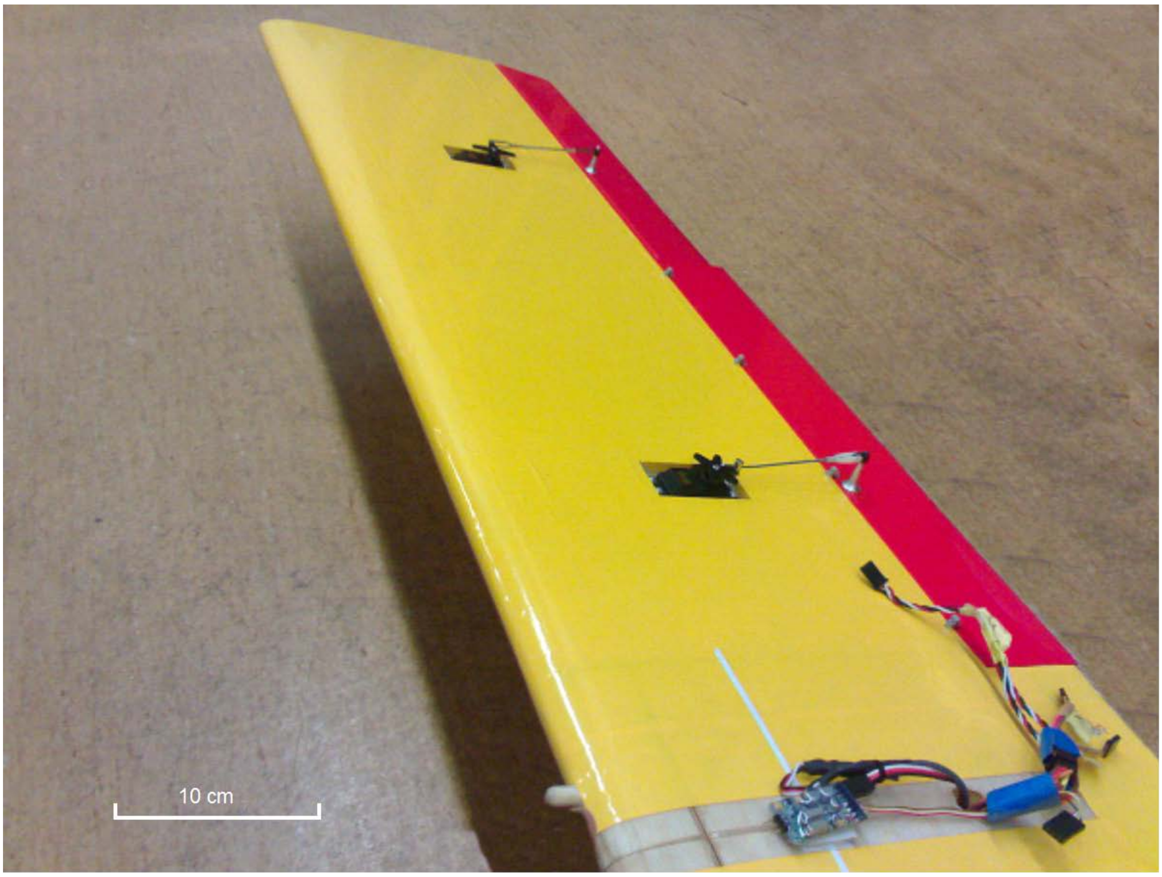

Figure A- 3 Underside of wing with connectors for flaps and aileron servos and video camera

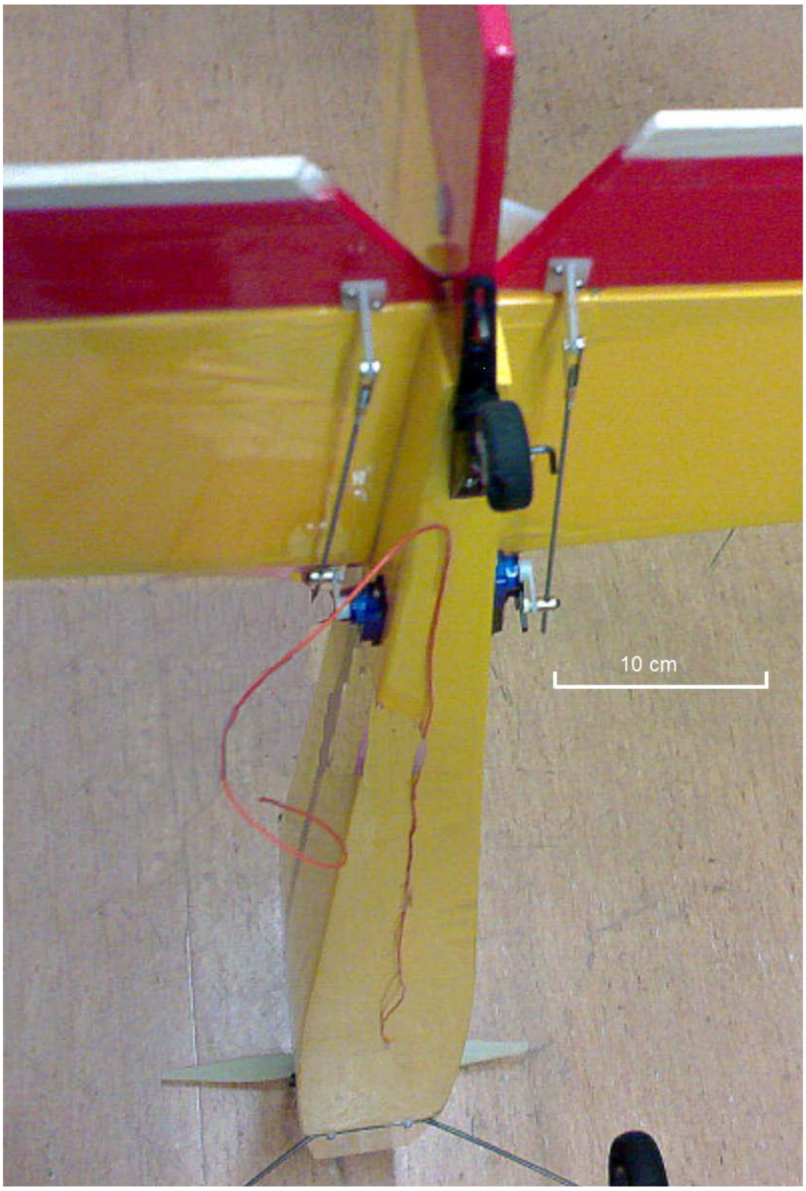

Figure A- 4 Underside of fuselage with antenna for remote control, elevators and tail wheel

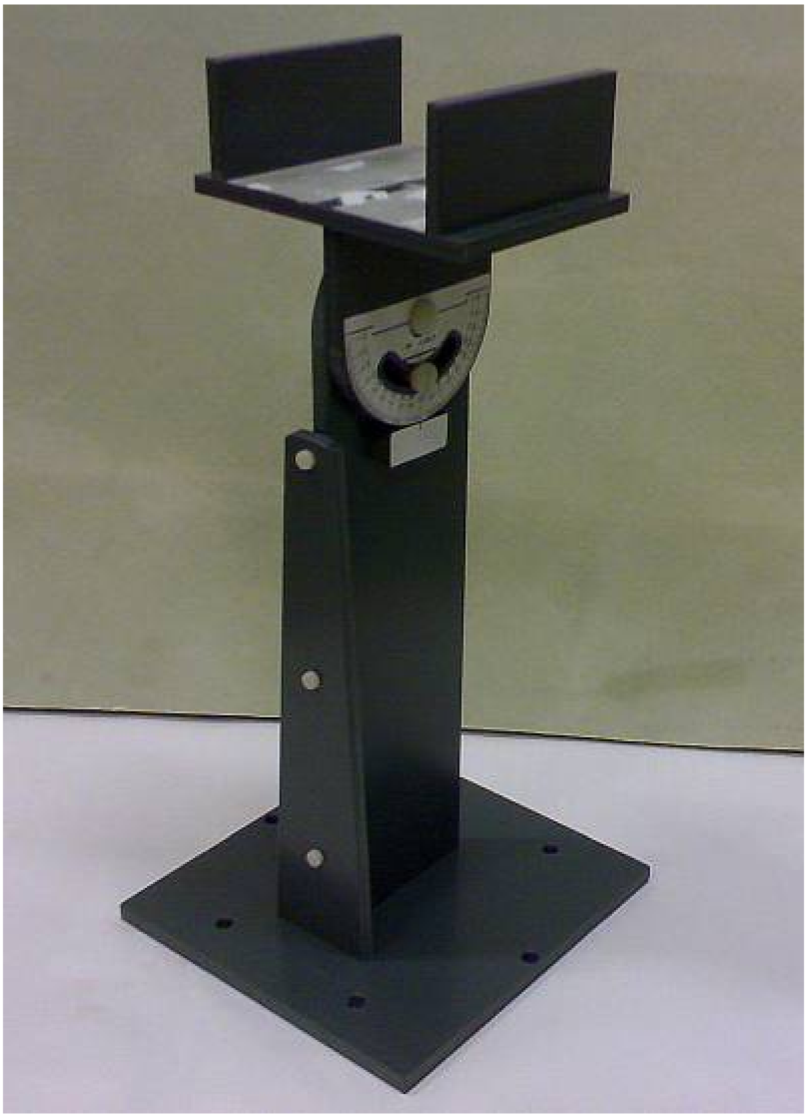

Figure A- 5 PVC aircraft mounting bracket with pitch adjustment

Appendix B Equipment list

Eqipment	Type/PN
Network Analyzer	Hewlett-Packard 8720C
Pre-amplifier	HP83017A 0.5-26.5 GHz
Rx cables	Suhner Sucoflex 104p (1m)<br>Suhner RG-58 C/U (0.75m)
Tx cable	Huber+Suhner Enviroflex 400 (2m)
Rx/Tx antennas	EMCO Double ridge horn antenna, model 3115, 1-18 GHz
Motion Controller	Newport MM4005
PC with Windows Vista and GPIB interface	
MATLAB	Ver 2009a, v7.8.0.347 with instrument control toolbox.<br>MATLAB script for antenna measurements: "AntennaPattern_sintef"<br>(Script developed by Marius Ubostad/NTNU, modified by Jagath Gamage/Yngve Steinheim)

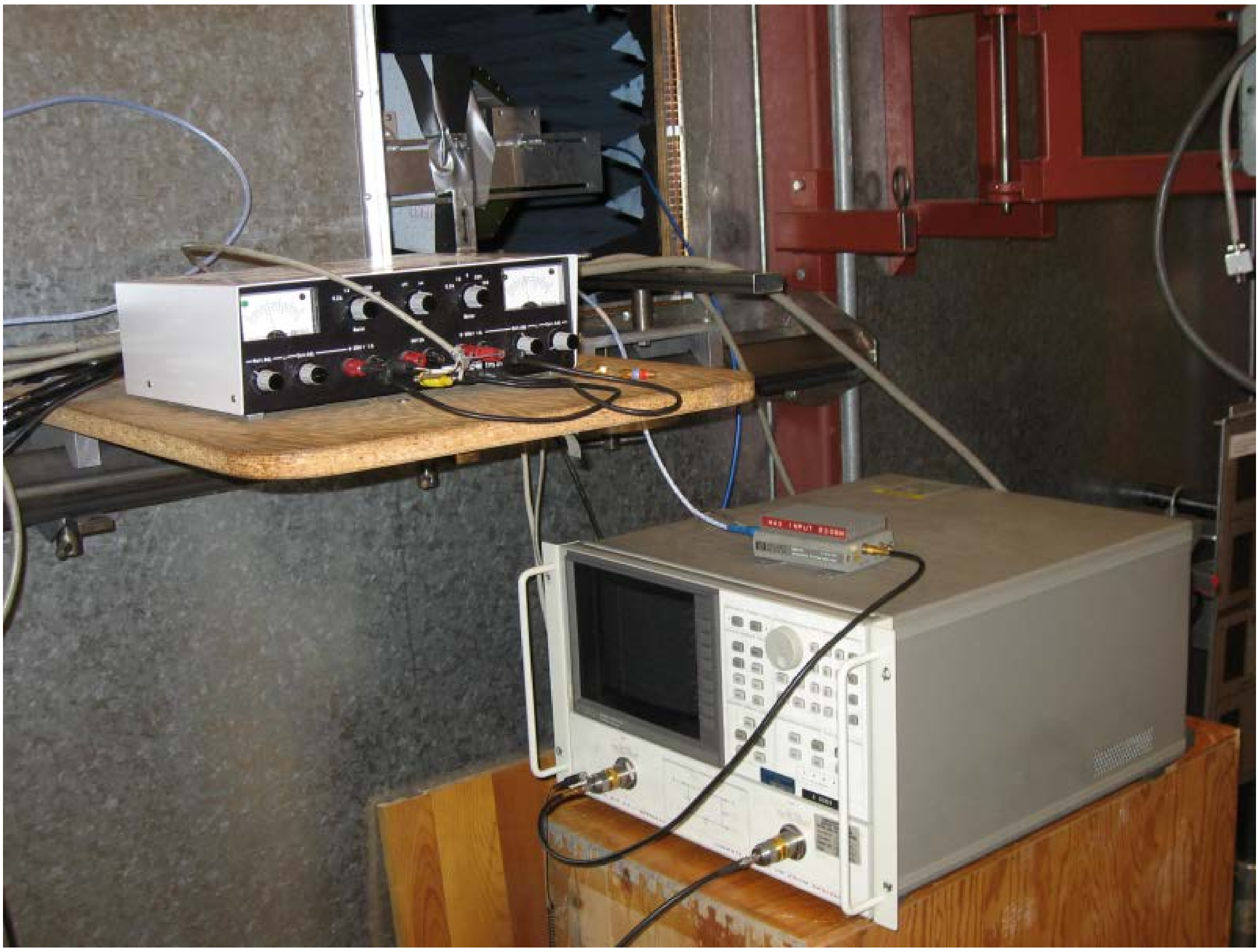

Figure B- 1 HP 8720C network analyzer and HP83017A pre-amplifier

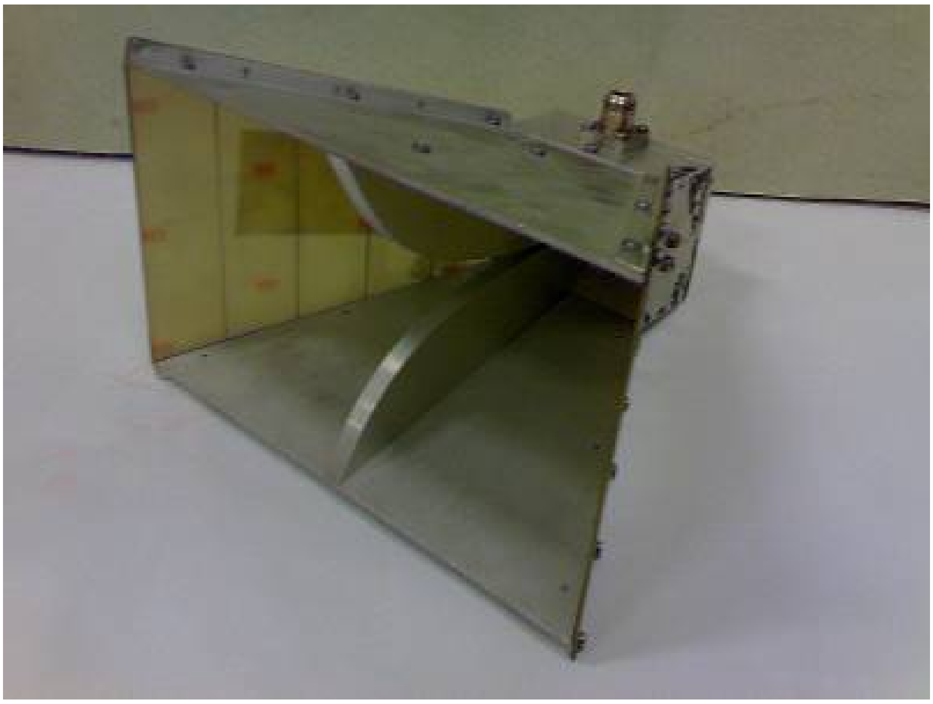

Figure B- 2 EMCO double ridge guide horn antenna, model 3115

Appendix C Link budget

Link budget S-band RCS measurements anechoic room

Frequency: 3050 MHz

	Link budget	Comment
Output power [dBm]	10	Network analyzer output, 3050 MHz
Tx cable loss [dB]	2	
Sender antenna gain [dB]	8,6	at 3GHz
Radar Cross Section [dBsm]	-20	
Range [m]	6,1	
Receiver antenna gain [dB]	8,6	at 3GHz
Pre-amp gain [dB]	36	HP 83017A @3GHz
Rx cable loss [dB]	2	
Received power [dBm]	**-45,33**	

Link budget X-band RCS measurements anechoic room

Frequency: 9410 MHz

	Link budget	Comment
Output power [dBm]	10	Network analyzer output, 9410 MHz
Tx cable loss [dB]	2	
Sender antenna gain [dB]	11,6	at 9,5GHz
Radar Cross Section [dBsm]	-20	
Range [m]	6,3	
Receiver antenna gain [dB]	11,6	at 9,5GHz
Pre-amp gain [dB]	36	HP83017A @9GHz
Receiver cable loss [dB]	2	
Received power [dBm]	**-49,68**	

Appendix D S-band RCS, horizontal polarisation

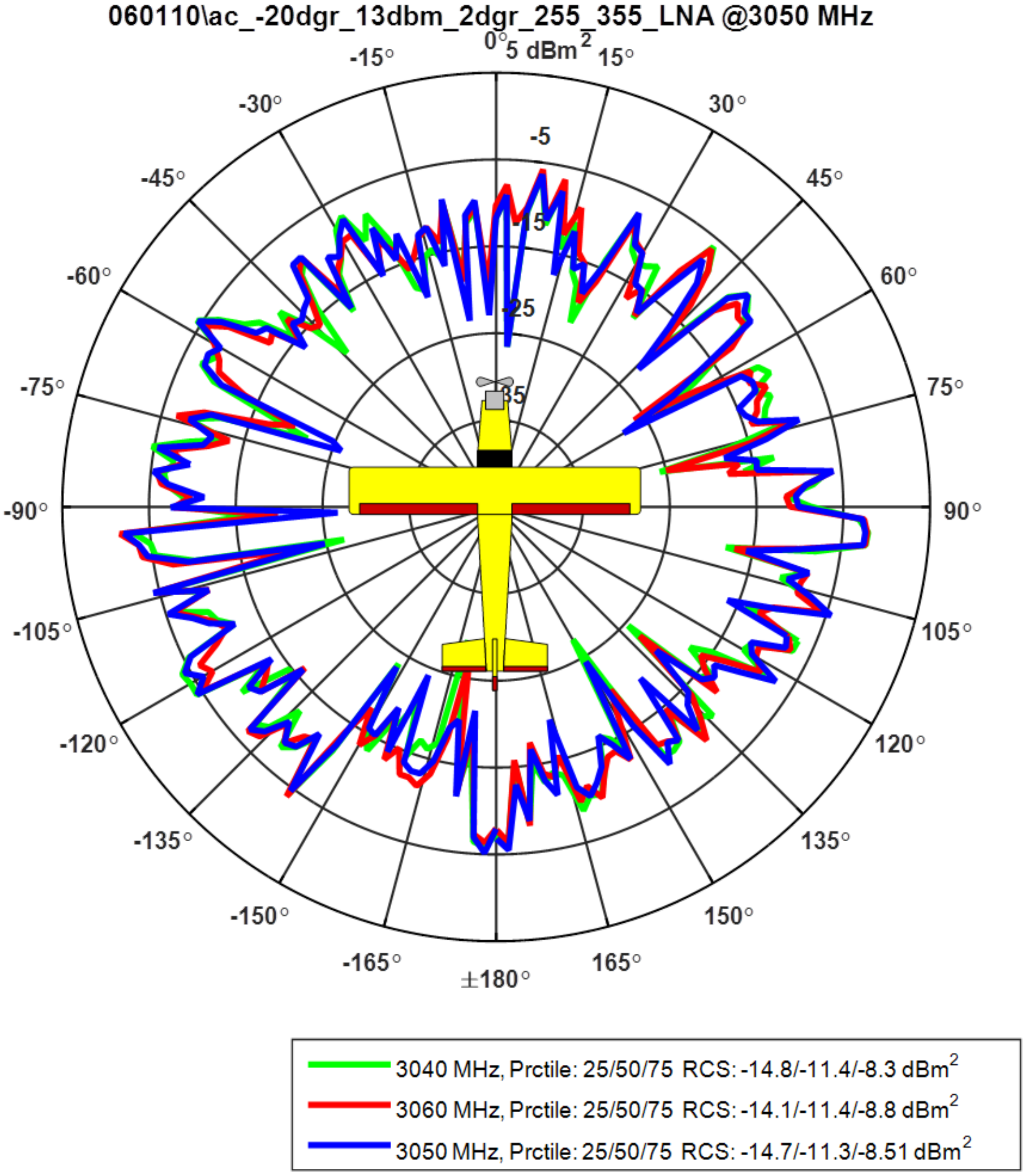

Figure D- 1 S-band RCS, horizontal polarisation, -20° elevation

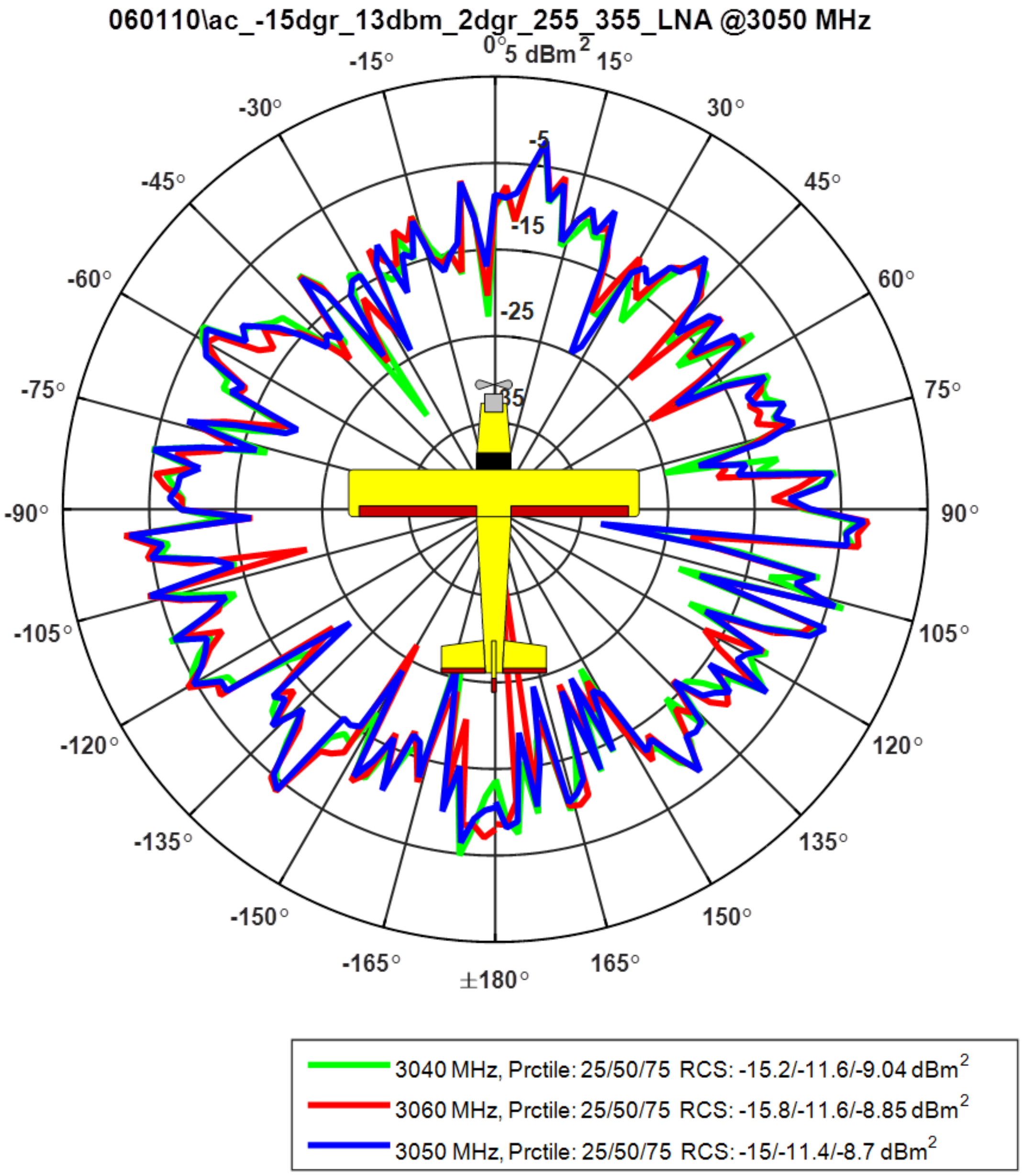

Figure D- 2 S-band RCS, horizontal polarisation, -15° elevation

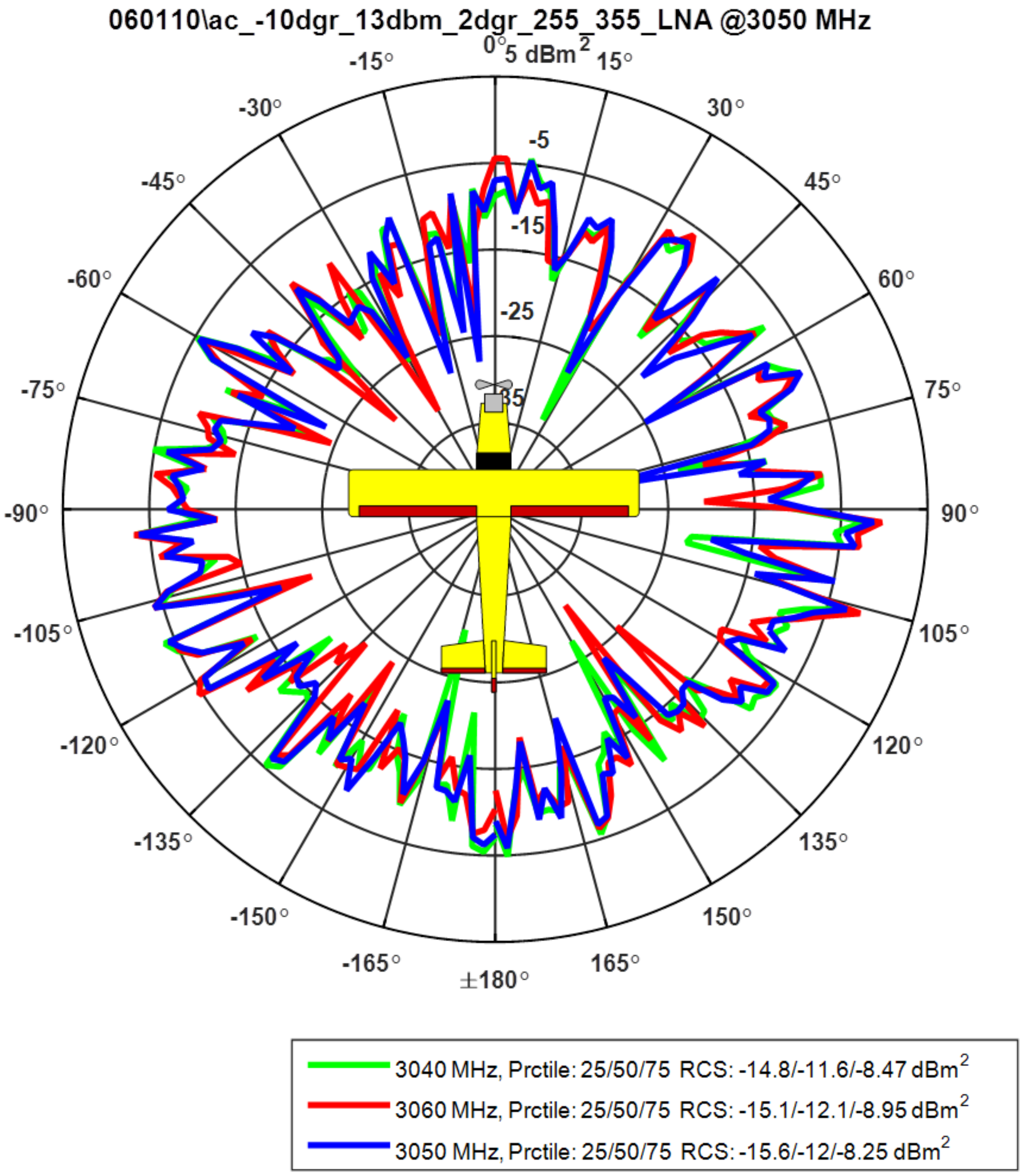

Figure D- 3 S-band RCS, horizontal polarisation, -10° elevation

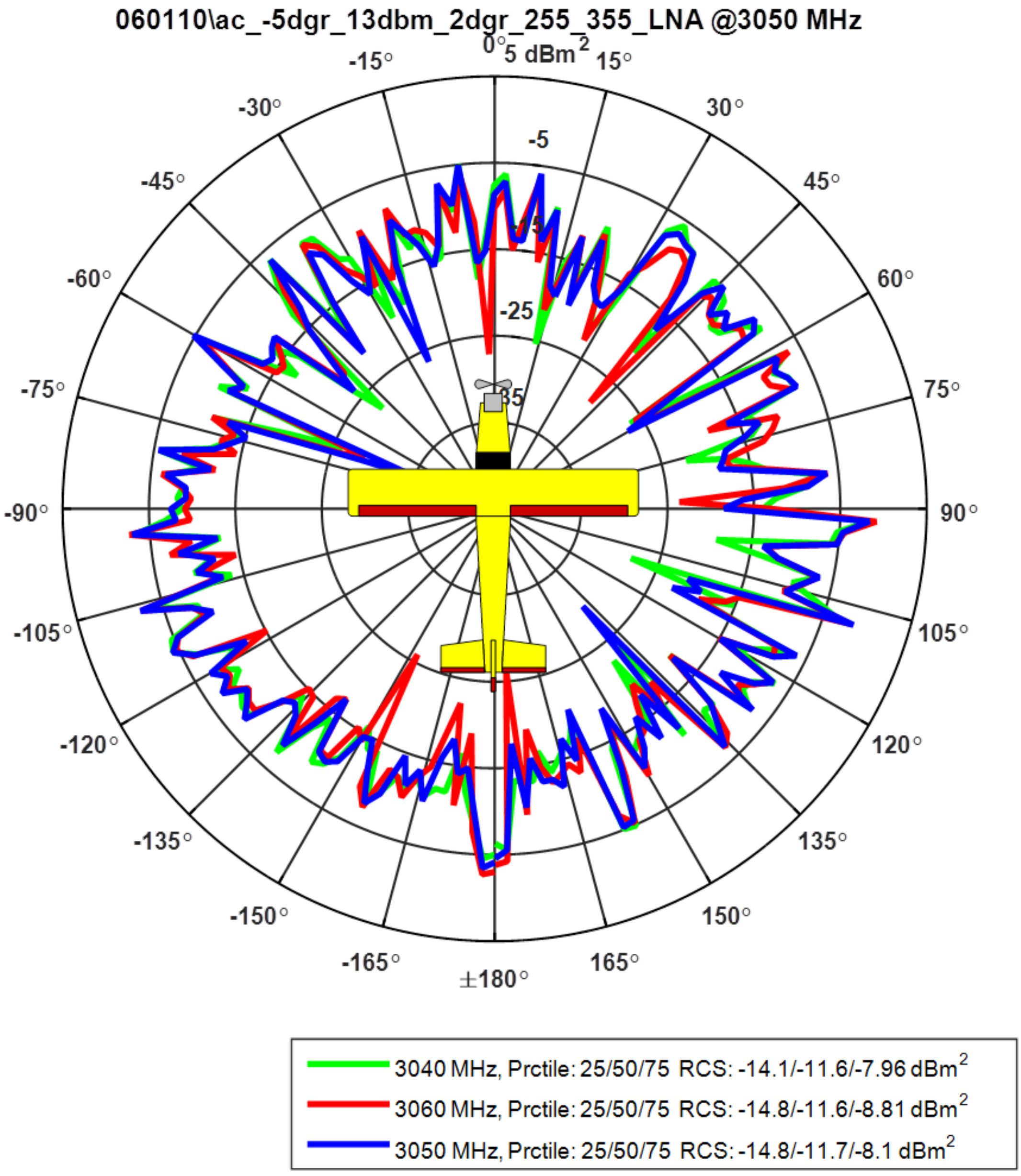

Figure D- 4 S-band RCS, horizontal polarisation, -5° elevation

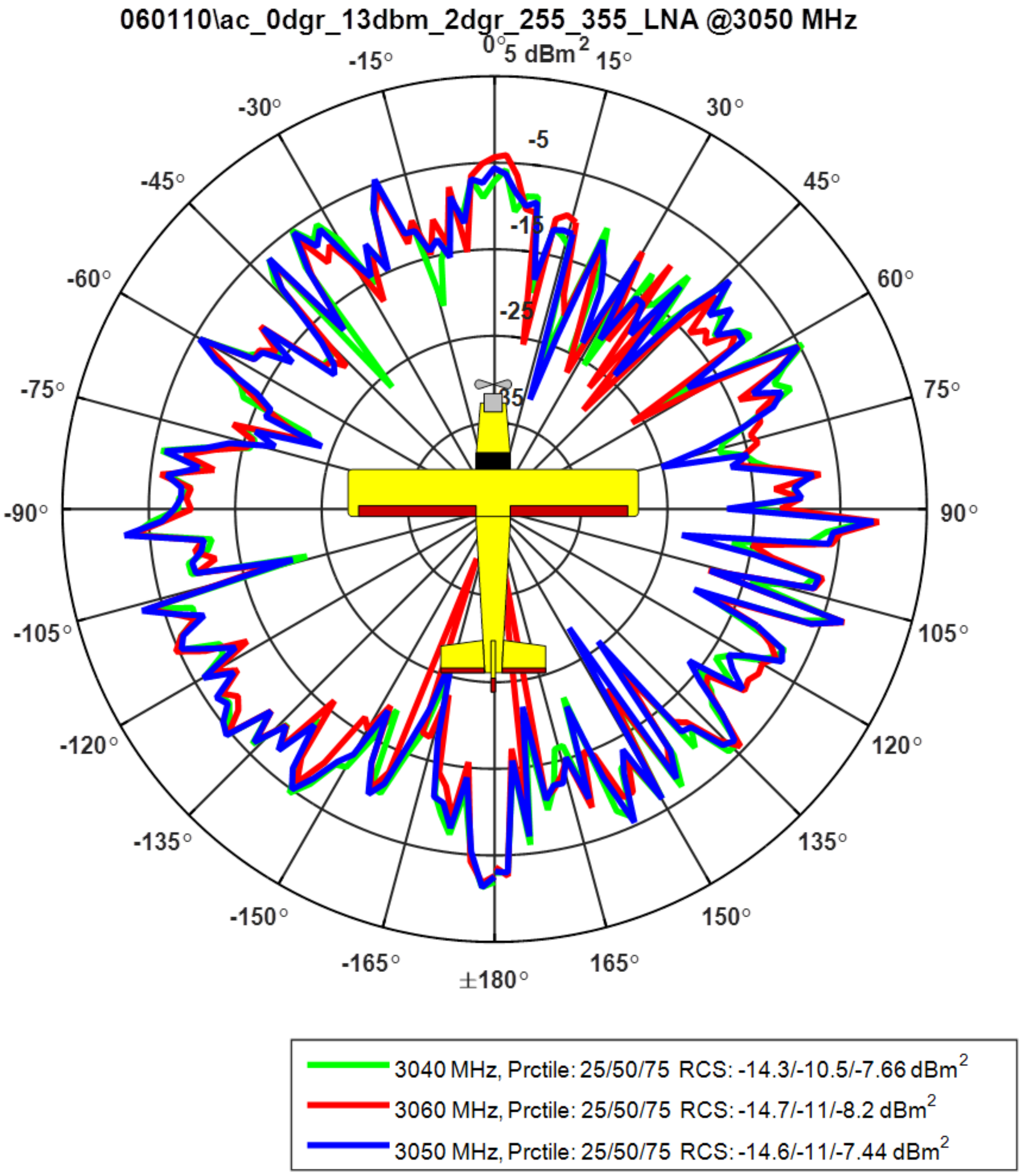

Figure D- 5 S-band RCS, horizontal polarisation, 0° elevation

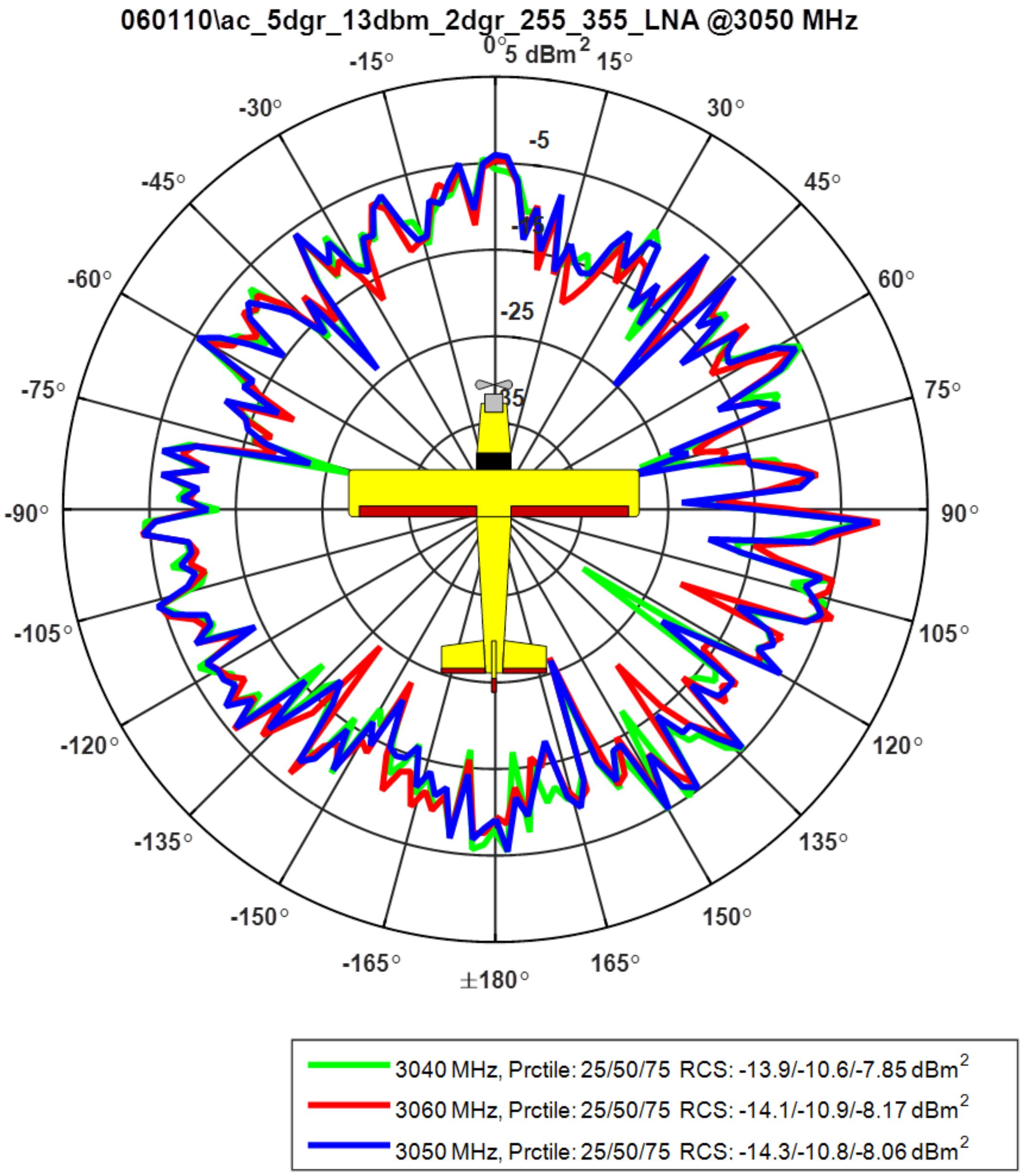

Figure D- 6 S-band RCS, horizontal polarisation, 5° elevation

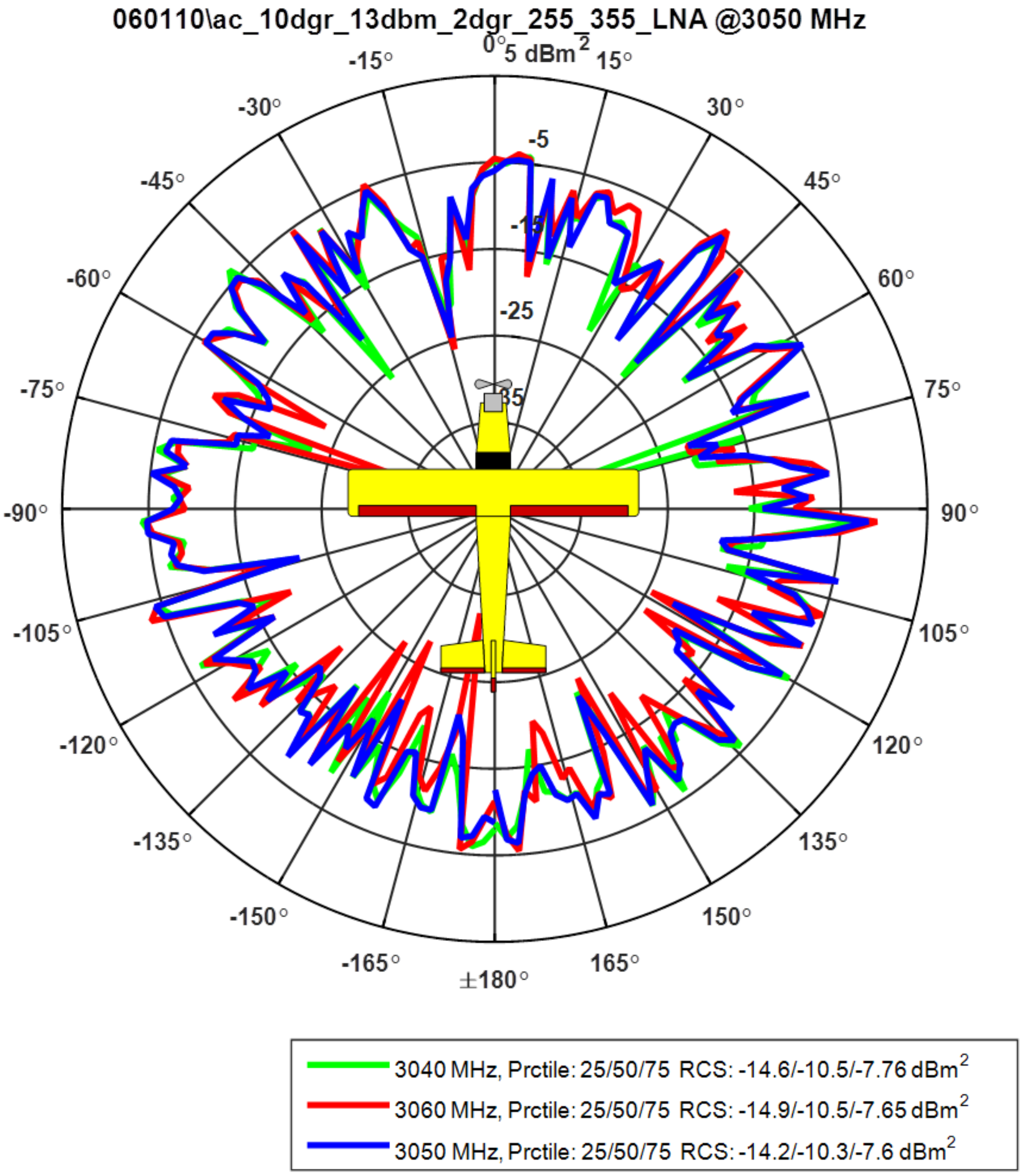

Figure D- 7 S-band RCS, horizontal polarisation, 10° elevation

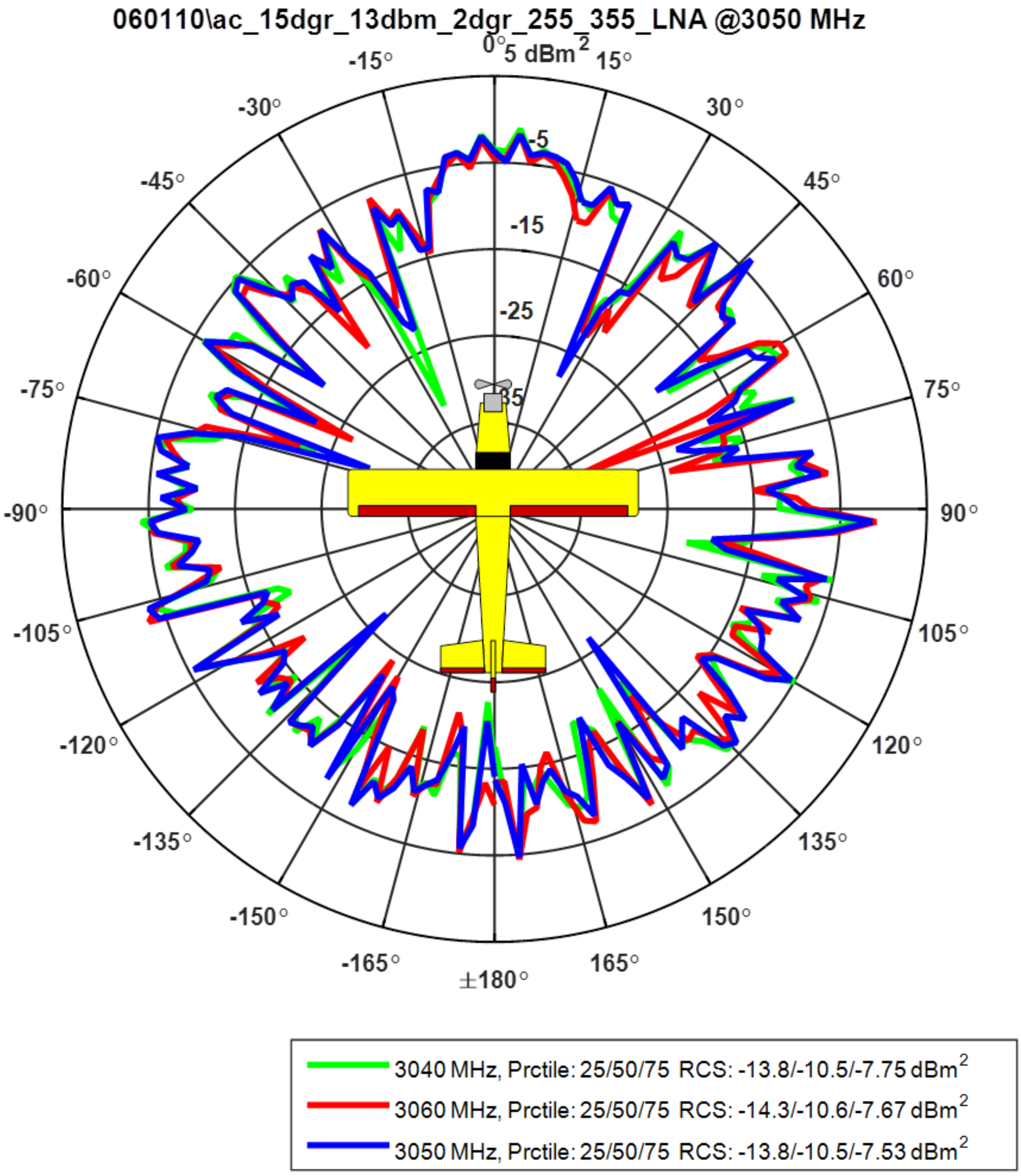

Figure D- 8 S-band RCS, horizontal polarisation, 15° elevation

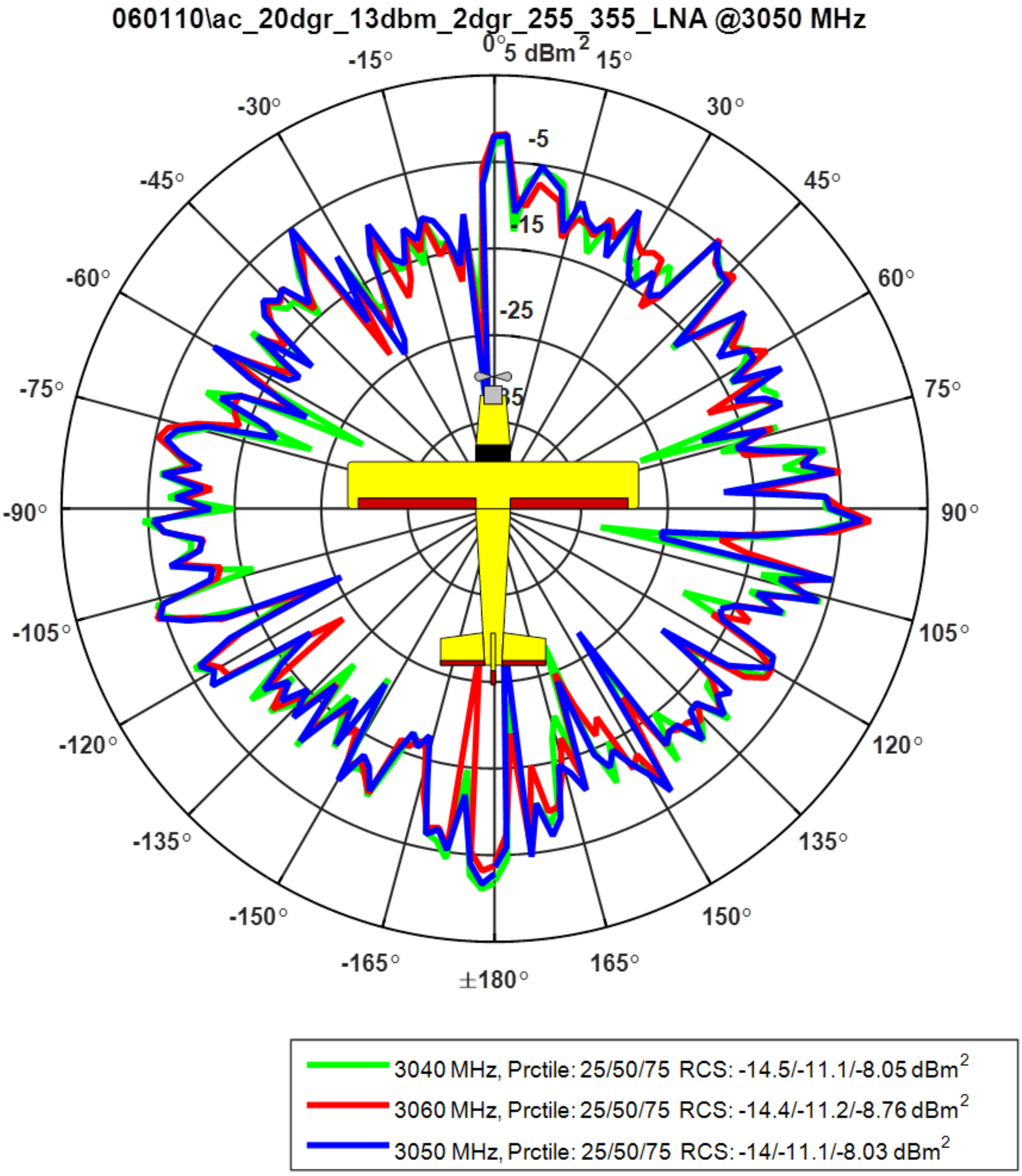

Figure D- 9 S-band RCS, horizontal polarisation, 20° elevation

Appendix E X-band RCS, horizontal polarisation

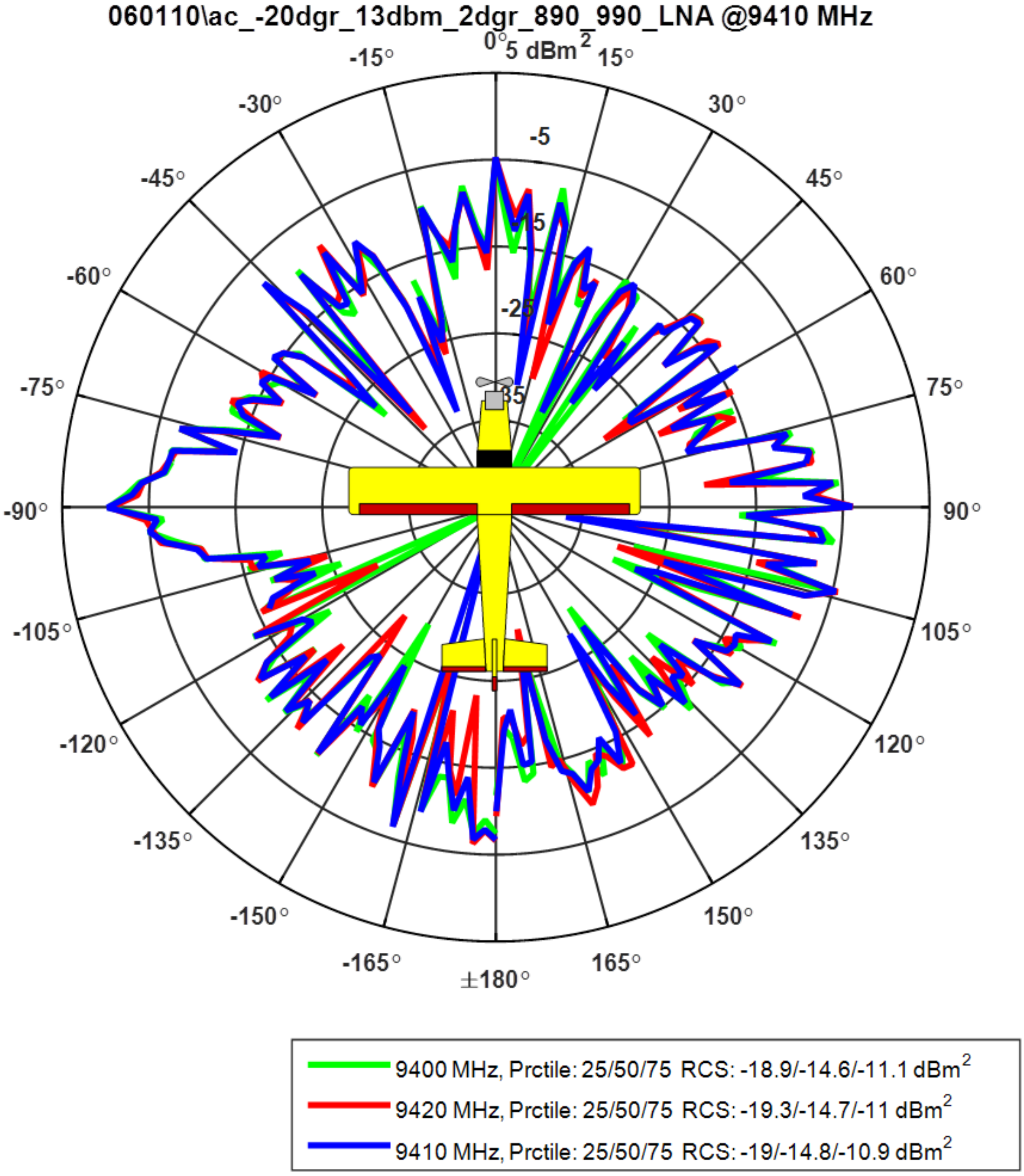

Figure E- 1 X-band RCS, horizontal polarisation, -20° elevation

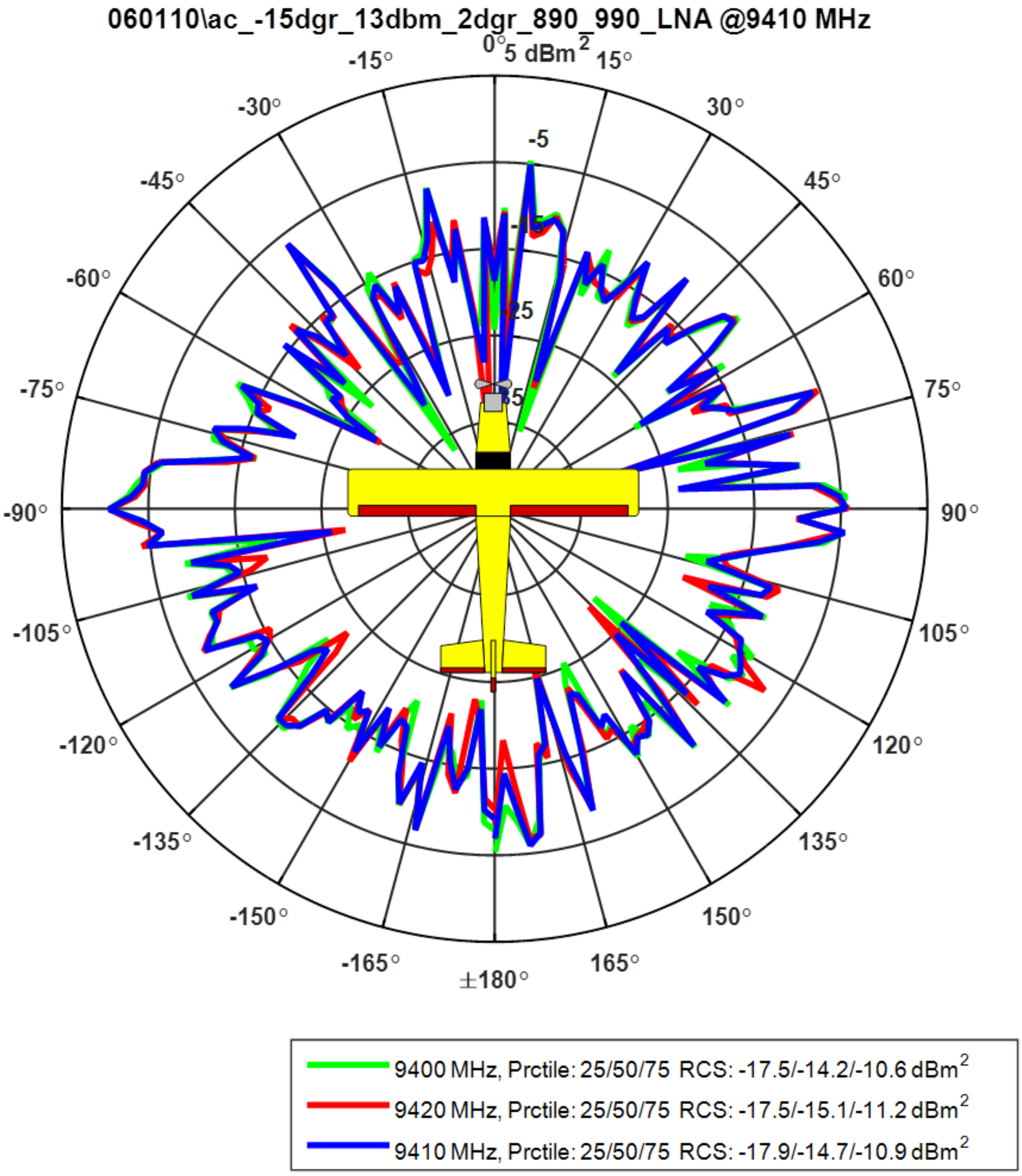

Figure E- 2 X-band RCS, horizontal polarisation, -15° elevation

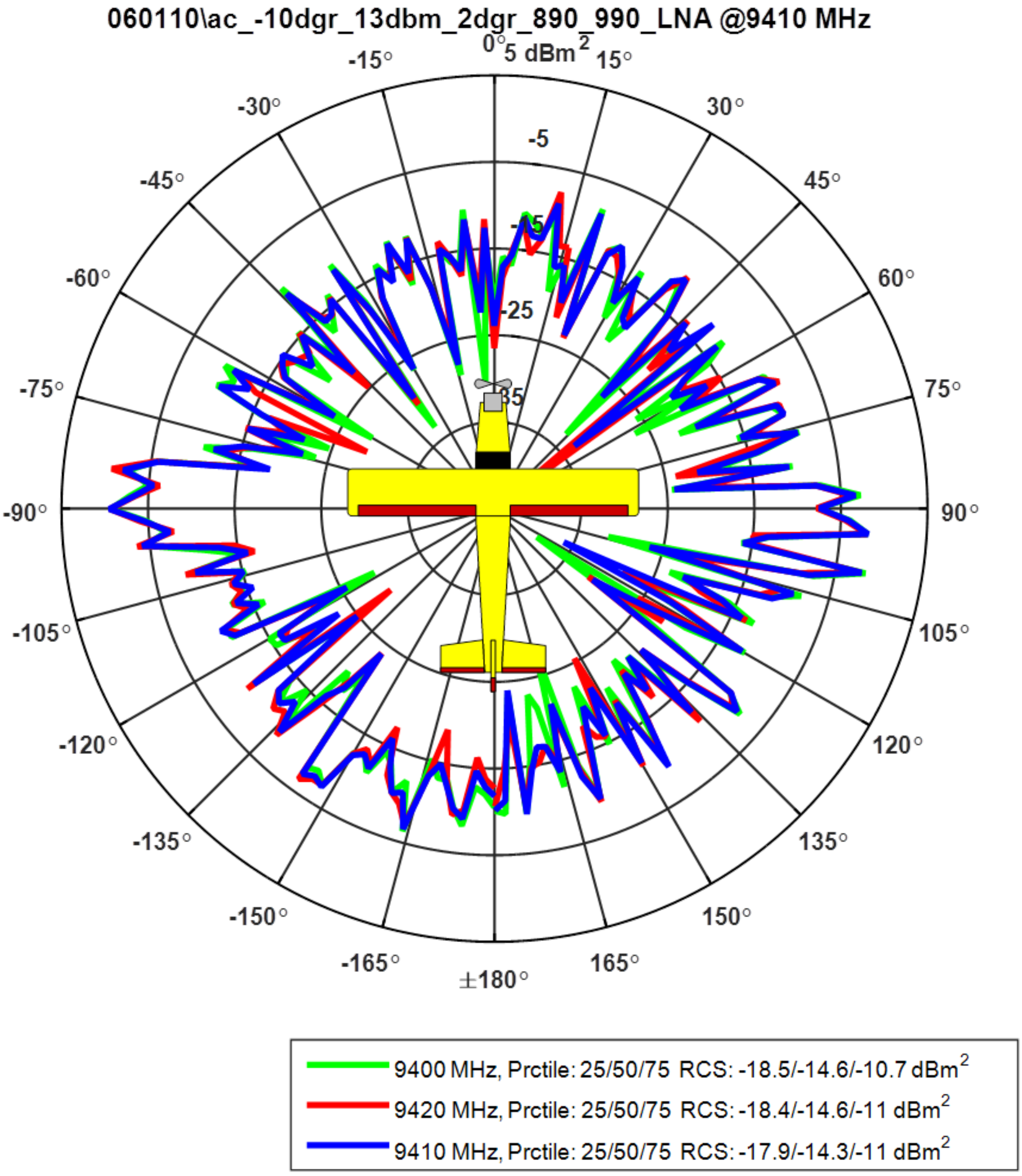

Figure E- 3 X-band RCS, horizontal polarisation, -10° elevation

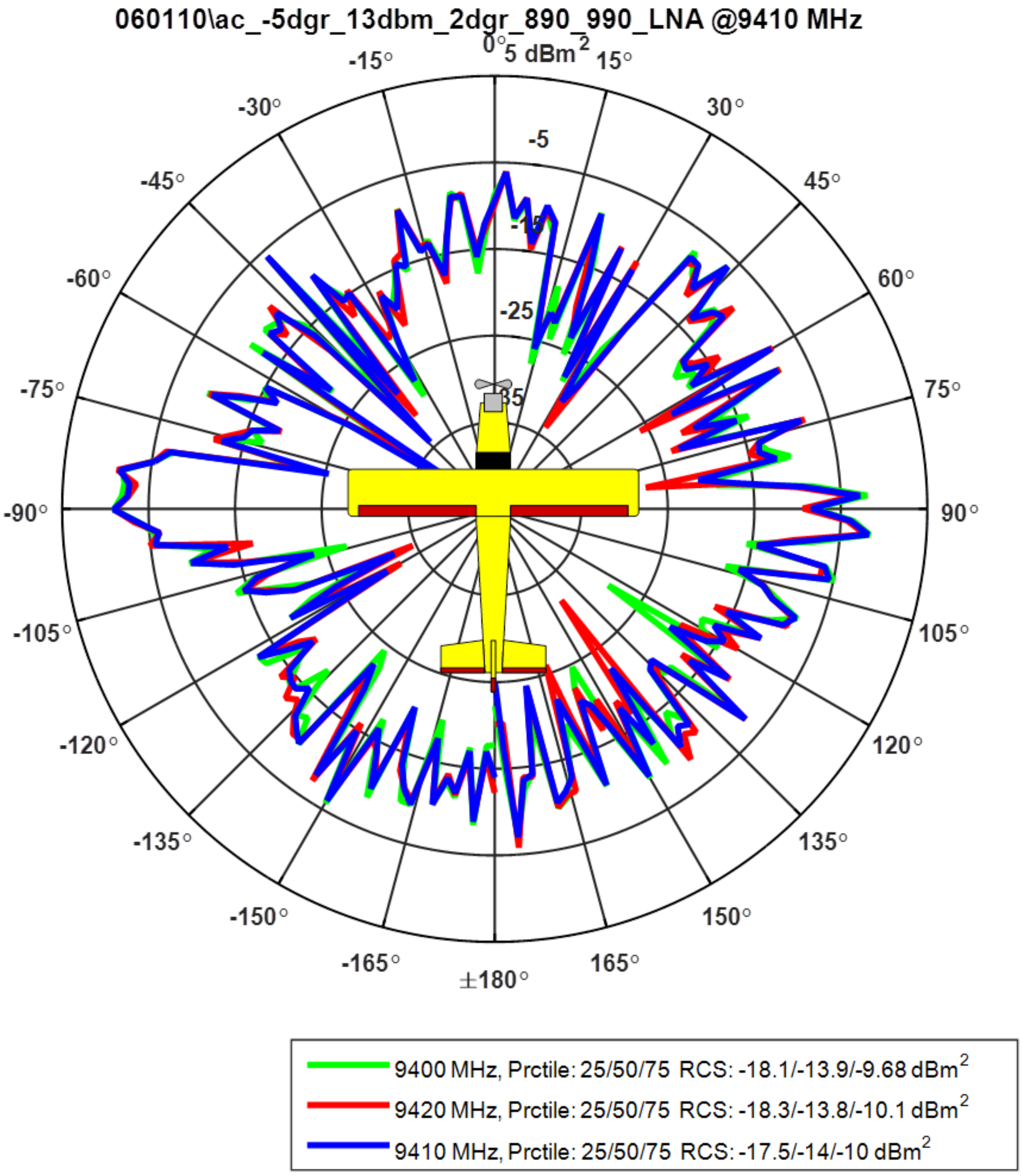

Figure E- 4 X-band RCS, horizontal polarisation, -5° elevation

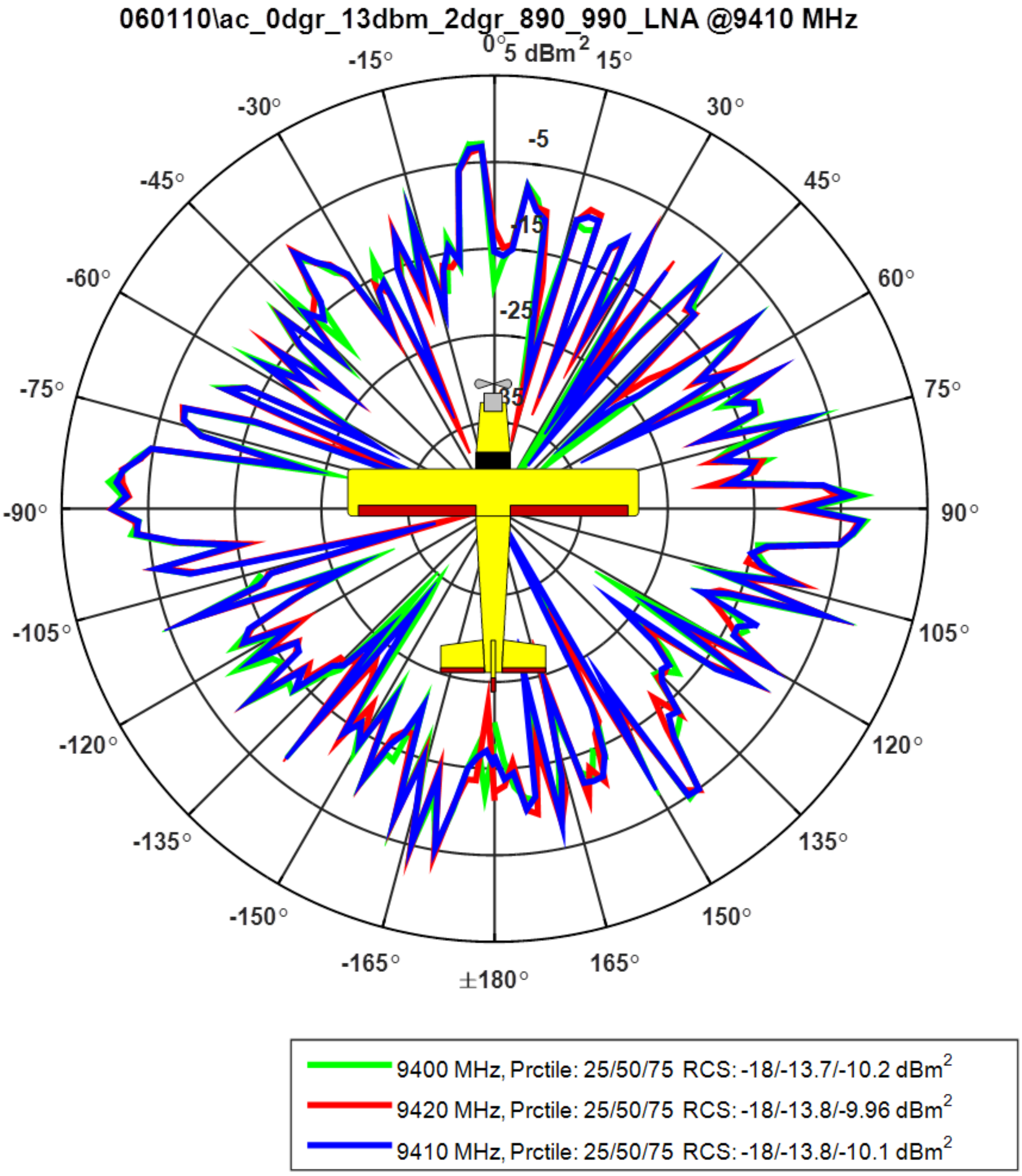

Figure E- 5 X-band RCS, horizontal polarisation, 0° elevation

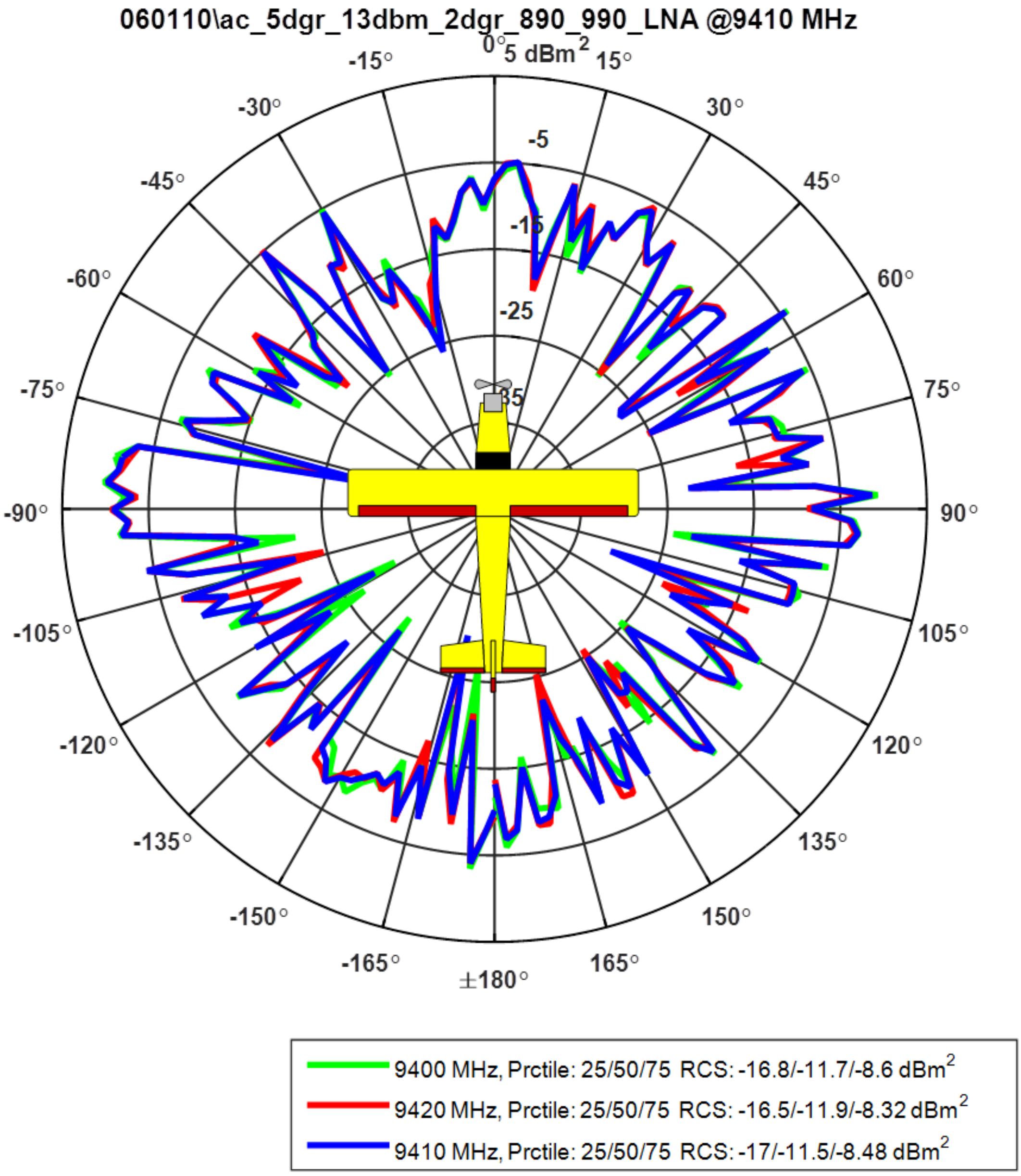

Figure E- 6 X-band RCS, horizontal polarisation, 5° elevation

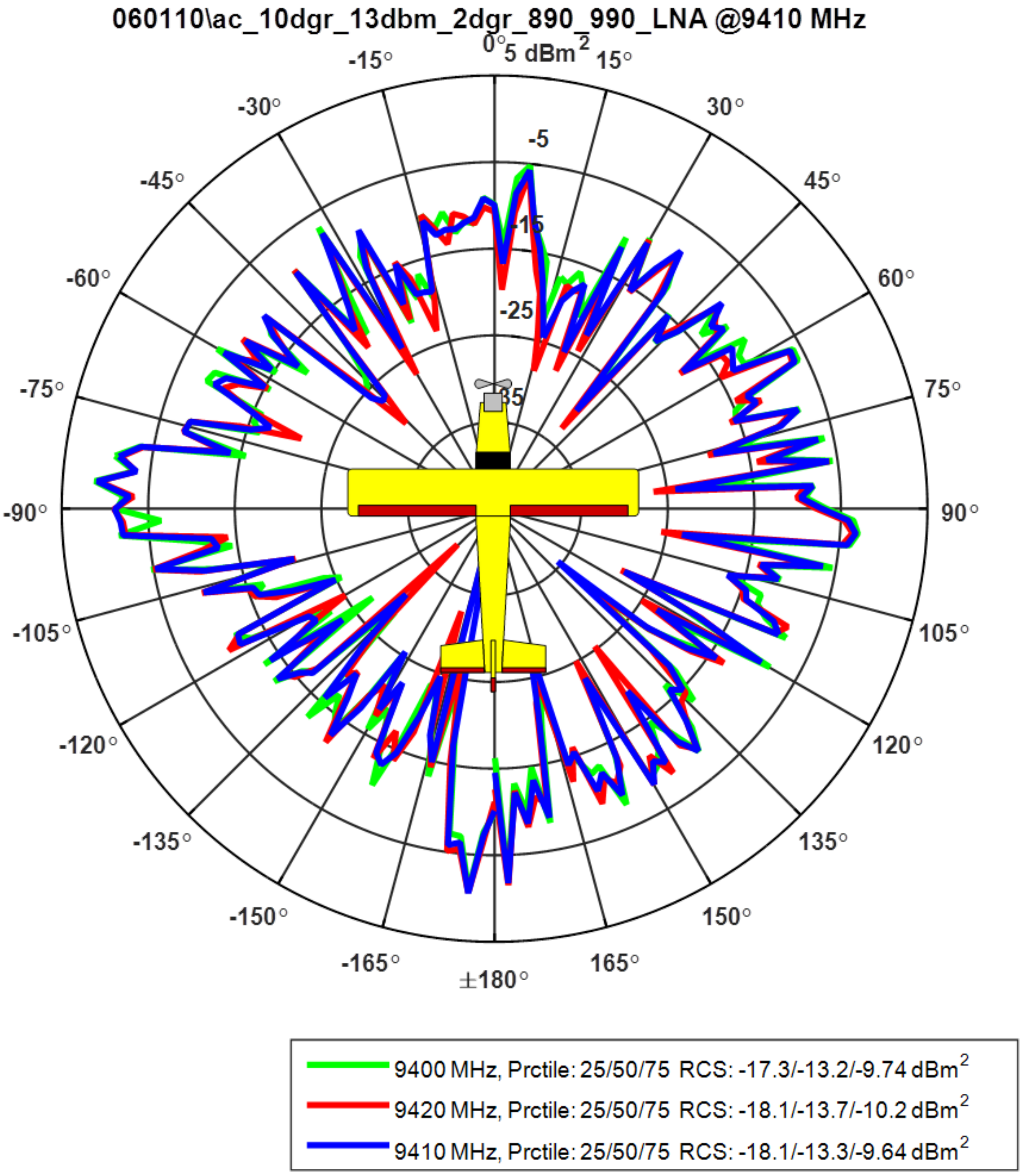

Figure E- 7 X-band RCS, horizontal polarisation, 10° elevation

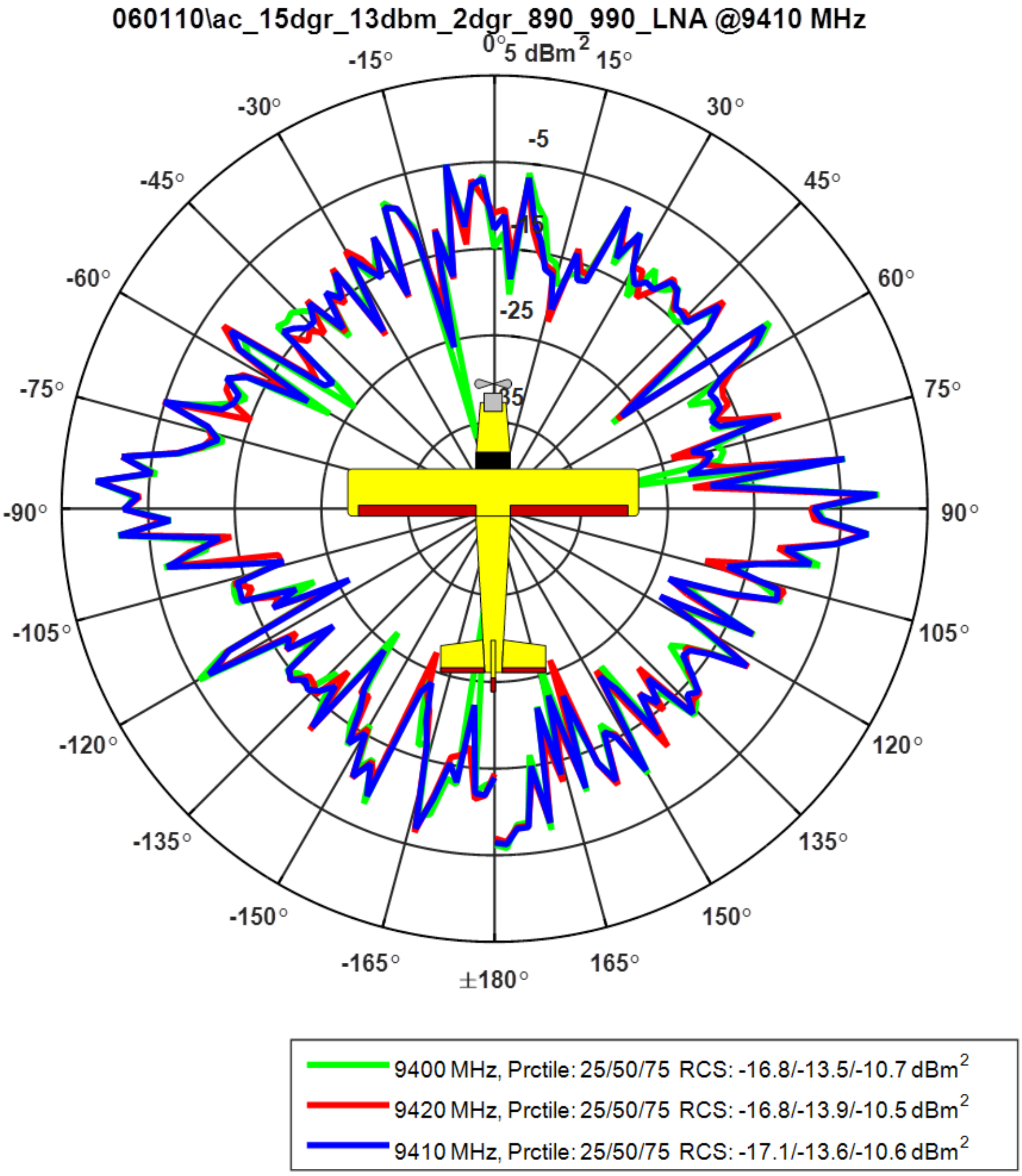

Figure E- 8 X-band RCS, horizontal polarisation, 15° elevation

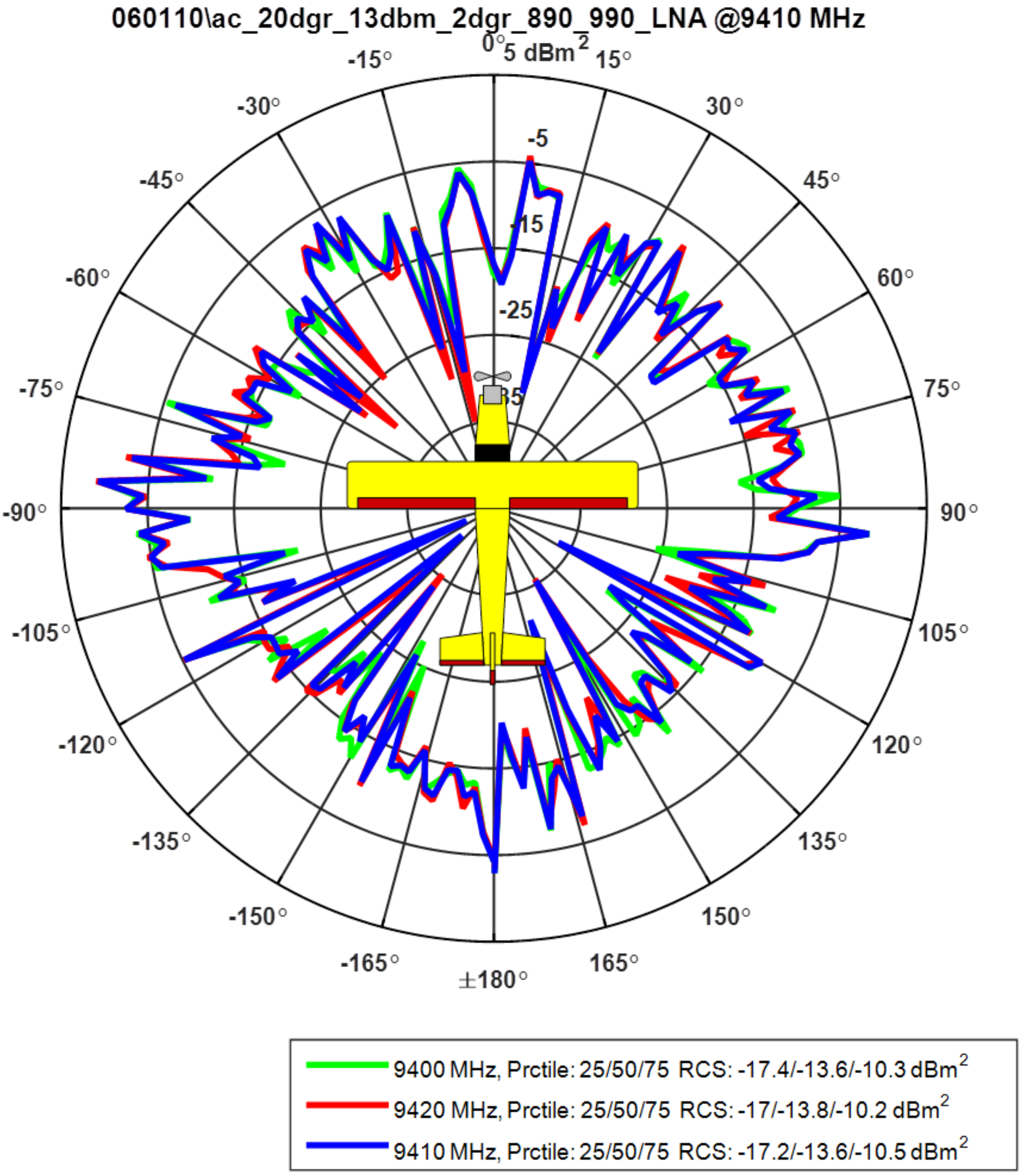

Figure E- 9 X-band RCS, horizontal polarisation, 20° elevation

Appendix F X-band RCS, vertical polarisation

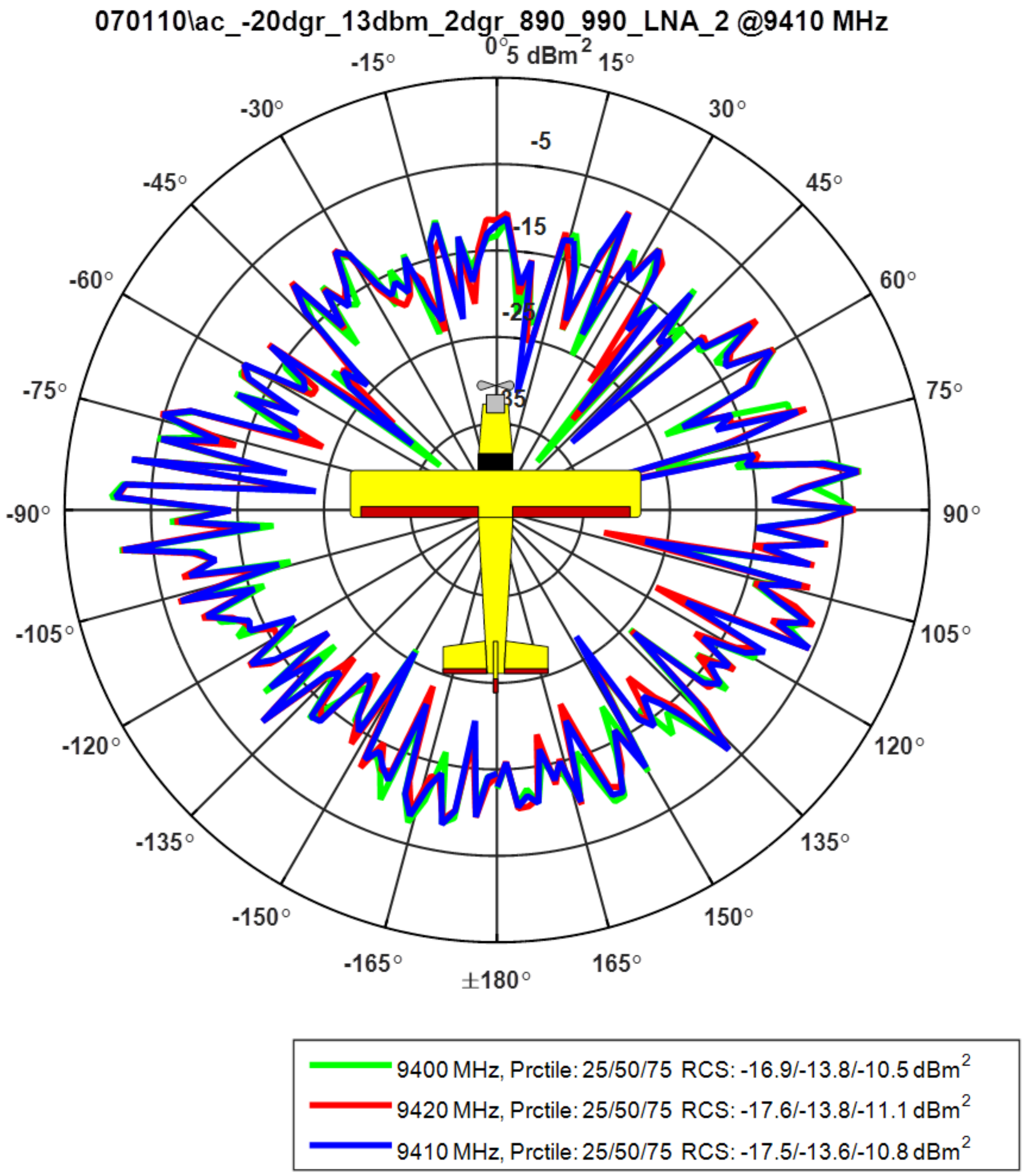

Figure F- 1 X-band RCS, vertical polarisation, -20° elevation

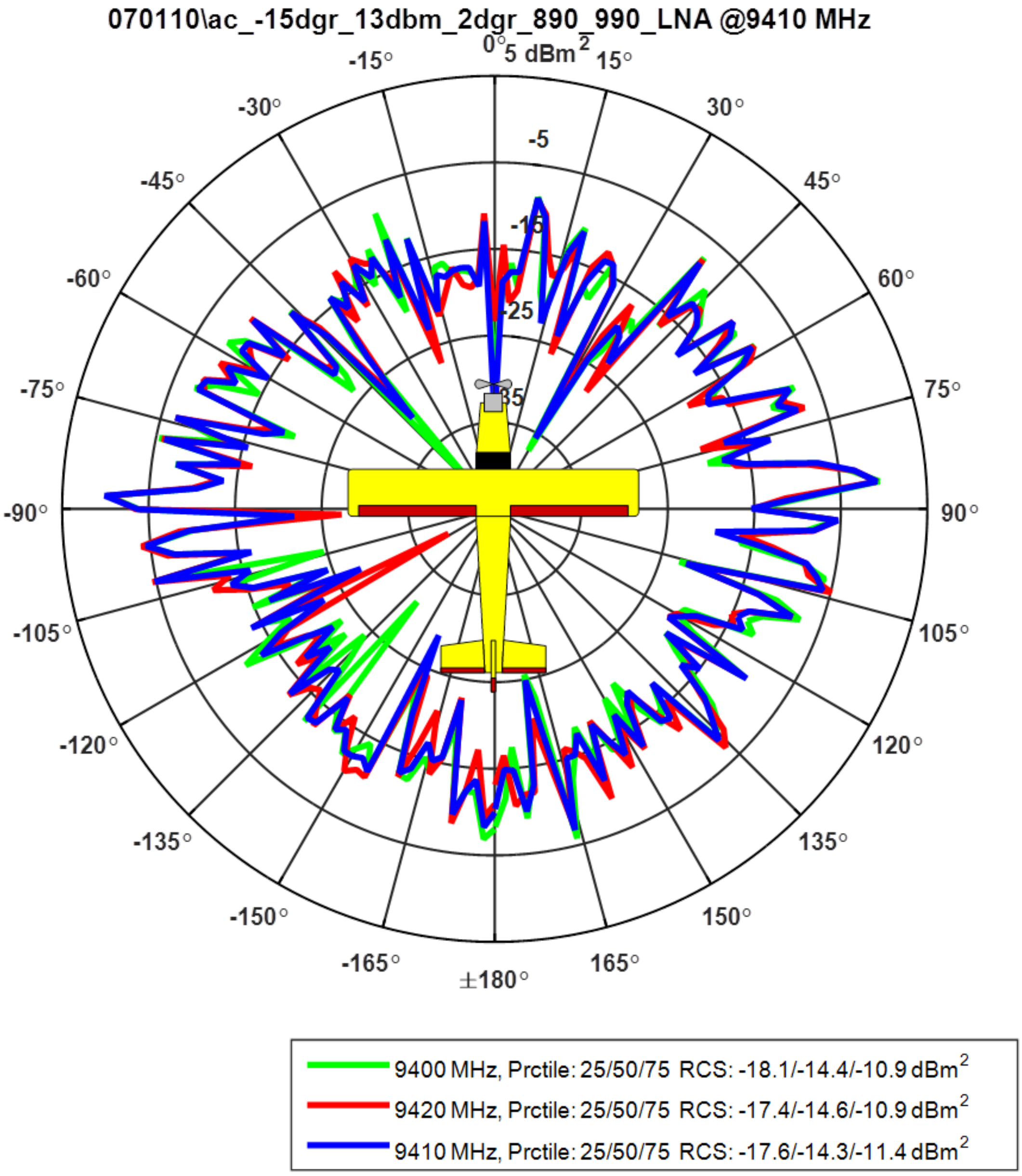

Figure F- 2 X-band RCS, vertical polarisation, -15° elevation

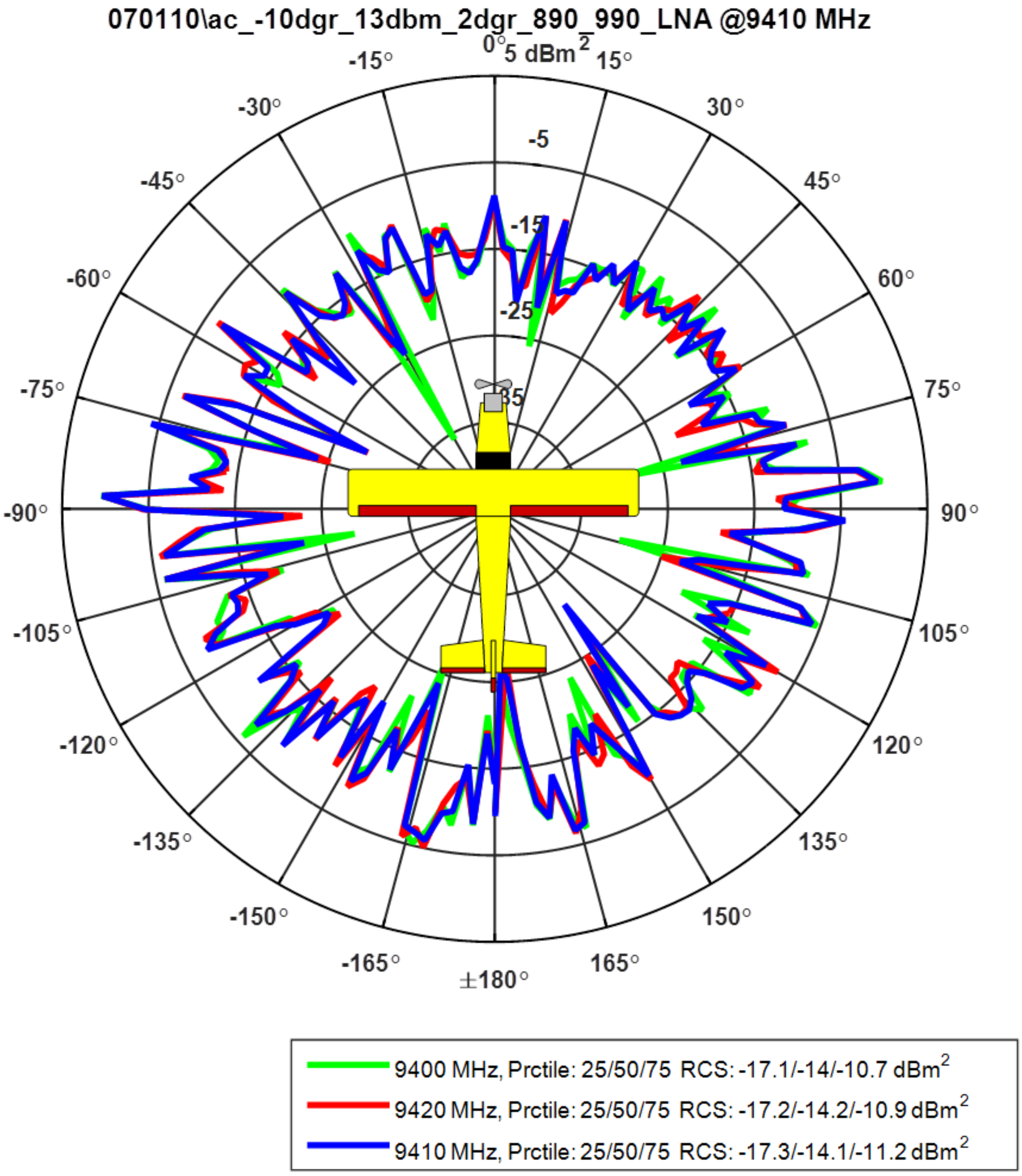

Figure F- 3 X-band RCS, vertical polarisation, -10° elevation

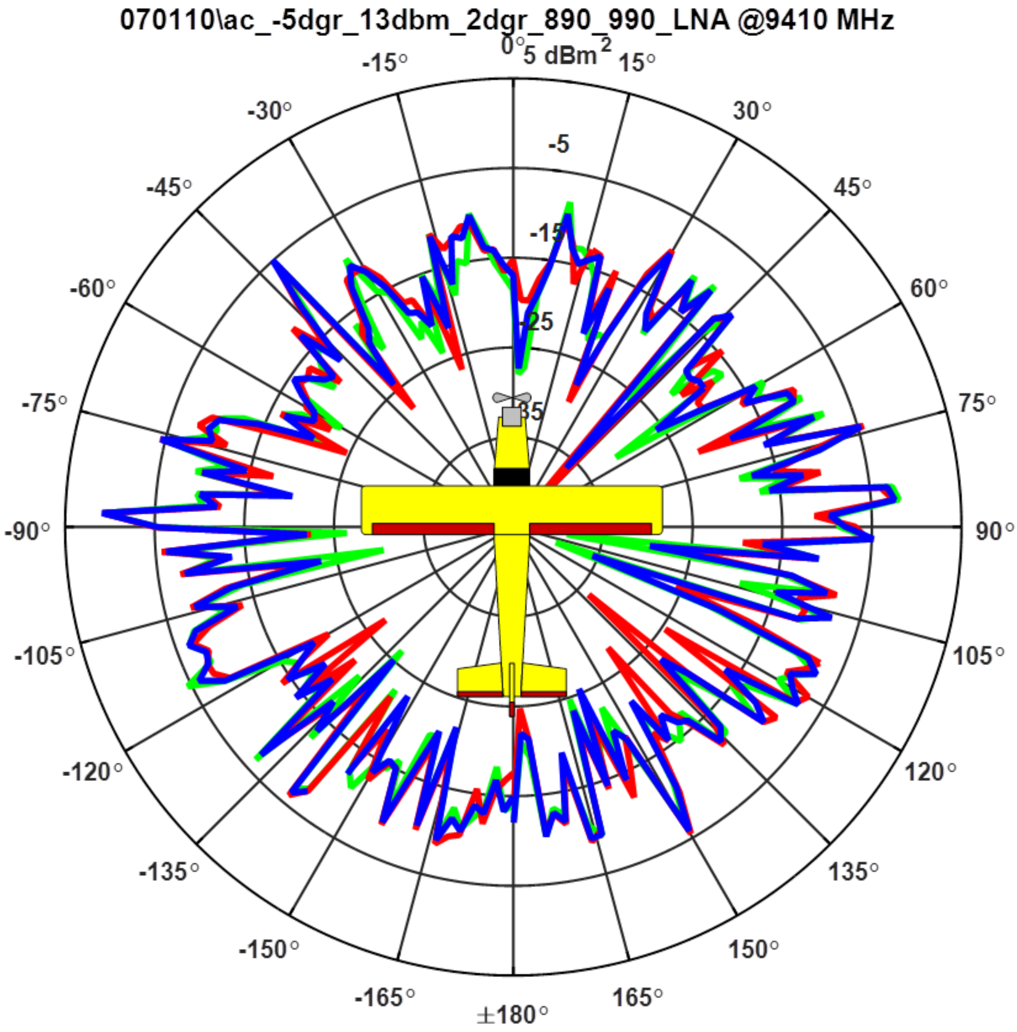

9400 MHz, Prctile: 25/50/75 RCS: -18.2/-14.6/-10.3 dBm^2
9420 MHz, Prctile: 25/50/75 RCS: -17.7/-14/-10.4 dBm^2
9410 MHz, Prctile: 25/50/75 RCS: -18.6/-14.1/-10.1 dBm^2

Figure F- 4 X-band RCS, vertical polarisation, -5° elevation

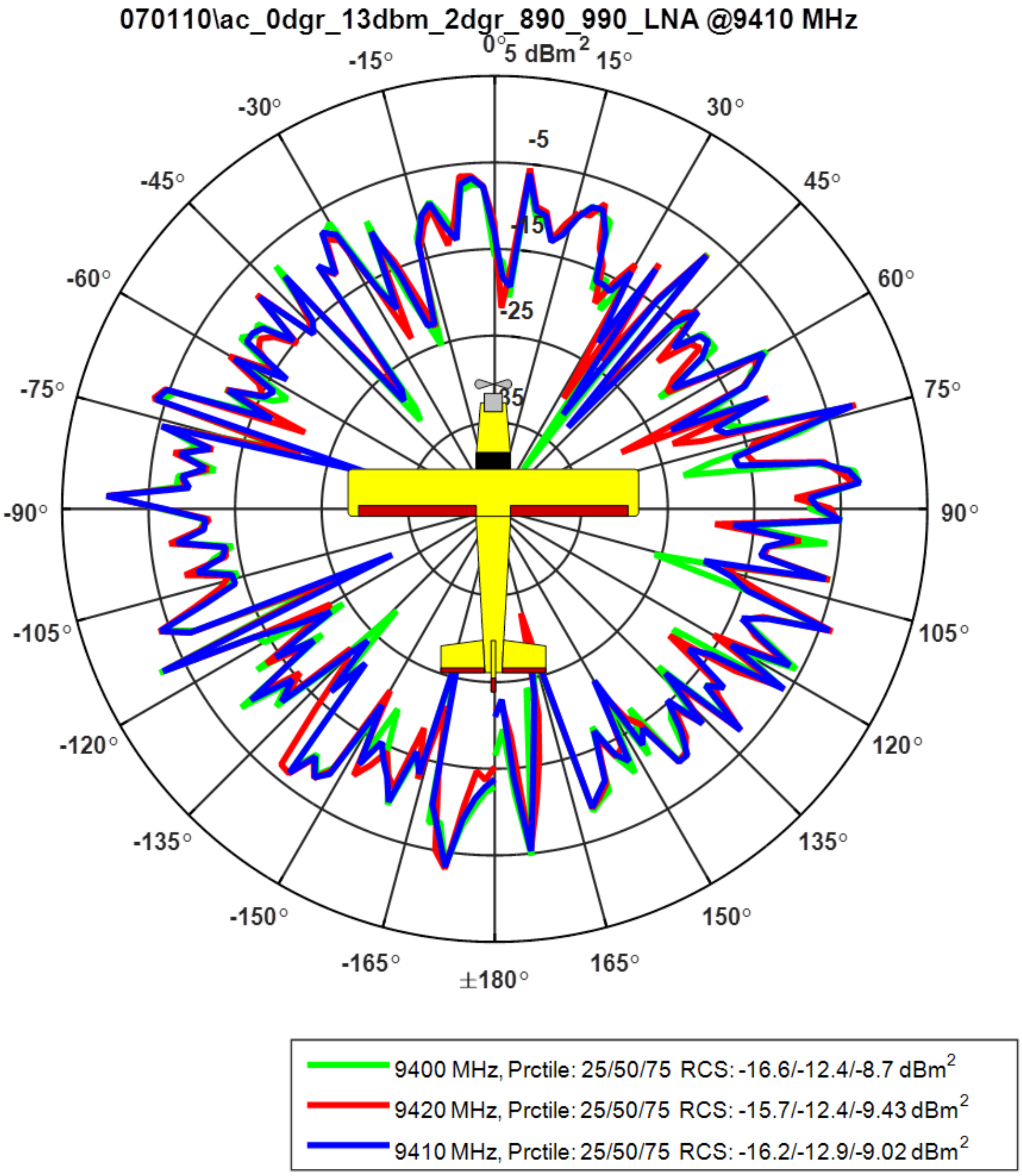

Figure F- 5 X-band RCS, vertical polarisation, 0° elevation

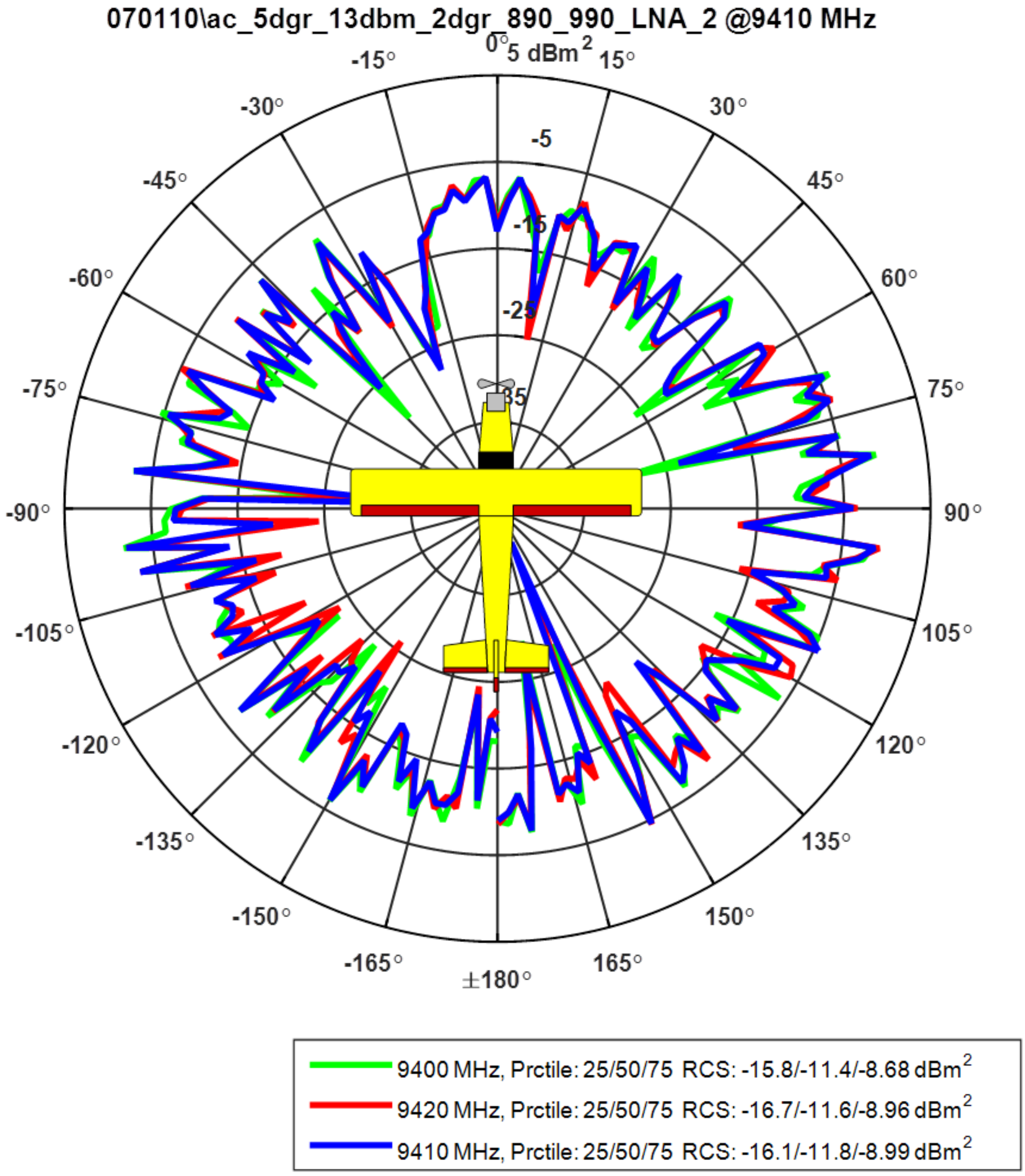

Figure F- 6 X-band RCS, vertical polarisation, 5° elevation

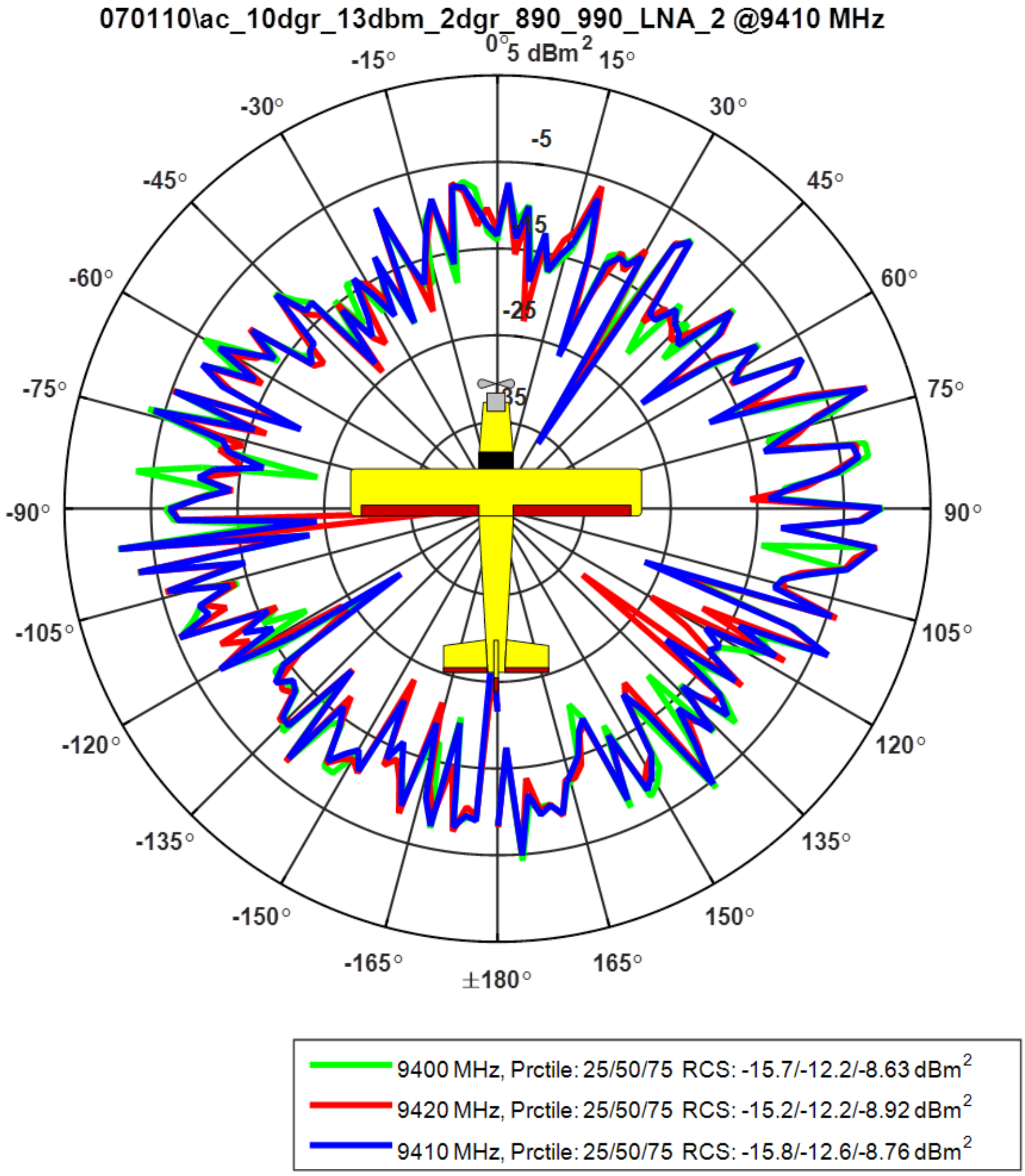

Figure F- 7 X-band RCS, vertical polarisation, 10° elevation

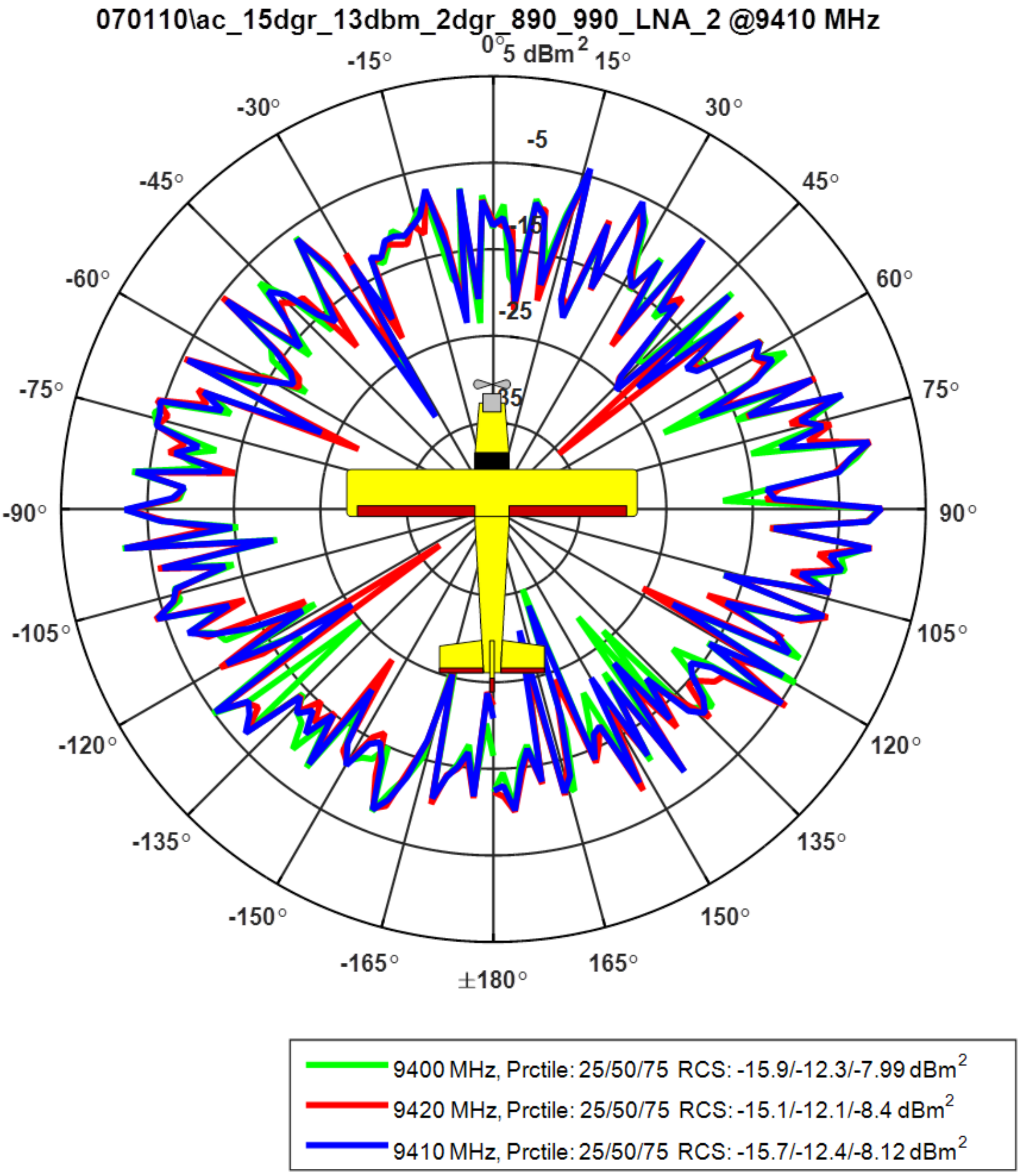

Figure F- 8 X-band RCS, vertical polarisation, 15° elevation

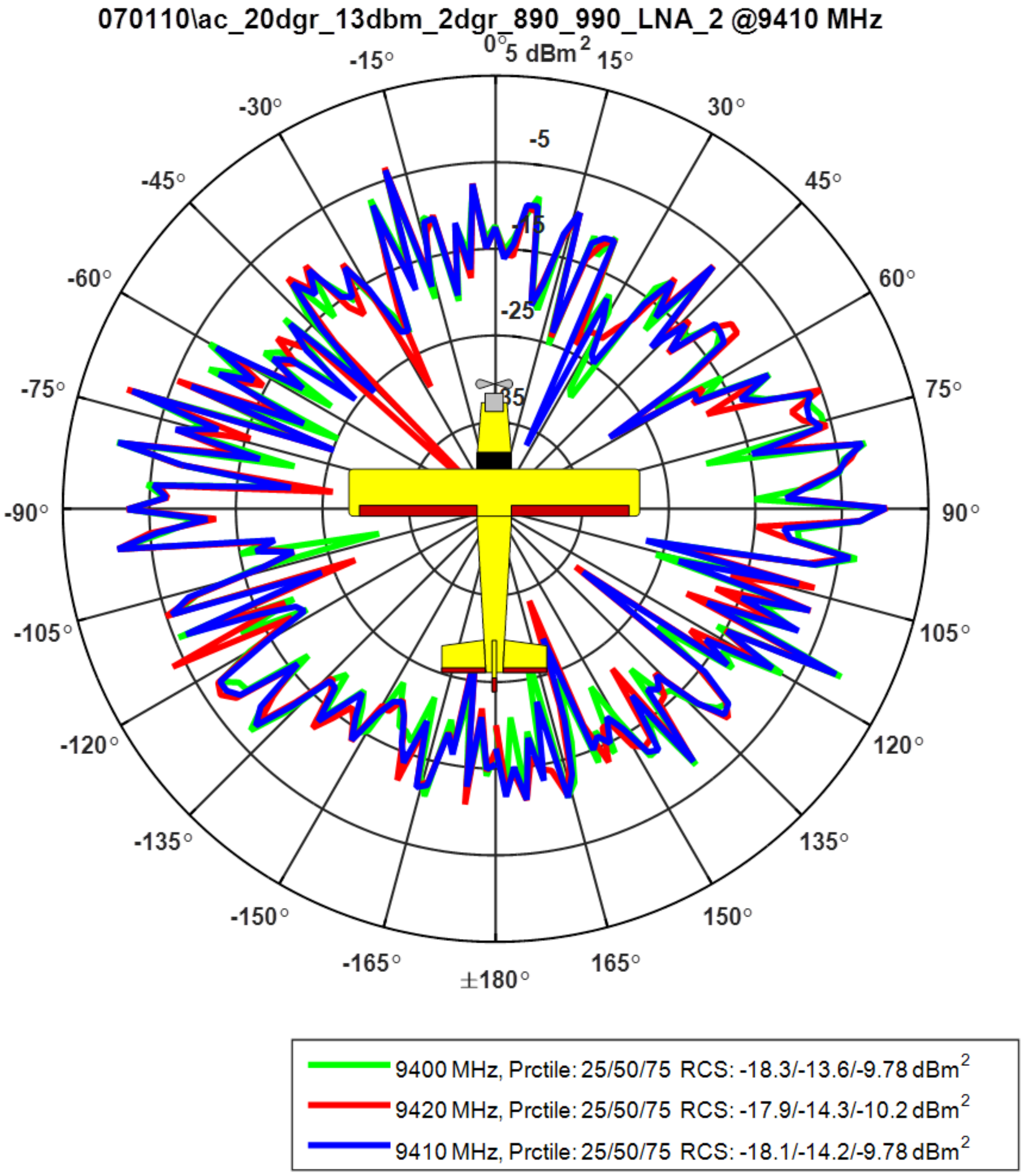

Figure F- 9 X-band RCS, vertical polarisation, 20° elevation
